# Friedel–Crafts Reaction of Acylsilanes: Highly Chemoselective Synthesis of 1-Hydroxy-bis(indolyl)methanes and 1-Silyl-bis(indolyl)methanes Derivatives

**DOI:** 10.3390/molecules28155685

**Published:** 2023-07-27

**Authors:** Qi Li, Xiu-Xia Liang, Wang Zhang, Man-Yi Han

**Affiliations:** Key Laboratory of Green and Precise Synthetic Chemistry and Applications, Ministry of Education, College of Chemistry and Materials Science, Huaibei Normal University, Huaibei 235000, China; lqq819920@126.com (Q.L.); xiuxialiang96@163.com (X.-X.L.); 13696555825@163.com (W.Z.)

**Keywords:** Friedel–Crafts reaction, bis(indolyl)methanes, hydrogen-bond, acylsilanes

## Abstract

A novel double Friedel–Crafts reaction of acylsilanes in water is described. This strategy enables synthesis of bis(indolyl)methane derivatives with 1-hydroxy or 1-silyl substituents in moderate to high yield. Compared to the 1-silyl-bis(indolyl)methane derivatives from indole substrate, 1-hydroxy-bis(indolyl)methane derivatives were synthesized from the 5-hydroxyindole, and the hydrogen bonds in the 5-hydroxyindole play a crucial role in regulating the reaction selectivity.

## 1. Introduction

**Scheme 1:**
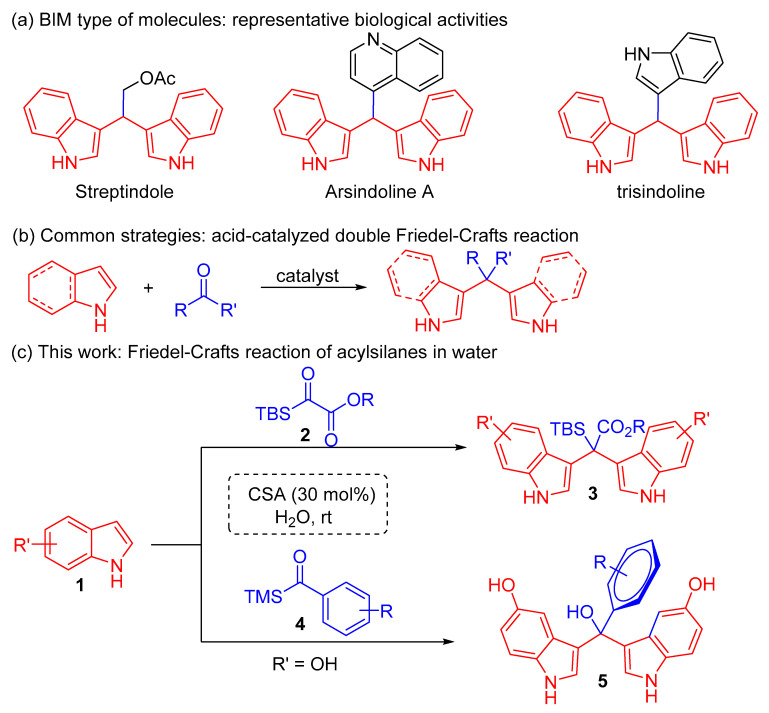
Strategies to access bis(indolyl)methanes derivatives via a double Friedel–Crafts reaction.

As an important class of indole alkaloids, bis(indolyl)methanes (BIM) broadly exist in many bioactive natural products [1,2,3,4,5], such as streptindole [6], arsindoline A [7], trisindoline [8], and a wide range of biological activities, such as antibacterial, antitumor and antileishmanial properties, were shown in these related compounds (Scheme 1a). Therefore, the synthesis of a bis(indolyl)methane (BIM) type of structure has attracted broad attention in synthetic chemistry [1,9,10,11,12,13,14,15,16]. Generally, 3-alkyl-bis(indolyl)methanes were obtained easily via a Lewis or Brønsted acid-catalyzed double Friedel–Crafts reaction between indole and carbonyl compounds (Scheme 1b). Moreover, photoredox catalysis-induced bisindolylations were also successfully employed by C-H bond cleavage [13,14]. However, the synthesis of bis(indolyl)methanes with 1-hydroxy or 1-silyl substituents remains challenging and less explored, probably due to the lesser stability of the hydroxy group and the silyl group in Lewis acid or Brønsted acid reaction conditions. Therefore, the development of an alternative synthetic method for BIM’s derivatives remains a challenging subject.

Acylsilanes are valuable organosilicon reagents in an umpolung reaction, and a C-Si or C-O bond could be synthesized by nucleophilic addition using the proton conditions or Brook rearrangement with high nucleophilicity of reagents [17,18,19,20,21,22,23,24,25,26,27,28,29]. In this regard, various reaction types, including nucleophilic addition, the carbene reaction, the Prins reaction and the Brook reaction were successfully achieved [17,18,29]. However, the Friedel–Crafts reaction of acylsilanes is less explored. Considering the unique reactivity of carbonyl groups, we wonder whether bis(indolyl)methanes with 1-silylsubstituent could be synthesized by a double Friedel–Crafts reaction of acylsilanes. Moreover, bis(indolyl)methanes with 1-hydroxy substituent could be yielded if Brook rearrangement occurred before the second Friedel–Crafts reaction. Herein, we disclose a novel double Friedel–Crafts reaction of acylsilanes, generating the bis(indolyl)methanes with 1-silyl substituent products. With the assistance of a hydrogen-bond, bis(indolyl)methanes with a 1-hydroxy substituent were successfully obtained in moderate yields (Scheme 1c).

## 2. Results

Using water as a reaction medium has been attracting considerable attention, as water is a non-toxic, green and readily available solvent [30,31,32,33,34,35]. Compared to an organic solvent, significant reaction acceleration was often observed when using water as the solvent [36,37,38,39]. Moreover, a reaction does not occur unless the solvent is water in some cases. Considering the unique ability, the Friedel–Crafts reaction of oxindole **1a** and acylsilane **2a** was firstly investigated in water (Table 1). Gratifyingly, with *p*-toluenesulfonic acid (PTSA) as the catalyst, a double Friedel–Crafts reaction occurred, yielding the desired product (**3a**) in a 43% yield. Encouraged by these results, a variety of other Brønsted acids were examined to improve the yield (Table 1, entries 2–7). A high yield of 72% for **3a** was achieved when the reaction was carried out using camphorsulfonic acid (CSA) as the catalyst (Table 1, entry 2). Compared to the strong acidic catalyst, a suitable acidic CSA is beneficial to the products. Subsequently, a series of polar or nonpolar solvents, including CH_2_Cl_2_, toluene, DMF, THF and EtOH, were investigated and inferior yields were observed (Table 1, entries 8–12). Solvent screening showed that the reaction was accelerated dramatically in water. Reducing the amount of catalyst leads to a decrease in the yield of the reaction, and even if the reaction time is extended, the yield does not increase (Table 1, entries 13–14).

**Scheme 2:**
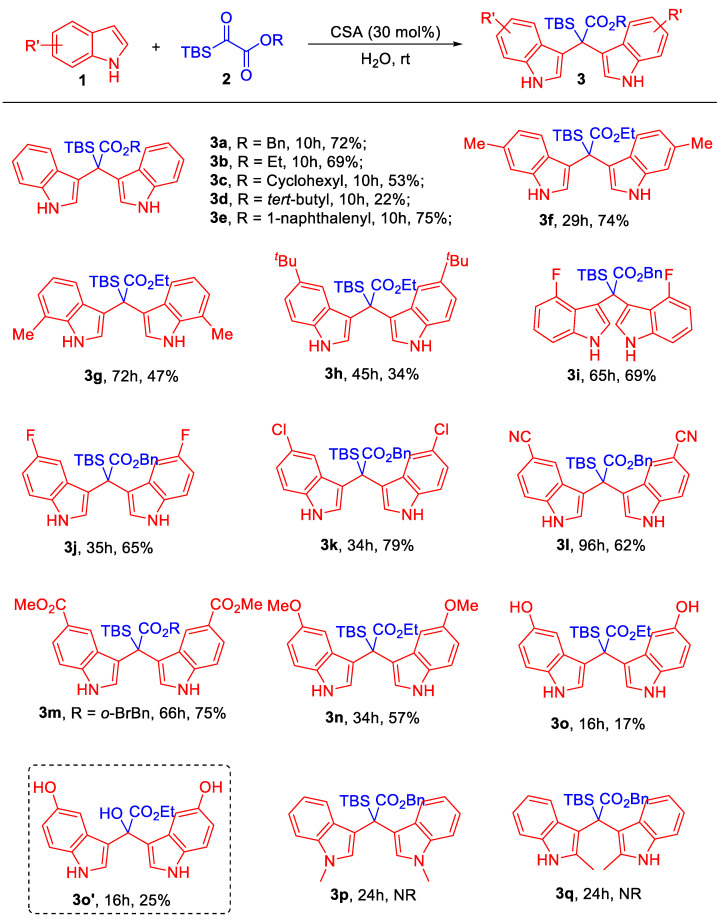
Scope of silyl glyoxylate. (Reaction conditions: **1** (0.25 mmol), silyl glyoxylate **2** (0.10 mmol), and CSA (30 mol%) in water (1.0 mL) at room temperature were stirred for the indicated time.).

With the optimized conditions in hand, we explored the scope of various silyl glyoxylates under standard reaction conditions, and various silicon-containing BIMs compounds were successfully obtained (Scheme 2). Generally, the double Friedel–Crafts reaction between indole and silyl glyoxylate containing different ester groups worked well (**3a–3o**). For example, a substrate with 1-naphthalenyl ester group was applicable, generating the desired product **3e** in 75% yield. However, a lower yield of 22% (**3d**) was observed when the substrate with *tert*-butyl ester product was employed. We speculate that the steric hindrance effect of the bulk of *tert*-butyl affects the reaction yield. Next, indoles bearing the alkyl and halogen substituents, such as Me, *tert*-butyl, F and Cl, were competent in this reaction, generating the corresponding products in moderate yield (**3f–3h**). Both electron-withdrawing (NO_2_) and electron-donating (MeO) groups attached to the indole could afford the desired products (**3l–3n**) in high yields. Interestingly, a new product of bis(indolyl)methanes with 1-hydroxy substituent (**3o’**) was formed when 5-hydroxyindole was employed as the substrate. To our delight, the hydrogen bonds from 5-hydroxyindole have a regulating effect on the generation of two products of **3o** and **3o’**. It should be noted that the desilication product of bis(indolyl)methanes with 1-hydroxy substituent products (**3o’**) was not easy to obtain by a common strategy. Moreover, no reaction was observed when 2-methylindole and 1-methylindole were used as the substrate (**3p** and **3q**) (details appear in Supplementary Materials).

**Scheme 3:**
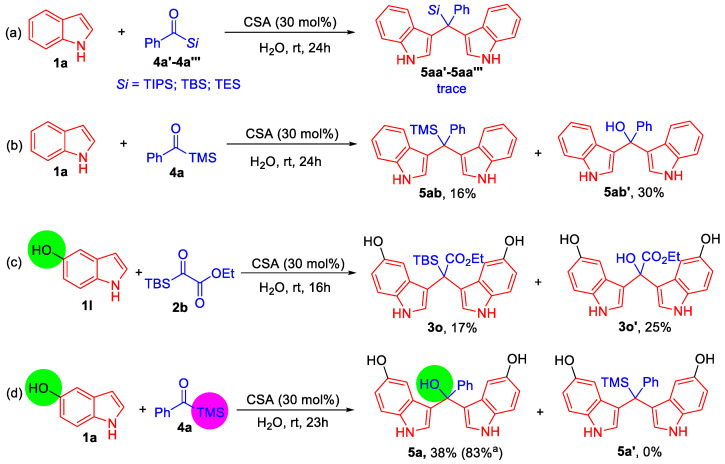
Double Friedel–Crafts reaction to synthetic tertiary alcohol. [a] The yield based on the recovery of starting materials.

To further broaden the substrate scope, the less reactive acylsilanes were investigated. However, no desired product of **5aa’–5aa’’’** was obtained when the reaction of indole (**1a**) and acylsilanes (**4a’–4a’’’**) was carried out under the optimized conditions (Scheme 3a). Considering the bulk steric hindrance effect of silyl substituents (TIPS, TBS and TES), the small size of TMS (**4a**) was employed to explore the possibility of the reactions. However, two desired products of **5ab** and **5ab’** were obtained in low yield (Scheme 3b). Based on a regulating effect of hydrogen bonds [40,41,42] (see Scheme 3c or Scheme 2), the reaction of 5-hydroxyindole was employed as the substrate under the optimized conditions. Fortunately, the desilication product of bis(indolyl)methanes with 1-hydroxy substituent product (**5a**) was obtained, and bis(indolyl)methanes with 1-silyl substituent product (**5a’**) were completely suppressed. These results clearly indicated that hydrogen bonds from the 5-hydroxyindole are a crucial factor in controlling reaction selectivity (Scheme 3d). Moreover, the hydroxyl group at different positions on indole, including 4-hydroxyindole, 6-hydroxyindole and 7-hydroxyindole, was employed as the substrate; however, a trace amount of the desired product was obtained. These results showed that 5-hydroxyindole is benefit to the desilication product. However, the exact mechanism was unclear (see SI for details).

**Scheme 4:**
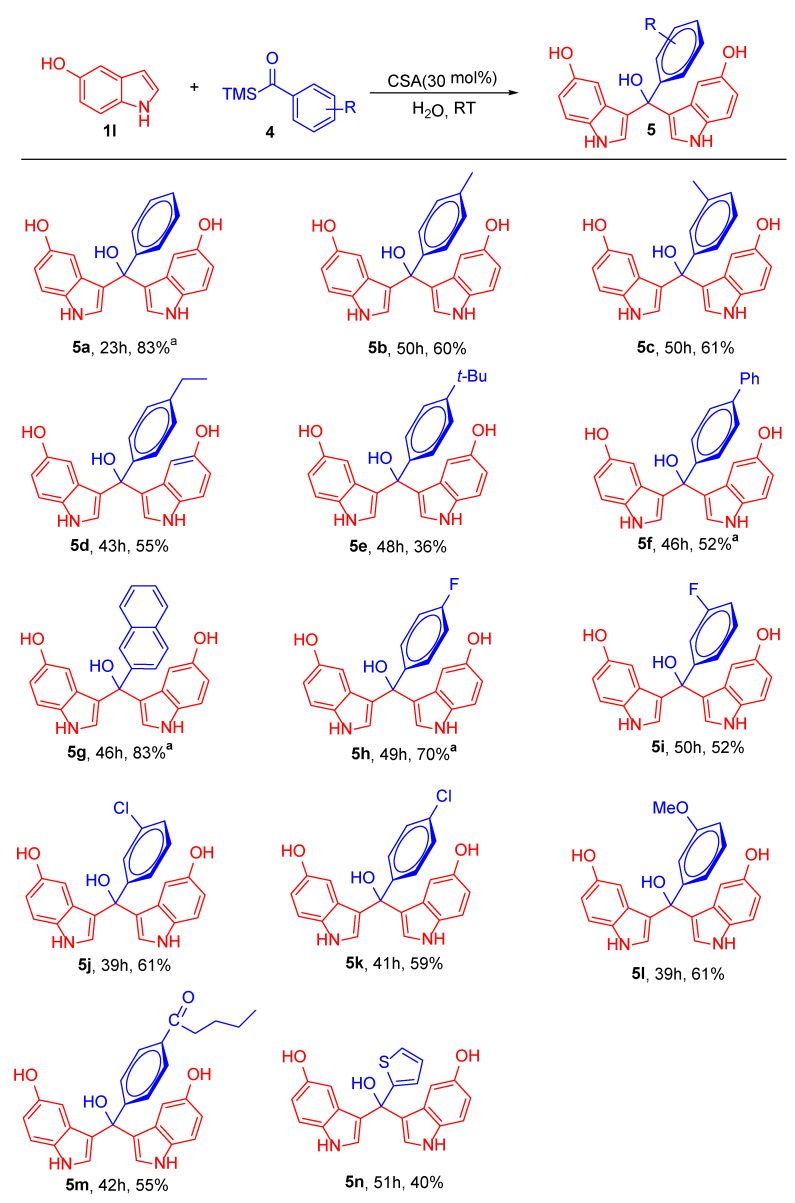
Scope of acylsilanes. (Reaction conditions: **1** (0.25 mmol), acylsilane **2** (0.10 mmol), and CSA (30 mol%) in water (1.0 mL) at room temperature were stirred for the indicated time. [a] Yield based on the recovery of starting materials. (TMS = trimethylsilyl).

Under the optimal reaction conditions, we further applied other less reactive acylsilanes to this reaction (Scheme 4). As shown in Scheme 3, the acylsilanes with an alkyl substituent, including Me, Et, tBu on the benzene ring, were amenable to this reaction, generating the desired products (**5a–5e**) in moderate yields. Moreover, the biphenyl and naphthyl substituted acylsilanes were performed smoothly with 5-hydroxyindole, affording the corresponding products (**5f** and **5g**) in good yields. Similarly, the acylsilanes with F and Cl atom on the benzene ring were also efficiently transformed into the desired products (**5i–5j**) in high yields. Both electron-withdrawing groups and electron-donating groups were also compatible with the developed protocol, yielding the corresponding products (**5l–5m**) in moderate yield. Interestingly, the thienyl-substituted acylsilane is also suitable to the reaction, and the desired product (**5n**) was obtained in moderate yield (details appear in Supplementary Materials).

**Scheme 5:**
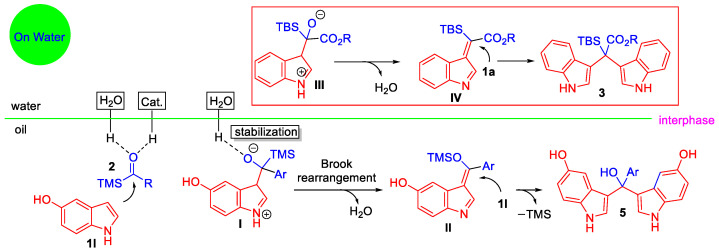
Proposed Reaction Mechanism.

Based on the experimental results, two possible reaction pathways were proposed to understand the unusual reaction selectivity. As shown in Scheme 5, the carbonyl group of acylsilanes could also be activated by CSA, and the hydrogen bond from water and the 5-hydroxyindole (**1l**) could react with the activated carbonyl group to afford the generate alkoxide intermediate **I**. Because the small size of TMS group is beneficial for Brook rearrangement, and the silyl enol ether intermediate **II** could be obtained from the intermediate **I**. These results indicate that it is more possible that the 5-hydroxyindole (**1l**) acts as a proton acid (OH) and the process occurs as an intermolecular process. Finally, another equivalent of 5-hydroxyindole (**1l**) reacts with silyl enol ether intermediate **II** to generate the desired product **5** after desilylation under the acid conditions. However, the role of the hydrogen bonding of 5-hydroxyindole is unclear in this transformation. Similarly, the carbonyl group could be activated by CSA and the hydrogen bond from water, and the indole could react with the activated carbonyl group to afford the tetrahedral intermediate **III**. Subsequently, the dehydration process is faster than the Brook rearrangement, and the azafulvene intermediate **IV** was generated by losing an equivalent amount of water. Another equivalent of **1a** reacts with intermediate **IV** to afford the final product **3**.

## 3. Materials and Methods

The detailed procedures for the synthesis and characterization of the products are given in Appendix A.

## 4. Conclusions

In summary, we developed a new strategy to synthesize bis(indolyl)methane derivatives with 1-hydroxy or 1-silyl substituents in moderate to high yield via double Friedel–Crafts reactions of acylsilanes in water. Hydrogen bonds from the 5-hydroxyindole are a crucial factor in controlling reaction selectivity between 1-silyl-bis(indolyl)methane derivatives and 1-hydroxy-bis(indolyl)methane derivatives. A variety of acylsilanes and indols were well tolerated under mild conditions. Further studies of new reactions of acylsilanes are currently underway in our laboratory.

## Figures and Tables

**Table 1:** Optimization of reaction conditions ^a^.

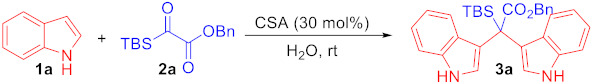				
Entry	Catalyst	Solvent	Time (h)	Yield (%) ^b^
1	PTSA	H_2_O	24	43
2	CSA	H_2_O	10	72
3	MeSO_3_H	H_2_O	24	-
4	Benzoic acid	H_2_O	70	33
5	*p*-Fluorobenzoic acid	H_2_O	69	28
6	3,5-Dinitrobenzoic acid	H_2_O	104	37
7	CH_3_CO_2_H	H_2_O	32	5
8	CSA	CH_2_Cl_2_	10	24
9	CSA	toluene	10	20
10	CSA	DMF	10	11
11	CSA	THF	10	28
12	CSA	EtOH	10	27
13	CSA ^c^	H_2_O	28	53
14	CSA ^d^	H_2_O	32	38

^a^ Reaction conditions: **1a** (0.25 mmol), **2** (0.10 mmol) in solvent (1.0 mL) at room temperature was stirred for the indicated time. ^b^ Isolated yield. ^c^ 20 mol% catalyst was used. ^d^ 10 mol% catalyst was used. TBS = *tert*-butyldimethylsily.

## Data Availability

The data presented in this study are available in this article.

## Supplementary Materials

The following supporting information can be downloaded at: https://www.mdpi.com/article/10.3390/molecules28155685/s1. Characterization data for product **3** and **5**, including ^1^H- and ^13^C-NMR spectroscopies, are available online: 1. The reaction of acylsilane with indole containing hydroxy groups at different positions; 1. ^1^H and ^13^C NMR spectra of the products.

## Sample Availability

Samples of the compounds are available from the authors.

## Appendix A. Experimental Section

Chemicals and analytical-grade solvents were purchased from commercial suppliers and used without further purification unless otherwise stated. Flash column chromatography was performed on silica gels (200–300 mesh). General ^1^H and ^13^C NMR spectra were recorded on a Bruker 600 MHz NMR spectrometer. Chemical shifts were reported in ppm, and the coupling constants *J* are given in Hz. Tetramethylsilane (TMS, *δ* = 0.00 ppm) or CHCl_3_ (*δ* = 7.27 ppm) served as an internal standard for ^1^H NMR, while CDCl_3_ was used as an internal standard (*δ* = 77.0 ppm) for ^13^C NMR. HRMS data were obtained on a Bruker Apex II mass instrument (ESI) or an Agilent Technologies 6540 UHD Accurate-Mass Q-TOF LC/MS (ESI). Silyl glyoxylates were prepared according to the literature procedure [41,42,43]. Acylsianes were prepared according to the literature procedure [28,44,45,46].

**General procedure for Friedel–Crafts reaction of silyl glyoxylates**. A mixture of indole derivative **1** (0.25 mmol), silyl glyoxylate **2** (0.10 mmol), and CSA (30 mol%) in H_2_O (1.0 mL) was stirred at room temperature for the indicated reaction time. The reaction mixture was extracted twice with 15 mL of ethyl acetate; combined organic phases were washed with brine, dried (Na_2_SO_4_), and evaporated. The residue was further purified by silica gel chromatography (petroleum ether/EtOAc as eluent) to afford the desired product **3**.

*Benzyl 2-(tert-butyldimethylsilyl)-2,2-di(1H-indol-3-yl)acetate* (**3a**); isolated by column chromatography (EtOAc/petroleum ether = 1:6); (35.6 mg, 72% yield); red amorphous solid; **^1^H NMR** (600 MHz, CDCl_3_) *δ* 8.06 (s, 2H), 7.52 (d, *J* = 2.6 Hz, 2H), 7.25 (d, *J* = 7.2 Hz, 2H), 7.17 (t, *J* = 7.2 Hz, 1H), 7.12 (t, *J* = 7.5 Hz, 2H), 6.98 (t, *J* = 7.5 Hz, 2H), 6.91 (d, *J* = 7.5 Hz, 2H), 6.80 (d, *J* = 8.1 Hz, 2H), 6.65 (t, *J* = 7.5 Hz, 2H), 5.07 (s, 2H), 0.71 (s, 9H), 0.29 (s, 6H); **^13^C NMR** (150 MHz, CDCl_3_): *δ* 175.0, 136.0, 135.8, 128.1, 127.9, 127.8, 127.6, 124.1, 121.4, 121.3, 118.8, 115.2, 110.6, 66.4, 44.6, 28.0, 20.1, −3.2; **IR** (cm^−1^) 3301, 3057, 2931, 2857, 1706, 1591, 1520, 1458, 1418; **HRMS** (ESI) *m*/*z* calcd for C_31_H_35_N_2_O_2_Si (M + H)^+^ 495.2468, found 495.2468.
*Ethyl 2-(tert-butyldimethylsilyl)-2,2-di(1H-indol-3-yl)acetate* (**3b**); isolated by column chromatography (EtOAc/petroleum ether = 1:8); (29.8 mg, 69% yield); red amorphous solid; **^1^H NMR** (600 MHz, CDCl_3_) *δ* 8.08 (s, 2H), 7.48 (s, 2H), 7.24 (d, *J* = 7.8 Hz, 2H), 6.98 (t, *J* = 7.2 Hz, 2H), 6.83 (d, *J* = 8.4 Hz, 2H), 6.68 (t, *J* = 7.2 Hz, 2H), 4.13–4.09 (m, 2H), 1.05 (t, *J* = 7.2 Hz, 3H) 0.73 (s, 9H), 0.35 (s, 6H); **^13^C NMR** (150 MHz, CDCl_3_): *δ* 175.2, 135.8, 127.8, 124.1, 121.4, 121.3, 118.7, 115.4, 110.6, 60.6, 44.4, 28.0, 20.0, 14.0, −3.1; **HRMS** (ESI) *m*/*z* calcd for C_26_H_32_N_2_O_2_SiNa (M + Na)^+^ 455.2125, found 455.2125.
*Cyclohexyl 2-(tert-butyldimethylsilyl)-2,2-di(1H-indol-3-yl)acetate* (**3c**); isolated by column chromatography (EtOAc/petroleum ether = 1:6); (25.8 mg, 53% yield); red amorphous solid; **^1^H NMR** (600 MHz, CDCl_3_) *δ* 8.07 (s, 2H), 7.46 (s, 2H), 7.24 (d, *J* = 8.4 Hz, 2H), 6.97 (t, *J* = 7.2 Hz, 2H), 6.87 (d, *J* = 8.4 Hz, 2H), 6.67 (t, *J* = 7.2 Hz, 2H), 4.84–4.85 (m, 1H), 1.58–1.68 (m, 2H), 1.28–1.37 (m, 4H), 1.13–1.21 (m, 4H), 0.70 (s, 9H), 0.39 (s, 6H); **^13^C NMR** (150 MHz, CDCl_3_): *δ* 174.6, 135.8, 127.8, 124.1, 121.2, 118.6, 115.5, 110.5, 72.7, 44.6, 31.2, 27.9, 23.2, 20.0, −2.9; **IR** (cm^−1^) 3351, 3054, 2931, 2856, 1694, 1455, 1415, 1338, 1205; **HRMS** (ESI) *m*/*z* calcd for C_30_H_38_N_2_O_2_SiNa (M + Na)^+^ 509.2595, found 509.2597.
*tert-Butyl 2-(tert-butyldimethylsilyl)-2,2-di(1H-indol-3-yl)acetate* (**3d**); isolated by column chromatography (EtOAc/petroleum ether = 1:5); (10.1 mg, 22% yield); red amorphous solid; **^1^H NMR** (600 MHz, CDCl_3_) *δ* 8.04 (s, 2H), 7.38 (s, 2H), 7.26 (d, *J* = 1.4 Hz, 2H), 7.25 (s, 1H), 6.98 (t, *J* = 7.5 Hz, 2H), 6.89 (d, *J* = 8.0 Hz, 1H), 6.68 (t, *J* = 7.6 Hz, 2H), 1.25 (s, 9H), 0.61 (s, 9H), 0.42 (s, 6H); **^13^C NMR** (150 MHz, CDCl_3_): *δ* 174.0, 135.9, 127.8, 124.0, 122.0, 121.2, 118.6, 115.8, 110.5, 80.4, 45.3, 27.9, 27.8, 19.9, −2.8; **HRMS** (ESI) *m*/*z* calcd for C_28_H_36_N_2_O_2_SiNa (M + Na)^+^ 483.2444, found 483.2444.
*Naphthalen-1-yl 2-(tert-butyldimethylsilyl)-2,2-di(1H-indol-3-yl)acetate* (**3e**); isolated by column chromatography (EtOAc/petroleum ether = 1:6); (39.8 mg, 75% yield); red amorphous solid; **^1^H NMR** (600 MHz, CDCl_3_) *δ* 8.01 (s, 2H), 7.72 (dd, *J* = 17.6, 8.2 Hz, 2H), 7.49 (s, 2H), 7.35 (t, *J* = 7.5 Hz, 1H), 7.25 (d, *J* = 8.1 Hz, 3H), 7.21 (d, *J* = 8.5 Hz, 1H), 7.18 (d, *J* = 7.0 Hz, 1H), 7.05 (t, *J* = 7.6 Hz, 1H), 6.99 (t, *J* = 7.5 Hz, 2H), 6.86 (d, *J* = 8.2 Hz, 2H), 6.66 (t, *J* = 7.6 Hz, 2H), 5.48 (s, 2H), 0.69 (s, 9H), 0.23 (s, 6H); **^13^C NMR** (150 MHz, CDCl_3_): *δ* 175.3, 135.8, 133.3, 131.5, 131.4, 128.8, 128.1, 127.8, 127.0, 125.9, 125.5, 124.9, 124.1, 123.7, 121.33, 121.27, 118.8, 115.1, 110.6, 65.2, 44.6, 27.9, 20.0, −3.3; **HRMS** (ESI) *m*/*z* calcd for C_35_H_36_N_2_O_2_SiNa (M + Na)^+^ 567.2438, found 567.2442.
*Ethyl 2-(tert-butyldimethylsilyl)-2,2-bis(6-methyl-1H-indol-3-yl)acetate* (**3f**); isolated by column chromatography (EtOAc/petroleum ether = 1:5); (34.0 mg, 74% yield); red amorphous solid; **^1^H NMR** (600 MHz, CDCl_3_) *δ* 7.92 (s, 2H), 7.38 (d, *J*= 2.4 Hz, 2H), 7.02–7.00 (s, 2H), 6.74 (d, *J* = 8.4 Hz, 2H), 6.52 (dd, *J* = 7.8 Hz, 1.8 Hz, 2H), 4.10 (q, *J* = 7.2 Hz, 2H), 1.06 (t, *J* = 7.2 Hz, 3H), 0.73 (s, 9H), 0.34 (s, 6H); **^13^C NMR** (150 MHz, CDCl_3_): *δ* 175.2, 136.3, 130.8, 125.7, 123.5, 121.1, 120.6, 115.3, 110.6, 60.6, 44.4, 28.0, 21.5, 20.0, 14.1, −3.0; **HRMS** (ESI) *m*/*z* calcd for C_28_H_36_N_2_O_2_SiNa (M + Na)^+^ 483.2438, found 483.2435.
*Ethyl 2-(tert-butyldimethylsilyl)-2,2-bis(7-methyl-1H-indol-3-yl)acetate* (**3g**); isolated by column chromatography (EtOAc/petroleum ether = 1:5); (21.6 mg, 47% yield); red amorphous solid; **^1^H NMR** (600 MHz, CDCl_3_) *δ* 7.99 (s, 2H), 7.49 (d, *J* = 3.0 Hz, 2H), 6.78 (d, *J* = 4.8 Hz, 2H), 6.70 (d, *J* = 2.4 Hz, 2H), 6.61 (dd, *J* = 7.2 Hz, 6.6 Hz, 2H), 4.10 (q, *J* = 7.2 Hz, 2H), 2.43 (s, 6H), 1.06 (t, *J* =7.2 Hz, 3H), 0.73 (s, 9H), 0.34 (s, 6H); **^13^C NMR** (150 MHz, CDCl_3_): *δ* 175.2, 135.4, 127.3, 123.9, 121.9, 119.4, 119.3, 118.8, 116,0, 60.6, 44.6, 28.1, 20.0, 16.5, 14.1, −3.0; **HRMS** (ESI) *m*/*z* calcd for C_28_H_36_N_2_O_2_Si (M + H)^+^ 461.2624, found 461.2622.
*Ethyl 2,2-bis(5-(tert-butyl)-1H-indol-3-yl)-2-(tert-butyldimethylsilyl)acetate* (**3h**); isolated by column chromatography (EtOAc/petroleum ether = 1:6); (18.5 mg, 34% yield); red amorphous solid; **^1^H NMR** (600 MHz, CDCl_3_) *δ* 7.98 (s, 2H), 7.54 (d, *J* = 2.4 Hz, 2H), 7.15 (d, *J* = 8.4 Hz, 2H), 7.00 (dd, *J* = 8.4 Hz, 1.8 Hz, 2H), 6.67 (s, 2H), 4.11 (q, *J* = 7.2 Hz, 2H), 1.08 (t, *J* = 7.2 Hz, 3H), 0.98 (s, 18H), 0.77 (s, 9H), 0.35 (s, 6H); **^13^C NMR** (150 MHz, CDCl_3_) *δ* 175.4, 140.8, 134.1, 127.8, 124.11, 119.0, 118.0, 115.2, 109.5, 60.5, 44.4, 34.1, 31.6, 28.2, 20.1, 14.1, −3.1; **HRMS** (ESI) *m/z* calcd for C_34_H_48_N_2_O_2_SiNa (M + Na)^+^ 567.3377, found 567.3379.
*Benzyl 2-(tert-butyldimethylsilyl)-2,2-bis(4-fluoro-1H-indol-3-yl)acetate* (**3i**); isolated by column chromatography (EtOAc/petroleum ether = 1:5); (38.8 mg, 69% yield); red amorphous solid; **^1^H NMR** (600 MHz, CDCl_3_) *δ* 8.15 (s, 2H), 7.76 (s, 2H), 7.13–7.16 (m, 1H), 7.08–7.11 (m, 2H), 7.03 (d, *J* = 7.8 Hz, 2H), 6.90–6.86 (m, 4H), 6.35 (q, *J* = 7.8 Hz, 2H), 5.11 (s,2H), 0.94 (s, 9H), 0.26 (s, 6H); **^13^C NMR** (150 MHz, CDCl_3_): *δ* 175.1, 156.2 (d, *J*_C-F_ = 246 Hz), 138.8 (d, *J*_C-F_ = 12 Hz), 135.9, 128.0, 127.6, 127.4, 124.8 (d, *J*_C-F_ = 3 Hz), 121.5 (d, *J*_C-F_ = 9 Hz), 116.1 (d, *J*_C-F_ = 19.5 Hz), 115.6 (d, *J*_C-F_ = 4.5 Hz), 106.9 (d, *J*_C-F_ = 12 Hz), 104.9 (d, *J*_C-F_ = 22.5 Hz), 66.6, 44.9, 28.7, 20.3, −3.5; **IR** (cm^−1^) 3469, 3034, 2931, 2858, 1698, 1574, 1498, 1471, 1223; **HRMS** (ESI) *m*/*z* calcd for C_31_H_33_F_2_N_2_O_2_Si (M + H)^+^ 531.2279, found 531.2276.
*Benzyl 2-(tert-butyldimethylsilyl)-2,2-bis(5-fluoro-1H-indol-3-yl)acetate* (**3j**); isolated by column chromatography (EtOAc/petroleum ether = 1:6); (34.5 mg, 65% yield); red amorphous solid; **^1^H NMR** (600 MHz, CDCl_3_) *δ* 8.12 (s, 2H), 7.50 (d, *J* = 2.5 Hz, 2H), 7.20–7.16 (m, 1H), 7.16–7.12 (m, 2H), 6.94–6.90 (m, 4H), 6.64 (dd, *J* = 9.0, 5.4 Hz, 2H), 6.64–6.38 (m, 2H), 5.08 (s, 2H), 0.72 (s, 9H), 0.27 (s, 6H); **^13^C NMR** (150 MHz, CDCl_3_): *δ* 174.8, 159.4 (d, *J*_C-F_ = 237 Hz), 154.2 (d, *J*_C-F_ = 64.5 Hz), 135.8, 128.0 (d, *J*_C-F_ = 28.5 Hz), 127.7, 124.3 (d, *J*_C-F_ = 3 Hz), 124.2, 122.0 (d, *J*_C-F_ = 9 Hz), 115.1, 107.7 (d, *J*_C-F_ = 24 Hz), 96.8 (d, *J*_C-F_ = 25.5 Hz), 66.5, 66.5, 44.4, 27.9, 20.0, 1.0, −3.3; **HRMS** (ESI) *m*/*z* calcd for C_31_H_33_F_2_N_2_O_2_Si (M + H)^+^ 531.2279, found 531.2274.
*Benzyl 2-(tert-butyldimethylsilyl)-2,2-bis(5-chloro-1H-indol-3-yl)acetate* (**3k**); isolated by column chromatography (EtOAc/petroleum ether = 1:5); (44.4 mg, 79% yield); red amorphous solid; **^1^H NMR** (600 MHz, CDCl_3_) *δ* 8.15 (s, 2H), 7.57 (s, 2H), 7.20–7.13 (m, 5H), 6.97 (d, *J* = 7.3 Hz, 2H), 6.93 (d, *J* = 8.7 Hz, 2H), 6.69 (s, 2H), 5.10 (s, 2H), 0.75 (s, 9H), 0.28 (s, 6H); **^13^C NMR** (150 MHz, CDCl_3_): *δ* 174.7, 135.6, 134.2, 128.6, 128.2, 128.1, 127.9, 125.5, 124.5, 121.9, 120.4, 114.6. 111.9, 66.8, 44.2, 28.0, 20.1, −3.3; **IR** (cm^−1^) 3468, 3032, 2933, 2858, 1705, 1565, 1463, 1412, 1257, 1189; **HRMS** (ESI) *m*/*z* calcd for C_31_H_32_C_l2_N_2_O_2_SiNa (M + Na)^+^ 585.1502, found 585.1508.
*Benzyl 2-(tert-butyldimethylsilyl)-2,2-bis(5-cyano-1H-indol-3-yl)acetate* (**3l**); isolated by column chromatography (EtOAc/petroleum ether = 1:6); (33.7 mg, 62% yield); red amorphous solid; **^1^H NMR** (600 MHz, DMSO-d_6_) *δ* 11.65 (d, *J* = 1.8 Hz, 2H), 7.79 (d, *J* = 2.4 Hz, 2H), 7.50 (d, *J* = 8.4 Hz, 2H), 7.23 (dd, *J* = 8.4 Hz, 1.8 Hz, 2H), 7.22–7.18 (m, 1H), 7.15 (t, *J* = 7.8 Hz, 2H), 7.00–6.93 (m, 2H), 6.75 (s, 2H), 5.09 (s, 2H), 0.66 (s, 9H), 0.24 (s, 6H); **^13^C NMR** (150 MHz, DMSO-d_6_): *δ* 173.8, 137.8, 135.7, 128.1, 127.94, 127.91, 127.6, 126.8, 125.3, 123.3, 120.6, 114.2, 113.0, 100.0, 66.2, 43.6, 27.7, 19.7, −3.6; **HRMS** (ESI) *m*/*z* calcd for C_33_H_32_N_4_O_2_SiNa (M + Na)^+^ 567.2192, found 567.2193.
*Dimethyl 3,3’-(2-((2-bromobenzyl)oxy)-1-(tert-butyldimethylsilyl)-2-oxoethane-1,1-diyl)bis(1H-indole-6-carboxylate)* (**3m**); isolated by column chromatography (EtOAc/petroleum ether = 1:6); (51.6 mg, 75% yield); red amorphous solid; **^1^H NMR** (600 MHz, CDCl_3_) *δ* 8.83 (d, *J* = 2.4 Hz, 2H), 8.06 (d, *J* = 1.2 Hz, 2H), 7.79 (d, *J* = 2.4 Hz, 2H), 7.32 (dd, *J* = 1.2, 7.8 Hz, 1H), 7.26 (dd, *J* = 1.2, 7.8 Hz, 2H), 6.95 (td, *J* = 1.8, 7.2 Hz, 1H), 6.86 (td, *J* = 1.8, 7.2 Hz, 1H), 6.67 (d, *J* = 9.0 Hz, 2H), 6.63 (dd, *J* = 1.8, 7.8 Hz, 1H), 5.16 (s 2H), 3.83 (s, 6H), 0.72 (s, 9H), 0.31 (s, 6H); **^13^C NMR** (150 MHz, CDCl_3_): *δ* 174.5, 168.2, 135.2, 134.9, 132.3, 131.2, 129.4, 129.2, 127.7, 127.0, 122.9, 122.8, 120.5, 119.8, 115.2, 113.4, 65.9, 51.8, 44.4, 27.9, 20.0, −3.4; **HRMS** (ESI) *m*/*z* calcd for C_31_H_33_BrN_2_O_2_SiNa (M + Na)^+^ 595.1387, found 595.1389.
*Ethyl 2-(tert-butyldimethylsilyl)-2,2-bis(5-methoxy-1H-indol-3-yl)acetate* (**3n**); isolated by column chromatography (EtOAc/petroleum ether = 1:6); (28.0 mg, 57% yield); red amorphous solid; **^1^H NMR** (600 MHz, DMSO-d_6_) *δ* 10.66 (s, 2H), 7.33 (d, *J* = 2.4 Hz, 2H), 6.74 (d, *J* = 1.8 Hz, 2H), 6.43 (d, *J* = 9.0 Hz, 2H), 6.29 (dd, *J* = 9.0 Hz, 2.4 Hz, 2H), 3.99 (q, *J* = 7.2 Hz, 2H), 3.65 (s, 6H), 1.00 (t, *J* = 7.2 Hz, 3H), 0.65 (s, 9H), 0.25 (s, 6H); **^13^C NMR** (150 MHz, DMSO-d_6_): *δ* 174.5, 154.7, 136.5, 123.2, 121.8, 121.0, 113.6, 107.8, 94.0, 59.9, 54.8, 43.7, 27.8, 19.7, 14.0, −3.3; **HRMS** (ESI) *m*/*z* calcd for C_28_H_36_N_2_O_4_SiNa (M + Na)^+^ 515.2337, found 515.2339.
*Ethyl 2-(tert-butyldimethylsilyl)-2,2-bis(5-hydroxy-1H-indol-3-yl)acetate* (**3o**); isolated by column chromatography (EtOAc/petroleum ether = 1:8); (7.9 mg, 17% yield); red amorphous solid; **^1^H NMR** (600 MHz, DMSO-d_6_) *δ* 10.53 (s, 2H), 8.16 (s, 2H), 7.30 (s, 2H), 7.03 (d, *J* = 8.4 Hz, 2H), 6.40 (d, *J* = 8.4 Hz, 2H), 6.08 (s, 2H), 4.00 (q, *J* = 7.2 Hz, 2H), 1.02 (t, *J* = 7.2 Hz, 3H), 0.63 (s, 9H), 0.26 (s, 6H); **^13^C NMR** (150 MHz, DMSO-d_6_): *δ* 174.5, 149.0, 130.5, 128.2, 125.2, 112.6, 111.0, 110.7, 105.2, 59.8, 43.7, 27.9, 19.7, 14.0, −2.9; **HRMS** (ESI) *m*/*z* calcd for C_26_H_32_N_2_O_4_SiNa (M + Na)^+^ 487.2024, found 487.2026.
*Ethyl 2-(tert-butyldimethylsilyl)-2,2-bis(5-hydroxy-1H-indol-3-yl)acetate* (**3o’**); isolated by column chromatography (EtOAc/petroleum ether = 1:5); (9.2 mg, 25% yield); red amorphous solid; **^1^H NMR** (600 MHz, DMSO-d_6_) *δ* 10.62 (s, 2H), 8.59 (s, 2H), 7.13 (d, *J* = 8.6 Hz, 2H), 7.03 (s, 2H), 6.81 (s, 2H), 6.58 (d, *J* = 8.4 Hz, 2H), 5.14 (s, 1H), 4.10 (q, *J* = 7.2 Hz, 2H), 1.18 (t, *J* = 7.2 Hz, 3H); **^13^C NMR** (150 MHz, DMSO-d_6_): *δ* 172.8, 150.2, 130.9, 127.1, 124.1, 111.8, 111.4, 111.3, 102.9, 60.2, 40.6, 14.2; **HRMS** (ESI) *m*/*z* calcd for C_20_H_18_N_2_O_5_ (M-(H_2_O) + H)^+^ 349.1188, found 349.1184.
*3,3’-(Phenyl(trimethylsilyl)methylene)bis(1H-indole)* (**5ab**); isolated by column chromatography (EtOAc/petroleum ether = 1:6); (6.3 mg, 16% yield); red amorphous solid; **^1^H NMR** (600 MHz, CDCl_3_) *δ* 7.96 (s, 2H), 7.37 (d, *J* = 7.8 Hz, 2H), 7.33 (d, *J* = 8.1 Hz, 2H), 7.23 (t, *J* = 7.6 Hz, 2H), 7.16 (t, *J* = 7.2 Hz, 1H), 7.08 (t, *J* = 7.6 Hz, 2H), 7.00 (d, *J* = 8.2 Hz, 2H), 6.89 (s, 2H), 6.80 (t, *J* = 7.6 Hz, 2H), 0.14 (s, 9H); **^13^C NMR** (150 MHz, CDCl_3_): *δ* 146.1, 136.7, 129.4, 127.6, 127.4, 125.1, 123.8, 122.9, 121.4, 121.3, 118.5, 110.9, 1.0; **HRMS** (ESI) *m*/*z* calcd for C_26_H_26_N_2_SiNa (M + Na)^+^ 417.1758, found 417.1755.
*Di(1H-indol-3-yl)(phenyl)methanol* (**5ab’**); isolated by column chromatography (EtOAc/petroleum ether = 1:8); (10.1 mg, 30% yield); red amorphous solid; **^1^H NMR** (600 MHz, CDCl_3_) *δ* 7.87 (s, 2H), 7.40 (d, *J* = 7.8 Hz, 2H), 7.34–7.36 (m, 4H), 7.29 (t, *J* = 7.5 Hz, 2H), 7.21–7.23 (m, 1H), 7.16–7.19 (m, 2H), 7.01 (t, *J* = 7.5 Hz, 2H), 6.64 (s, 2H), 5.90 (s, 1H); **^13^C NMR** (150 MHz, CDCl_3_):*δ* 144.0, 136.7, 128.7, 128.2, 127.0, 126.1, 123.6, 121.9, 119.9, 119.7, 119.2, 111.0, 40.2; **HRMS** (ESI) *m*/*z* calcd for C_23_H_17_N_2_ (M-(H_2_O) + H)^+^ 321.1391, found 321.1395.

**General procedure for Friedel–Crafts reaction of acylsilanes.** A mixture of acylsianes **3** (0.10 mmol), 5-hydroxyindole **1l** (0.25 mmol) and CSA (30 mol%) in H_2_O (1.0 mL) was stirred at room temperature for the indicated time. Then, the mixture was extracted twice with EtOAc (10 mL), and the combined organic phases were washed with brine, dried (Na_2_SO_4_) and evaporated. The residue was further purified by silica gel chromatography (petroleum ether/EtOAc as eluent) to afford the desired product **5**.

*3,3’-(Hydroxy(phenyl)methylene)bis(1H-indol-5-ol)* (**5a**); isolated by column chromatography (EtOAc/petroleum ether = 1:5); (14.1 mg, 83% yield); red amorphous solid; **^1^H NMR** (600 MHz, acetone-d_6_) *δ* 9.70 (s, 2H), 7.61 (s, 2H), 7.41–7.39 (m, 2H), 7.28 (t, *J* = 7.6 Hz, 2H), 7.23 (d, *J* = 8.7 Hz, 2H), 7.19 (t, *J* = 7.4 Hz, 1H), 6.79 (d, *J* = 2.3 Hz, 2H), 6.72 (dd, *J* = 8.3, 2.5 Hz, 4H), 5.73 (s, 1H); **^13^C NMR** (150 MHz, acetone-d_6_):*δ* 151.2, 145.8, 132.8, 129.5, 128.79, 128.76, 126.6, 125.2, 118.9, 112.5, 112.2, 104.5, 41.2; **HRMS** (ESI) *m*/*z* calcd for C_23_H_17_N_2_O_2_ (M-(H_2_O) + H)^+^ 353.1289, found 353.1285.
*3,3’-(Hydroxy(p-tolyl)methylene)bis(1H-indol-5-ol)* (**5b**); isolated by column chromatography (EtOAc/petroleum ether = 1:5); (23.0 mg, 60% yield); red amorphous solid; **^1^H NMR** (600 MHz, acetone-d_6_) *δ* 9.68 (s, 2H), 7.53 (s, 2H), 7.26 (d, *J* = 8.4 Hz, 2H), 7.21 (d, *J* = 8.4 Hz, 2H), 7.08 (d, *J* = 7.8 Hz, 2H), 6.76 (d, *J* = 2.4 Hz, 2H), 6.70 (t, *J* = 1.8 Hz, 3H), 6.68 (d, *J* = 2.4 Hz, 1H), 5.66 (s, 1H), 2.29 (s, 3H); **^13^C NMR** (150 MHz, acetone-d_6_):*δ* 151.2, 142.9, 135.7, 132.8, 129.4, 128.8, 125.2, 119.1, 112.5, 112.2, 104.5, 40.8, 21.1; **IR** (cm^−1^) 3378, 2922, 2856, 1704, 1584, 1459, 1362, 1184; **HRMS** (ESI) *m*/*z* calcd for C_24_H_19_N_2_O_2_ (M-(H_2_O) + H)^+^ 367.1441, found 367.1441.
*3,3’-(Hydroxy(m-tolyl)methylene)bis(1H-indol-5-ol)* (**5c**); isolated by column chromatography (EtOAc/petroleum ether = 1:6); (23.4 mg, 61% yield); red amorphous solid; **^1^H NMR** (600 MHz, acetone-d_6_) *δ* 9.68 (s, 2H), 7.52 (s, 2H), 7.23 (d, *J* = 1.8 Hz, 1H), 7.21 (d, *J* = 8.7 Hz, 2H), 7.17–7.13 (m, 2H), 7.01–6.99 (m, 1H), 6.76 (d, *J* = 2.4 Hz, 2H), 6.70–6.69 (m, 3H), 6.68 (d, *J* = 2.4 Hz, 1H), 5.66 (s, 1H), 2.26 (s, 3H); **^13^C NMR** (150 MHz, acetone-d_6_): *δ* 151.2, 145.9, 138.0, 132.8, 130.2, 128.9, 128.7, 127.3, 126.6, 125.2, 119.1, 112.5, 112.2, 104.5, 41.2, 21.6; **HRMS** (ESI) *m*/*z* calcd for C_24_H_19_N_2_O_2_ (M-(H_2_O) + H)^+^ 367.1441, found 367.1442.
*3,3’-((4-ethylphenyl)(hydroxy)methylene)bis(1H-indol-5-ol)* (**5d**); isolated by column chromatography (EtOAc/petroleum ether = 1:5); (21.9 mg, 55% yield); red amorphous solid; **^1^H NMR** (600 MHz, acetone-d_6_) *δ* 9.68 (s, 2H), 7.50 (s, 2H), 7.29–7.27 (m, 2H), 7.21 (d, *J* = 8.4 Hz, 2H), 7.12–7.11(m, 2H), 6.75 (d, *J* = 2.4 Hz, 2H), 6.70 (dd, *J* = 0.9, 2.4 Hz, 2H), 6.68 (d, *J* = 2.4 Hz, 1H), 6.67 (d, *J* = 2.4 Hz, 1H), 5.66 (s, 1H), 2.61 (q, *J* = 7.5 Hz, 2H), 1.20 (t, *J* = 7.8 Hz, 3H); **^13^C NMR** (150 MHz, acetone-d_6_):*δ* 151.2, 143.2, 142.3, 132.9, 129.5, 128.9, 128.2, 125.2, 119.2, 112.5, 112.2, 104.5, 40.9, 16.1; **IR** (cm^−1^) 3305, 2964, 2929, 2869, 1704, 1584, 1458, 1361, 1168; **HRMS** (ESI) *m*/*z* calcd for C_25_H_21_N_2_O_2_ (M-(H_2_O) + H)+ 381.1598, found 381.1597.
*3,3’-((4-(tert-Butyl)phenyl)(hydroxy)methylene)bis(1H-indol-5-ol)* (**5e**); isolated by column chromatography (EtOAc/petroleum ether = 1:6); (15.3 mg, 36% yield); red amorphous solid; **^1^H NMR** (600 MHz, acetone-d_6_) *δ* 9.69(s, 2H), 7.52 (s, 1H), 7.29 (d, *J* = 2.1 Hz, 5H), 7.19 (d, *J* = 8.4 Hz, 3H), 6.75 (d, *J* = 2.4 Hz, 2H), 6.69 (d, *J* = 1.8 Hz, 2H), 6.67 (d, *J* = 2.4 Hz, 1H), 6.65 (d, *J* = 2.4 Hz, 1H), 5.65 (s, 1H), 1.28 (s, 9H); **^13^C NMR** (150 MHz, acetone-d_6_): *δ* 151.3, 149.1, 142.9, 132.8, 129.1, 128.9, 125.6, 125.2, 119.2, 112.5, 112.2, 104.5, 40.7, 34.9, 31.8; **HRMS** (ESI) *m*/*z* calcd for C_27_H_25_N_2_O_2_ (M-(H_2_O) + H)^+^ 409.1911, found 409.1911.
*3,3’-([1,1’-Biphenyl]-4-yl(hydroxy)methylene)bis(1H-indol-5-ol)* (**5f**); isolated by column chromatography (EtOAc/petroleum ether = 1:6); (8.5 mg, 52%) yield); red amorphous solid; **^1^H NMR** (600 MHz, acetone-d_6_) *δ* 9.74 (s, 2H), 7.66–7.65 (m, 2H), 7.58 (d, *J* = 8.4 Hz, 2H), 7.52 (s, 2H), 7.46 (d, *J* = 8.4 Hz, 2H), 7.43 (t, *J* =7.2 Hz, 2H), 7.32 (t, *J* = 7.8 Hz, 1H), 7.23 (d, *J* = 2.4 Hz, 2H), 6.77 (dd, *J* = 1.8, 9.6 Hz, 4H), 6.69 (dd, *J* = 2.4, 8.4 Hz, 2H), 5.76 (s, 1H); **^13^C NMR** (150 MHz, acetone-d_6_): *δ* 151.3, 145.3, 141.8, 139.3, 132.9, 130.1, 129.7, 128.9, 127.9, 127.6, 127.3, 125.3, 118.8, 112.5, 112.3, 104.5, 40.9; **HRMS** (ESI) *m*/*z* calcd for C_29_H_21_N_2_O_2_ (M-(H_2_O) + H)^+^ 429.1598, found 429.1600.
*3,3’-(Hydroxy(naphthalen-2-yl)methylene)bis(1H-indol-5-ol)* (**5g**); isolated by column chromatography (EtOAc/petroleum ether = 1:5); (23.9 mg, 83% yield); red amorphous solid; **^1^H NMR** (600 MHz, acetone-d_6_) *δ* 9.75 (s, 2H), 7.86–7.83 (m, 2H), 7.80 (d, *J* = 8.4 Hz, 1H), 7.77–7.74 (m, 1H), 7.58 (dd, *J* = 1.8, 8.4 Hz, 1H), 7.51 (s, 2H), 7.44–7.41 (m, 2H), 7.23 (d, *J* = 8.4 Hz, 2H), 6.77 (dd, *J* = 2.4, 10.2 Hz, 4H), 6.69 (dd, *J* = 2.4, 8.4 Hz, 2H), 5.89 (s, 1H); **^13^C NMR** (150 MHz, acetone-d_6_): *δ* 151.3, 143.6, 134.6, 133.3, 132.9, 128.9, 128.8, 128.5, 128.4, 128.2, 127.3, 126.6, 126.0, 125.4, 118.7, 112.6, 112.3, 104.5, 41.4; **HRMS** (ESI) *m*/*z* calcd for C_27_H_19_N_2_O_2_ (M-(H_2_O) + H)^+^ 403.1441, found403.1442.
*3,3’-((4-fluorophenyl)(hydroxy)methylene)bis(1H-indol-5-ol)* (**5h**); isolated by column chromatography (EtOAc/petroleum ether = 1:5); (20.6 mg, 70% yield); red amorphous solid; **^1^H NMR** (600 MHz, acetone-d_6_) *δ* 9.73 (s, 2H), 7.57 (s, 2H), 7.40–7.36 (m, 2H), 7.23 (d, *J* = 8.4 Hz, 2H), 7.05–7.01 (m, 2H), 6.75 (d, *J* = 2.4 Hz, 2H), 6.71 (dd, *J* = 8.6, 2.4 Hz, 4H), 5.72 (s, 1H); **^13^C NMR** (150 MHz, acetone-d_6_): *δ* 162.1 (d, *J*_C-F_ = 240 Hz),151.3, 141.9 (d, *J*_C-F_ = 3 Hz), 132.8, 131.1 (d, *J*_C-F_ = 7.5 Hz), 128.7, 125.2, 118.8, 115.3 (d, *J*_C-F_ = 21 Hz), 112.4 (d, *J*_C-F_ = 39 Hz), 104.4, 40.5; **HRMS** (ESI) *m*/*z* calcd for C_23_H_16_FN_2_O_2_ (M-(H_2_O) + H)^+^ 371.1190, found 371.1190.
*3,3’-((3-Fluorophenyl)(hydroxy)methylene)bis(1H-indol-5-ol)* (**5i**); isolated by column chromatography (EtOAc/petroleum ether = 1:5); (20.2 mg, 52% yield); red amorphous solid; **^1^H NMR** (600 MHz, acetone-d_6_) *δ* 9.76 (s, 2H), 7.56 (s, 2H), 7.32–7.29 (m, 1H), 7.23 (d, *J* = 9.0 Hz, 3H), 7.12–7.09 (m, 1H), 6.96–6.93 (m, 1H), 6.76 (s, 4H), 6.70 (dd, *J* = 2.4, 6.0 Hz, 2H), 5.75 (s, 1H); **^13^C NMR** (150 MHz, acetone-d_6_):*δ* 163.7 (d, *J*_C-F_ = 241.5 Hz), 151.4, 149.1 (d, *J*_C-F_ = 6 Hz), 132.8, 130.5 (d, *J*_C-F_ = 7.5 Hz), 128.7, 125.5 (d, *J*_C-F_ = 3 Hz), 125.3, 118.3, 116.1 (d, *J*_C-F_ = 21 Hz), 113.2 (d, *J*_C-F_ = 21 Hz), 112.5 (d, *J*_C-F_ = 36 Hz), 104.3, 41.0; **HRMS** (ESI) *m*/*z* calcd for C_23_H_16_FN_2_O_2_ (M-(H_2_O) + H)^+^ 371.1190, found 371.1191.
*3,3’-((3-Chlorophenyl)(hydroxy)methylene)bis(1H-indol-5-ol)* (**5j**); isolated by column chromatography (EtOAc/petroleum ether = 1:3); (24.6 mg, 61% yield); red amorphous solid; **^1^H NMR** (600 MHz, acetone-d_6_) *δ* 9.81 (s, 2H), 7.60 (d, *J* = 9.0 Hz, 2H), 7.38 (s, 1H), 7.34–7.28 (m, 2H), 7.22 (d, *J* = 8.4 Hz, 4H), 6.73 (s, 4H), 6.69–6.67 (m, 2H), 5.72 (s, 1H); **^13^C NMR** (150 MHz, acetone-d_6_):*δ* 151.4, 148.6, 134.2, 132.8, 130.5, 129.4, 128.6, 128.1, 126.7, 125.3, 118.2, 112.6, 112.4, 104.3, 40.9; **IR** (cm^−1^) 3304, 2921, 2851, 1697, 1585, 1463, 1423, 1363, 1187; **HRMS** (ESI) *m*/*z* calcd for C_23_H_16_ClN_2_O_2_ (M-(H_2_O) + H)^+^ 387.0895, found 387.0894.
*3,3’-((4-Chlorophenyl)(hydroxy)methylene)bis(1H-indol-5-ol)* (**5k**); isolated by column chromatography (EtOAc/petroleum ether = 1:5); (23.8 mg, 59% yield); red amorphous solid; **^1^H NMR** (600 MHz, acetone-d_6_) *δ* 9.75 (s, 2H), 7.55 (s, 2H), 7.38–7.35 (m, 2H), 7.31– 7.28 (m, 2H), 7.23 (d, *J* = 6.0 Hz, 1H), 6.75–6.71 (m, 4H), 6.70 (dd, *J* = 2.4, 9.0 Hz, 2H), 5.71 (s, 1H); **^13^C NMR** (150 MHz, acetone-d_6_):*δ* 151.3, 144.9, 132.8, 131.8, 131.2, 128.8, 128.7, 125.3, 118.4, 112.6, 112.4, 104.4, 40.6; **HRMS** (ESI) *m*/*z* calcd for C_23_H_16_ClN_2_O_2_ (M-(H_2_O) + H)^+^ 387.0895, found 387.0895.
*3,3’-(Hydroxy(3-methoxyphenyl)methylene)bis(1H-indol-5-ol)* (**5l**); isolated by column chromatography (EtOAc/petroleum ether = 1:5); (24.4 mg, 61% yield); red amorphous solid; **^1^H NMR** (600 MHz, acetone-d_6_) *δ* 9.70 (s, 2H), 7.53 (s, 2H), 7.17–7.22 (m, 3H), 6.96–6.98 (m, 2H), 6.77 (d, *J* = 6.0 Hz, 2H), 6.74 (d, *J* = 6.0 Hz, 2H), 6.68 (dd, *J* = 8.4 Hz, 2.4 Hz, 2H), 5.68 (s, 1H), 3.71 (s, 3H); **^13^C NMR** (150 MHz, acetone-d_6_): *δ* 160.5, 151.1, 147.5, 132.7, 128.7, 125.1, 121.8, 118.7, 115.6, 112.4, 111.4, 104.4, 55.1, 41.2; **HRMS** (ESI) *m*/*z* calcd for C_24_H_21_N_2_O_4_ (M + H)^+^ 401.14958, found 401.14975.
*1-(4-(Hydroxybis(5-hydroxy-1H-indol-3-yl)methyl)phenyl)pentan-1-one* (**5m**); isolated by column chromatography (EtOAc/petroleum ether = 1:5); (24.5 mg, 55% yield); red amorphous solid; **^1^H NMR** (600 MHz, acetone-d_6_) *δ* 9.76 (s, 2H), 7.92 (d, *J* = 8.4 Hz, 2H), 7.55 (s, 2H), 7.49 (d, *J* = 8.4 Hz, 2H), 7.23 (d, *J* = 8.4 Hz, 2H), 6.75 (d, *J* = 1.8 Hz, 4H), 6.70 (dd, *J* = 2.4, 8.4 Hz, 2H), 5.79 (s, 1H), 2.99 (t, *J* = 7.2 Hz, 2H), 1.69–1.64 (m, 2H), 1.42–1.36 (m, 2H), 0.92 (t, *J* = 7.2 Hz, 3H); **^13^C NMR** (150 MHz, acetone-d_6_):*δ* 200.1, 151.42, 151.37, 136.1, 132.9, 128.8, 125.4, 118.2, 112.7, 112.4, 104.3, 41.4, 38.6, 27.3, 23.1, 14.3; **HRMS** (ESI) *m*/*z* calcd for C_28_H_25_N_2_O_3_ (M-(H_2_O) + H)^+^ 437.1865, found 437.1868.
*3,3’-(Hydroxy(thiophen-2-yl)methylene)bis(1H-indol-5-ol)* (**5n**); isolated by column chromatography (EtOAc/petroleum ether = 1:3); (15.0 mg, 40% yield); red amorphous solid; **^1^H NMR** (600 MHz, acetone-d_6_) *δ* 9.74 (s, 2H), 7.56 (s, 2H), 7.24–7.22 (m, 3H), 6.92–6.91 (m, 4H), 6.86 (d, *J* = 2.4 Hz, 2H), 6.72(dd, *J* = 2.4, 6.0 Hz, 2H), 6.01 (s, 1H); **^13^C NMR** (150 MHz, acetone-d_6_): *δ* 151.3, 150.6, 132.7, 128.5, 127.0, 125.5, 124.8, 124.1, 119.0, 112.6, 112.1, 104.4, 36.3; **HRMS** (ESI) *m*/*z* calcd for C_21_H_15_N_2_O_2_S (M-(H_2_O) + H)^+^ 359.0854, found 359.0852.
