# Supplementary material for: Friedel–Crafts Reaction of Acylsilanes: Highly Chemoselective Synthesis of 1-Hydroxy-bis(indolyl)methanes and 1-Silyl-bis(indolyl)methanes Derivatives
Source: Molecules. 2023 Jul 27;28(15):5685. doi: 10.3390/molecules28155685 (PMC10420641; doi:10.3390/molecules28155685)
Supplement: Supplementary file 1 [file molecules-28-05685-s001.zip › molecules-2431858-supplementary.pdf]

Supporting Information

Friedel-Crafts Reaction of Acylsilanes: Highly Chemoselective Synthesis of 1-Hydroxy-bis(indolyl)methanes and 1-Silyl-bis(indolyl)methanes Derivatives

Qi Li,[†] Xiu-Xia Liang,[†] Wang Zhang,[†] and Man-Yi Han*[†]

[†]Key Laboratory of Green and Precise Synthetic Chemistry and Applications, Ministry of Education; College of Chemistry and Materials Science, Huaibei Normal University, Huaibei, Anhui 235000, P.R. China

E-Mail: hanmy10@126.com; hanmy@chnu.edu.cn

Table of Contents

1. The reaction of acylsilane with indole containing hydroxy groups at different positions………………………..……..……..……...S2
2. ^{1}H and ^{13}C NMR spectra of the products…………………………S3

1. The reaction of acylsilane with indole containing hydroxy groups at different positions

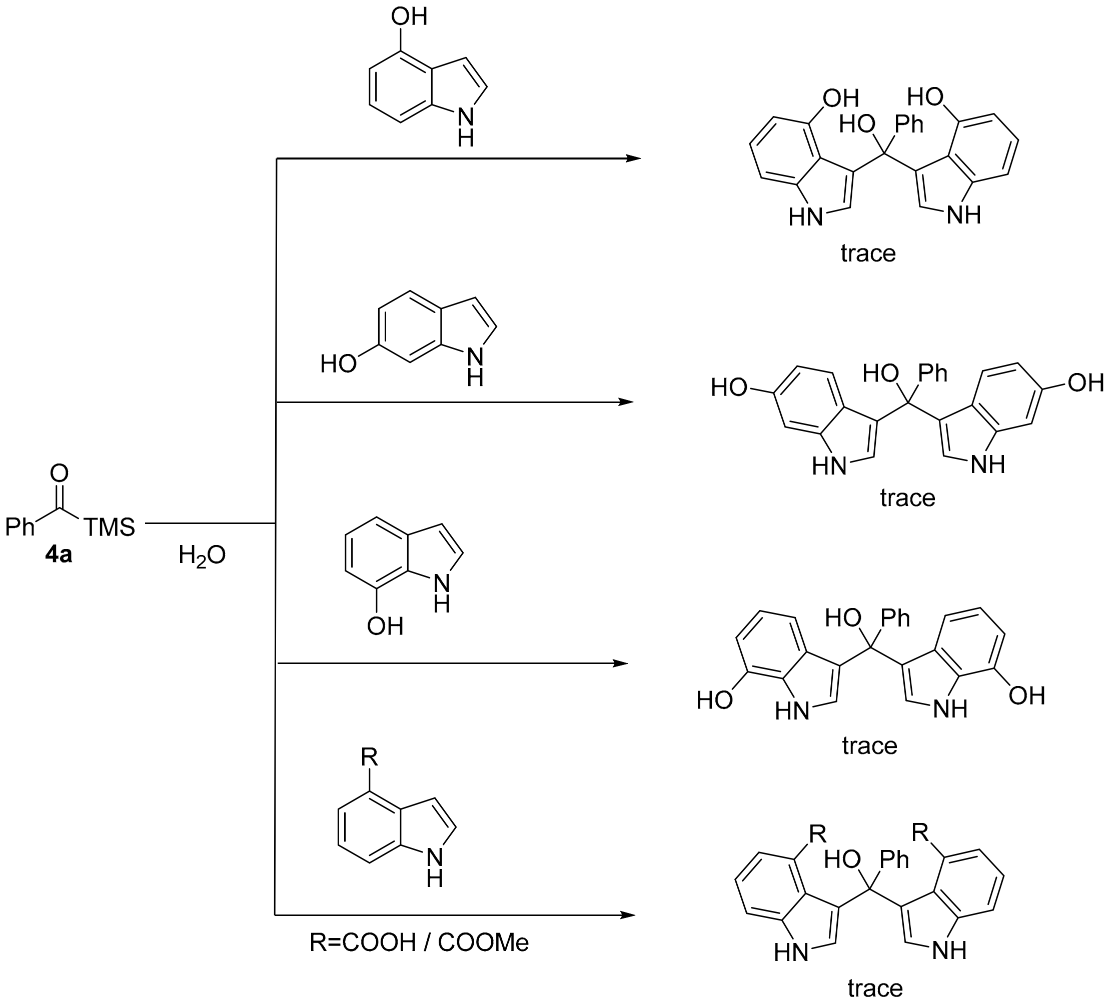

2.^{1}H and ^{13}C NMR spectraof the products

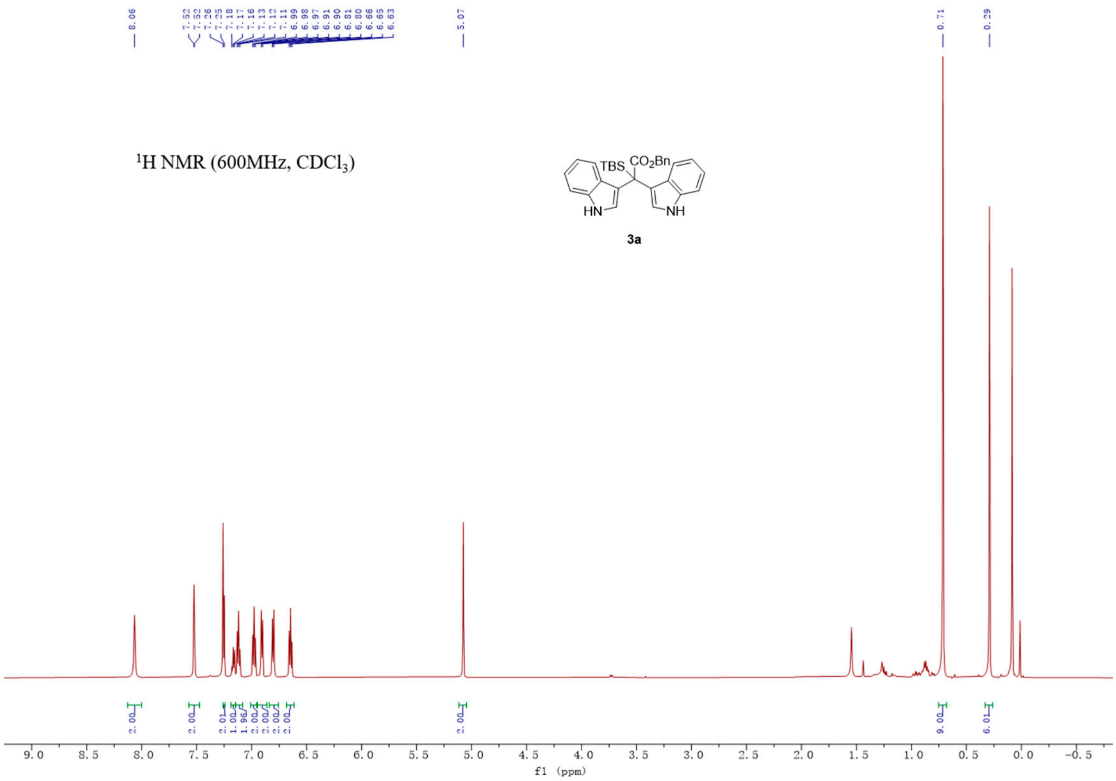

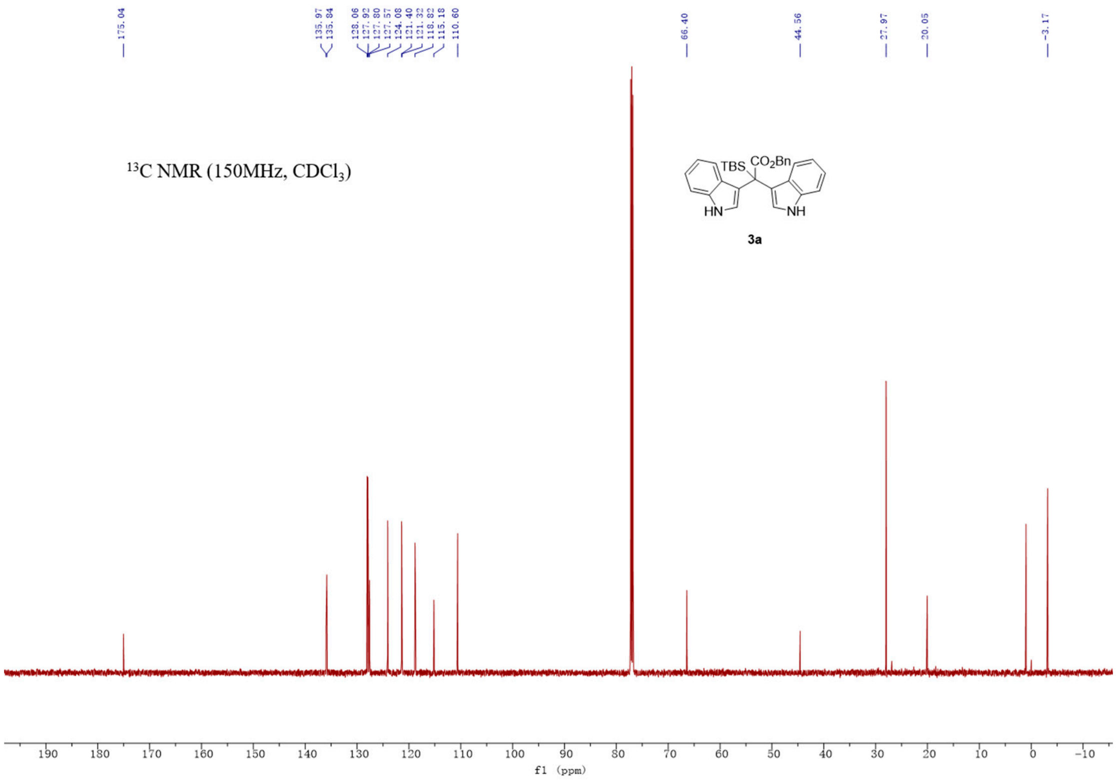

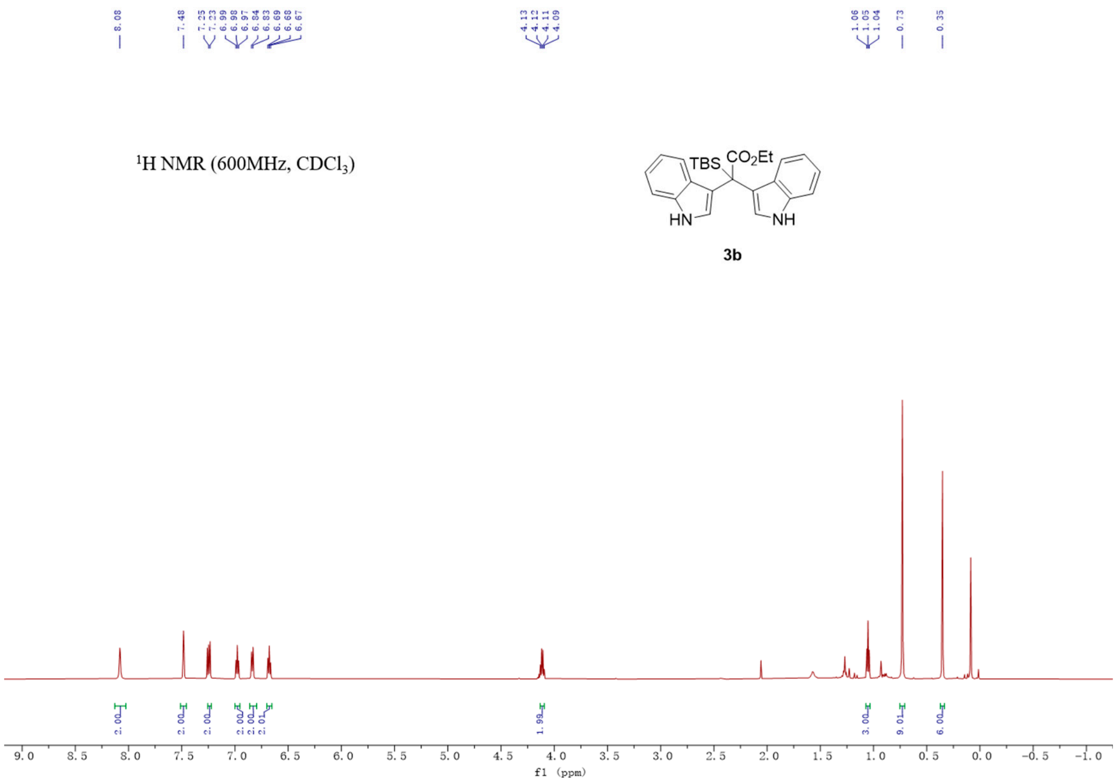
8.08
7.48
7.25
7.23
6.99
6.98
6.97
6.84
6.83
6.69
6.68
6.67
4.13
4.12
4.11
4.09
1.06
1.05
1.04
0.73
0.35
^{1}H NMR (600MHz, CDCl$_3$)
TBS
CO_2Et
HN
NH
3b
2.00
2.00
2.00
2.00
2.00
2.01
1.98
3.00
9.01
6.00
f1 (ppm)

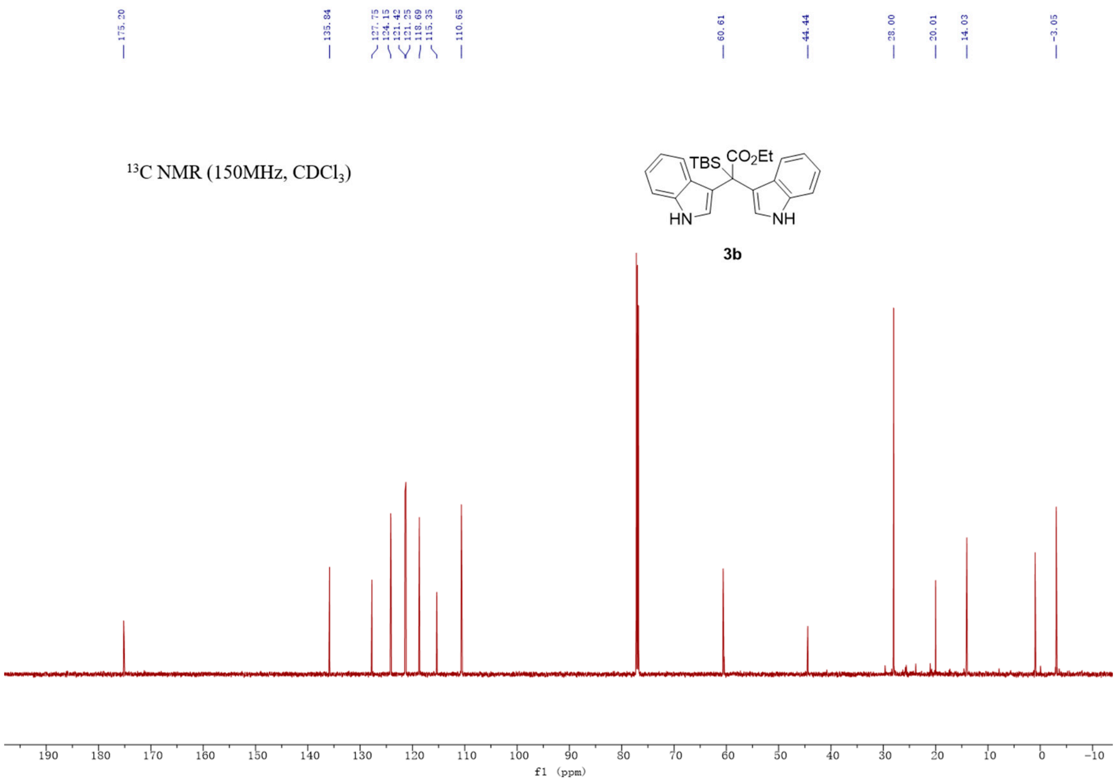
175.20
135.84
127.75
124.15
121.42
121.25
118.69
115.35
110.65
60.61
44.44
28.00
20.01
14.03
-3.05
^{13}C NMR (150MHz, CDCl$_3$)
TBS
CO_2Et
HN
NH
3b
f1 (ppm)

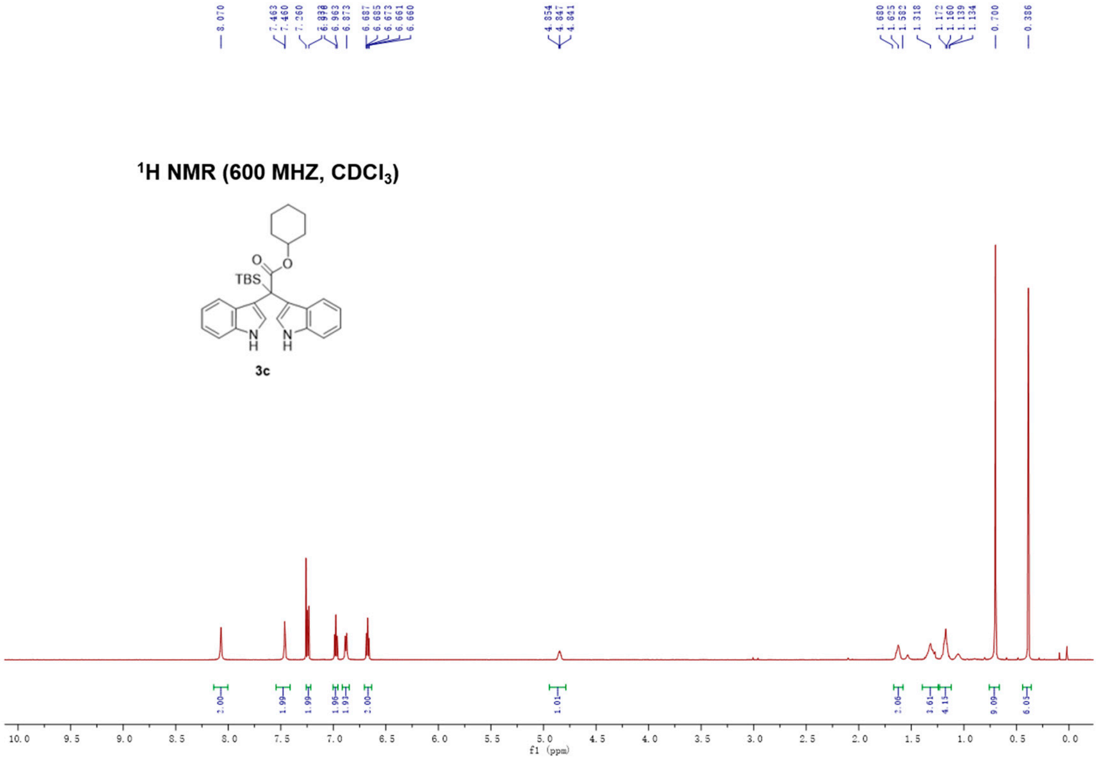

¹H NMR (600 MHZ, CDCl₃)
TBS
O
N H
3c
8.070
7.463
7.460
7.260
6.963
6.873
6.687
6.685
6.673
6.661
6.660
4.854
4.847
4.841
1.680
1.625
1.582
1.318
1.172
1.160
1.139
1.134
0.700
0.386
f1 (ppm)

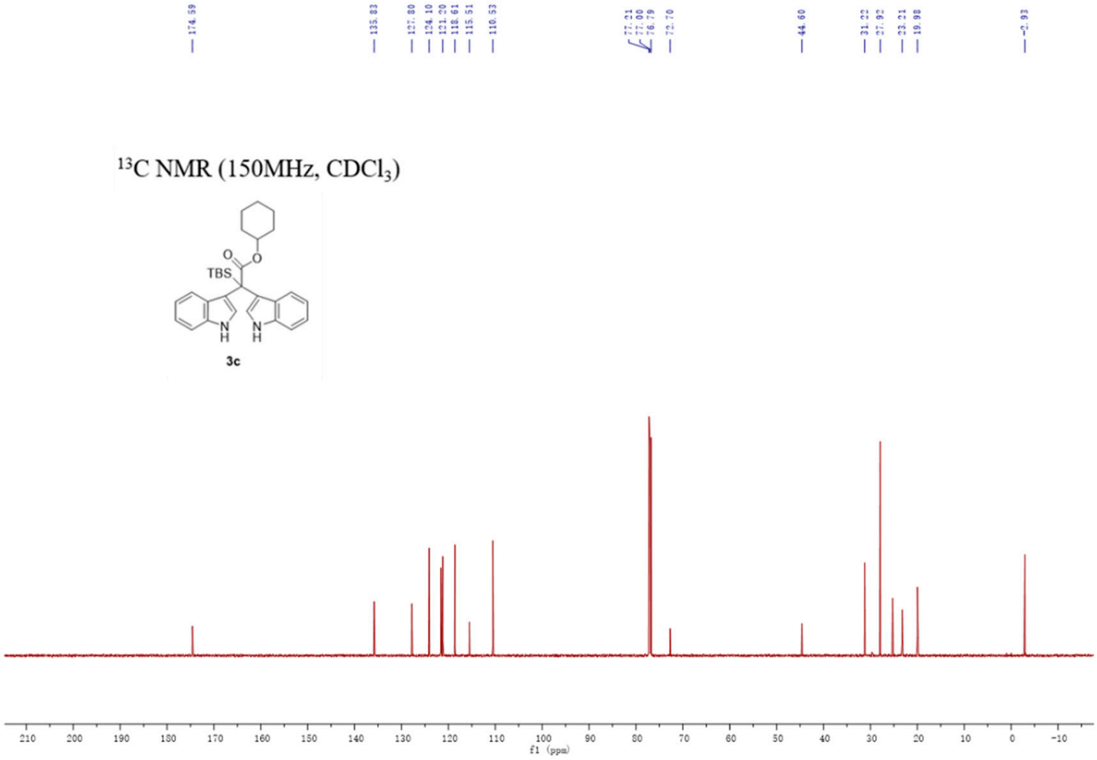

¹³C NMR (150MHz, CDCl₃)
TBS
O
N H
3c
174.59
135.83
127.80
124.10
121.20
118.61
115.51
110.53
77.21
77.00
76.79
72.70
44.60
31.22
27.92
23.21
19.98
-2.93
f1 (ppm)

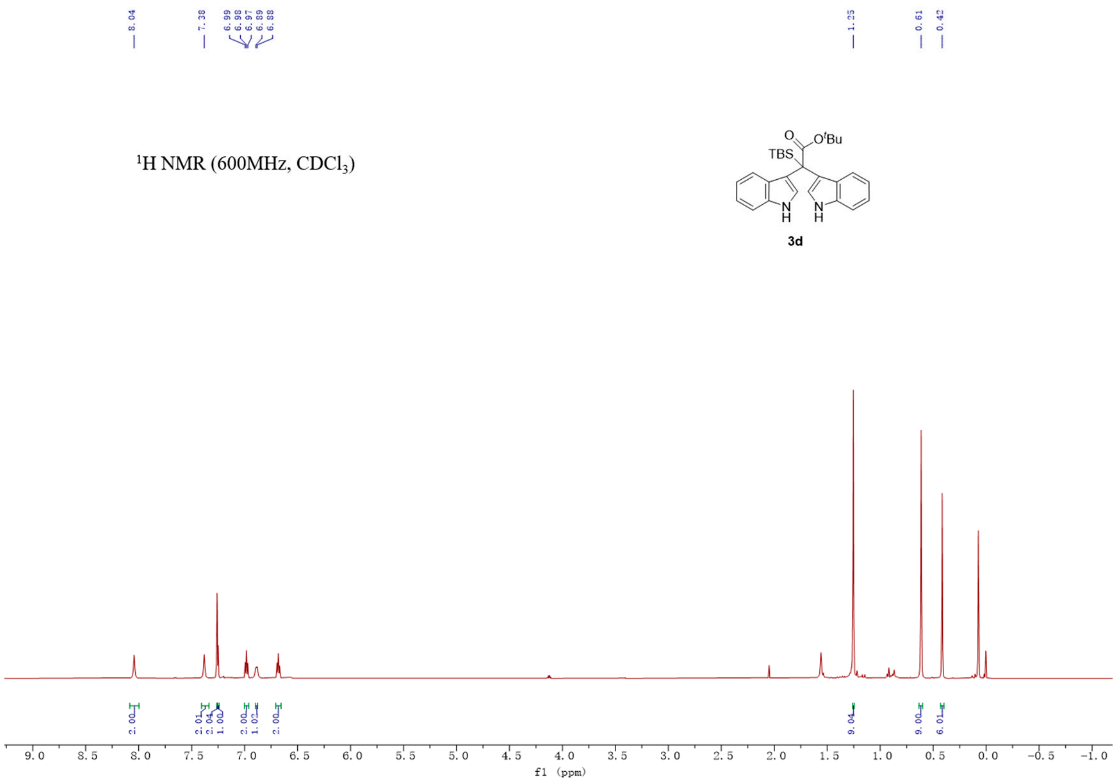

1H NMR (600MHz, CDCl3)
O
O'Bu
TBS
N
H
N
H
3d
8.04
7.38
6.99
6.98
6.97
6.89
6.88
1.25
0.61
0.42
2.00
2.01
2.04
1.00
2.00
1.02
2.00
9.04
9.00
6.01
f1 (ppm)

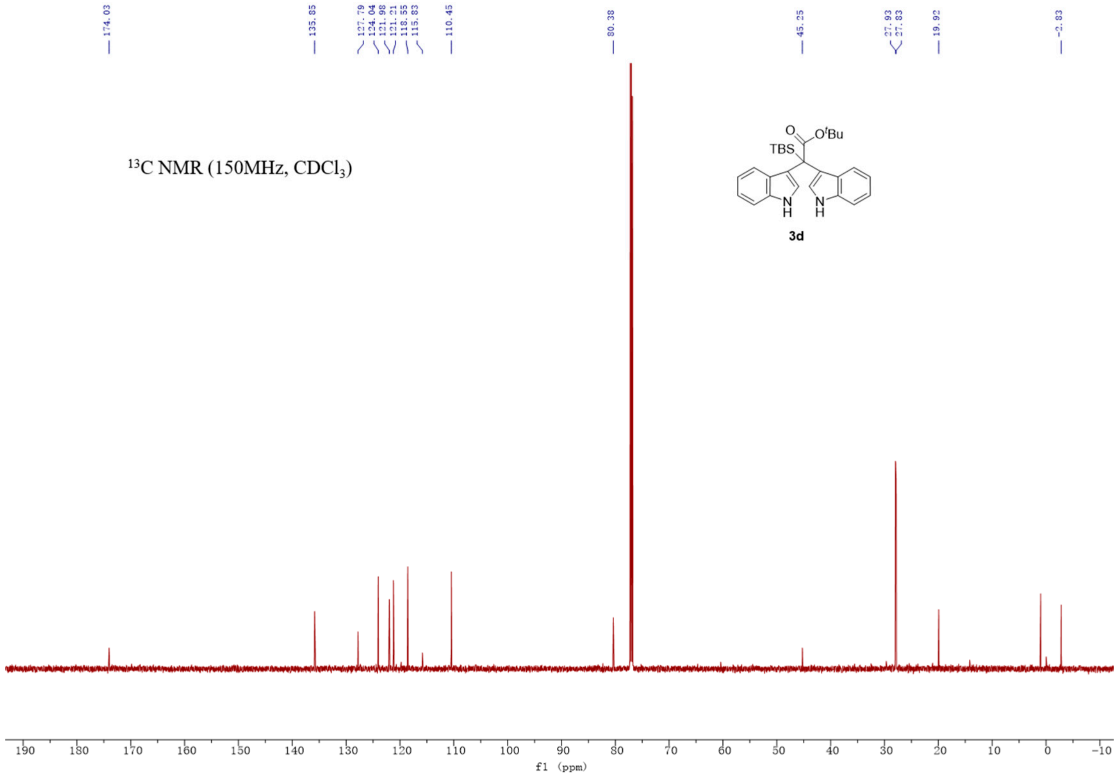

13C NMR (150MHz, CDCl3)
O
O'Bu
TBS
N
H
N
H
3d
174.03
135.85
127.79
124.04
121.98
121.21
118.55
115.83
110.45
80.38
45.25
27.93
27.83
19.92
-2.83
f1 (ppm)

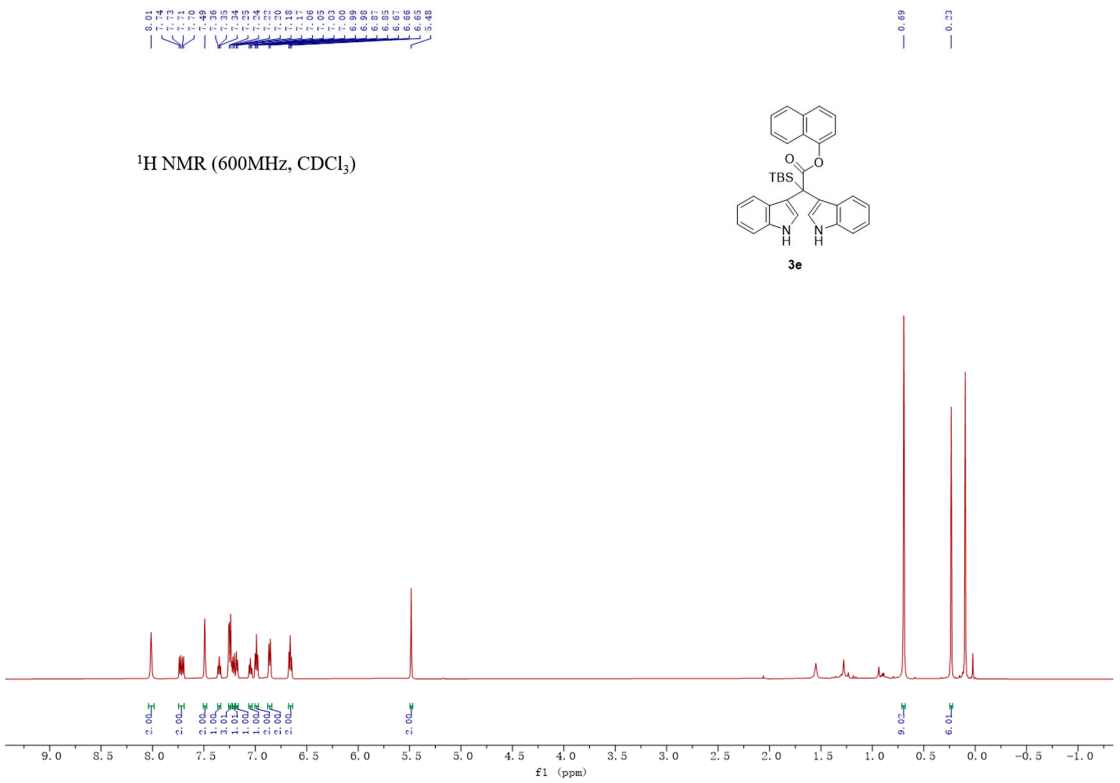
8.01 7.74 7.73 7.71 7.70 7.49 7.36 7.35 7.34 7.25 7.24 7.22 7.20 7.18 7.17 7.06 7.05 7.03 7.00 6.99 6.98 6.87 6.85 6.67 6.66 6.65 5.48
0.69
0.23
^{1}H NMR (600MHz, CDCl$_3$)
O
O
TBS
N
H
N
H
3e
2.00 2.00 2.00 1.00 3.01 1.01 1.00 1.00 2.00 2.00 2.00
2.00
9.02
6.01
9.0 8.5 8.0 7.5 7.0 6.5 6.0 5.5 5.0 4.5 4.0 3.5 3.0 2.5 2.0 1.5 1.0 0.5 0.0 −0.5 −1.0
f1 (ppm)

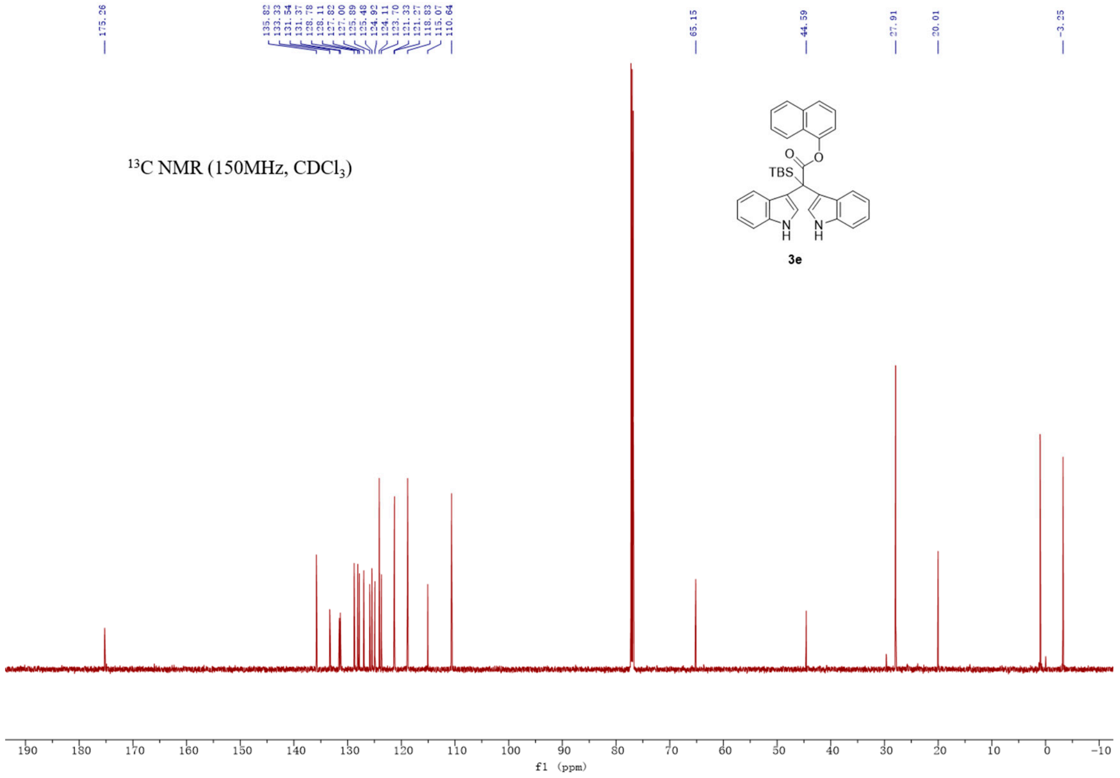
175.26
135.82 133.33 131.54 131.37 128.78 128.11 127.82 127.00 125.89 125.48 124.92 124.11 123.70 121.33 121.27 118.83 115.07 110.64
65.15
44.59
27.91
20.01
−3.25
^{13}C NMR (150MHz, CDCl$_3$)
O
O
TBS
N
H
N
H
3e
190 180 170 160 150 140 130 120 110 100 90 80 70 60 50 40 30 20 10 0 −10
f1 (ppm)

^1H NMR (600MHz, $CDCl_3$)

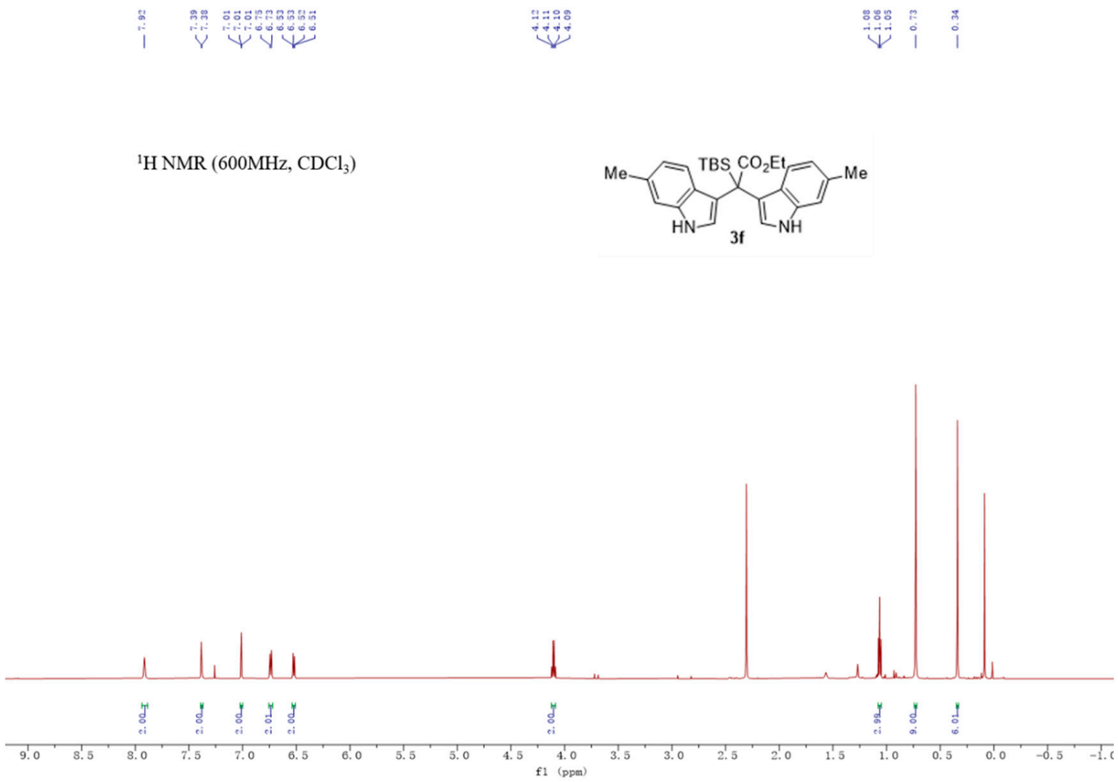

^{13}C NMR (150MHz, $CDCl_3$)

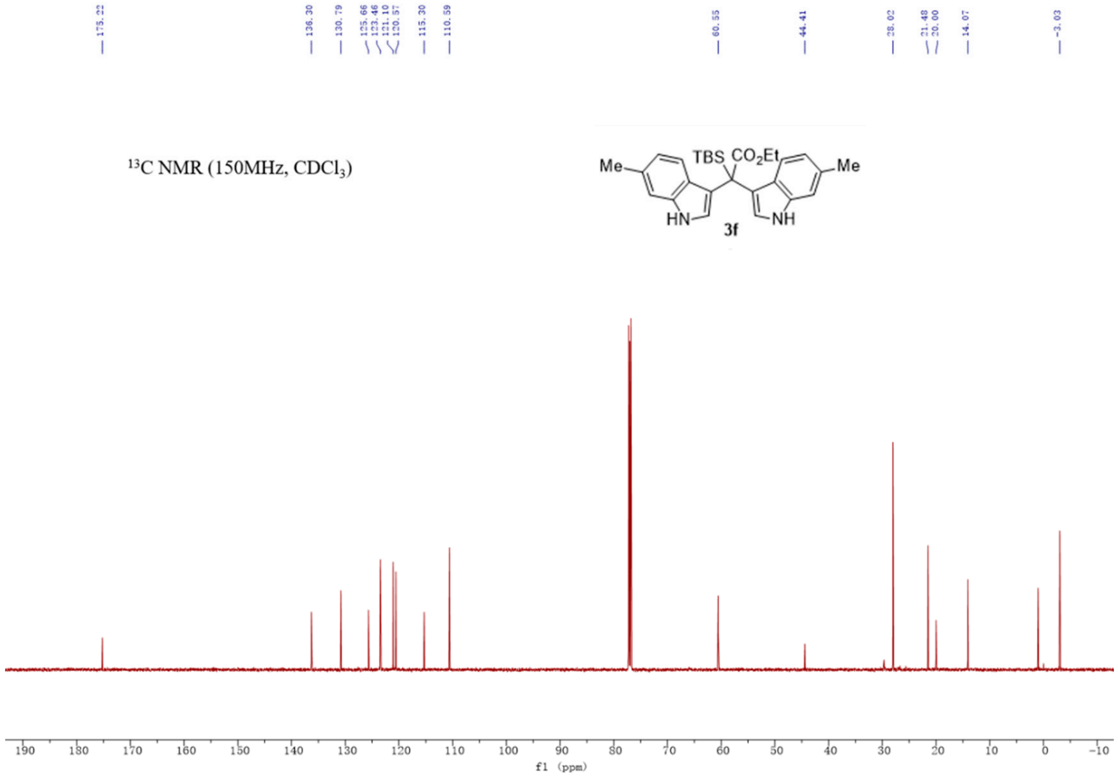

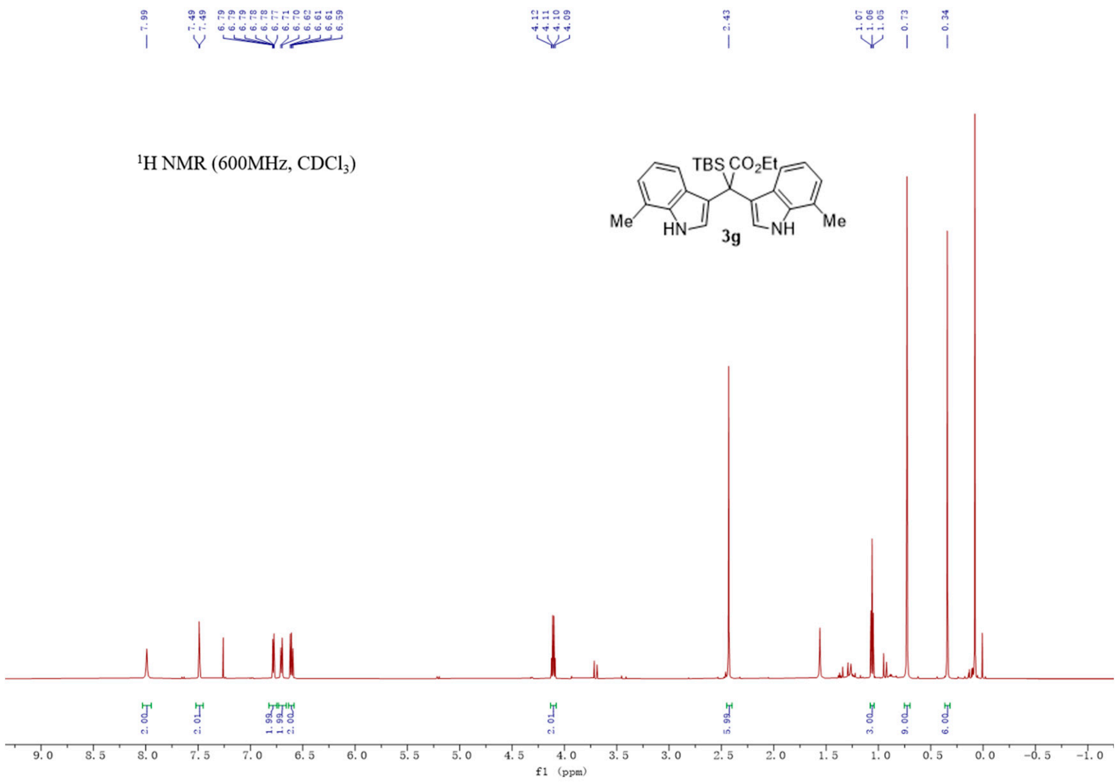

1H NMR (600MHz, CDCl3)
TBS CO2Et
Me
HN
3g
NH
Me
f1 (ppm)

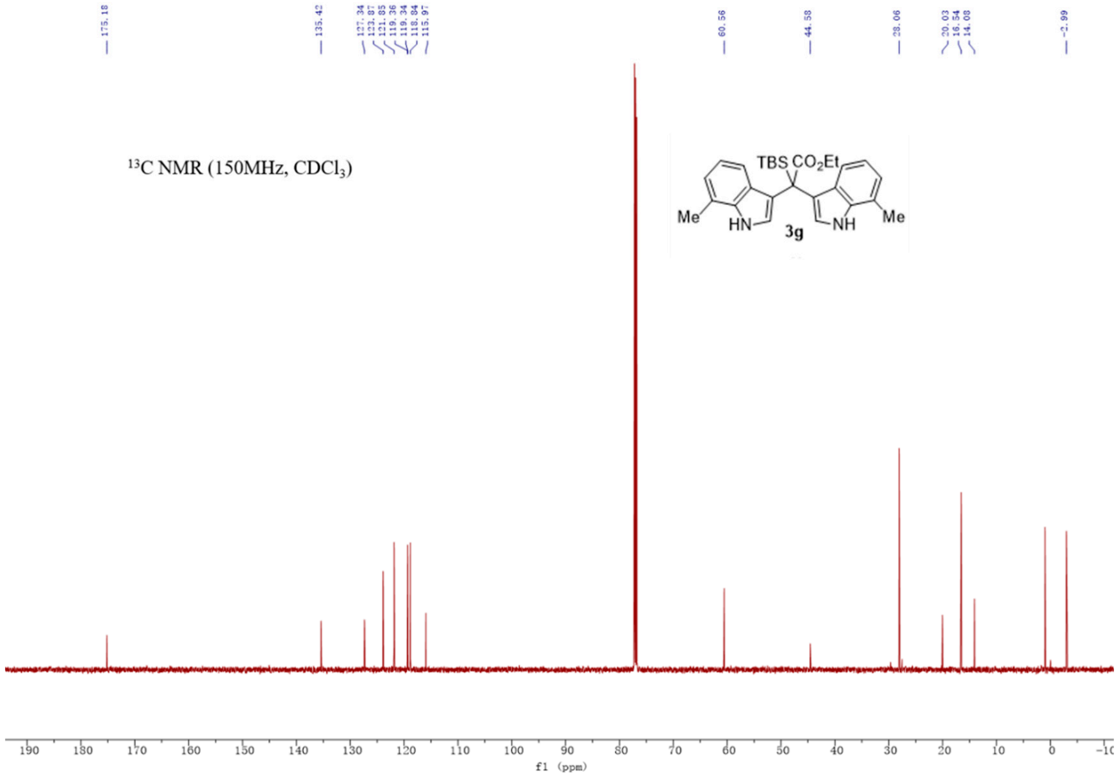

13C NMR (150MHz, CDCl3)
TBS CO2Et
Me
HN
3g
NH
Me
f1 (ppm)

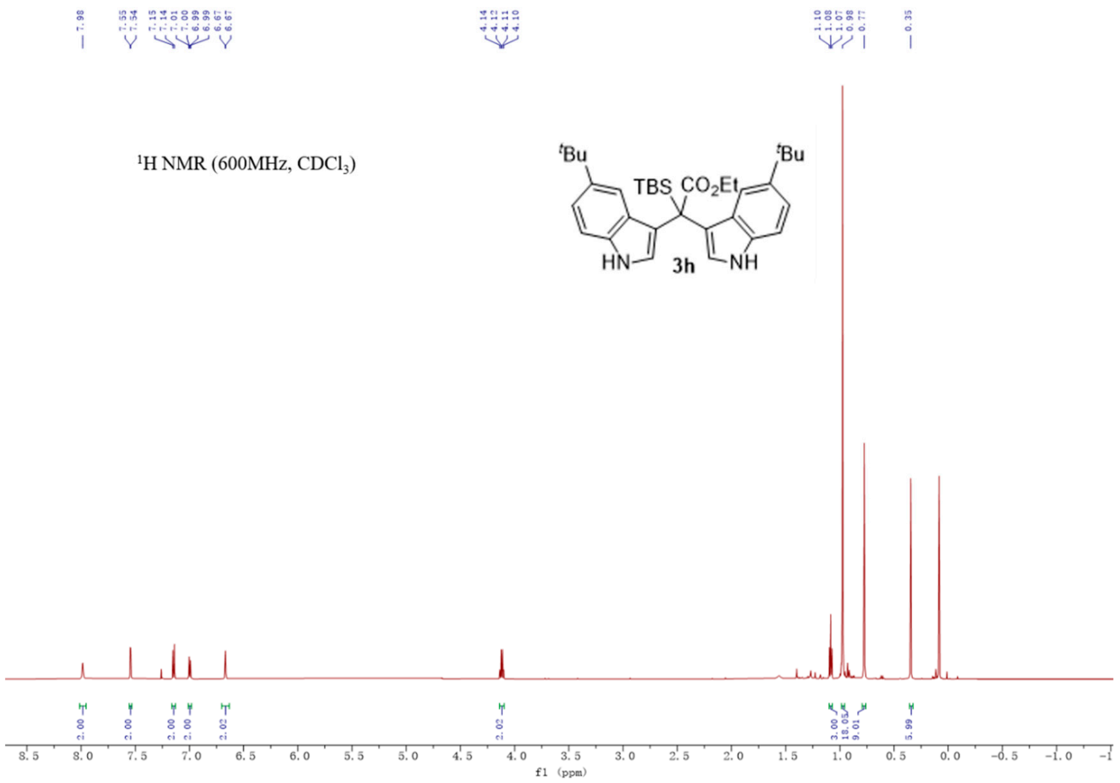

^1H NMR (600MHz, $CDCl_3$)

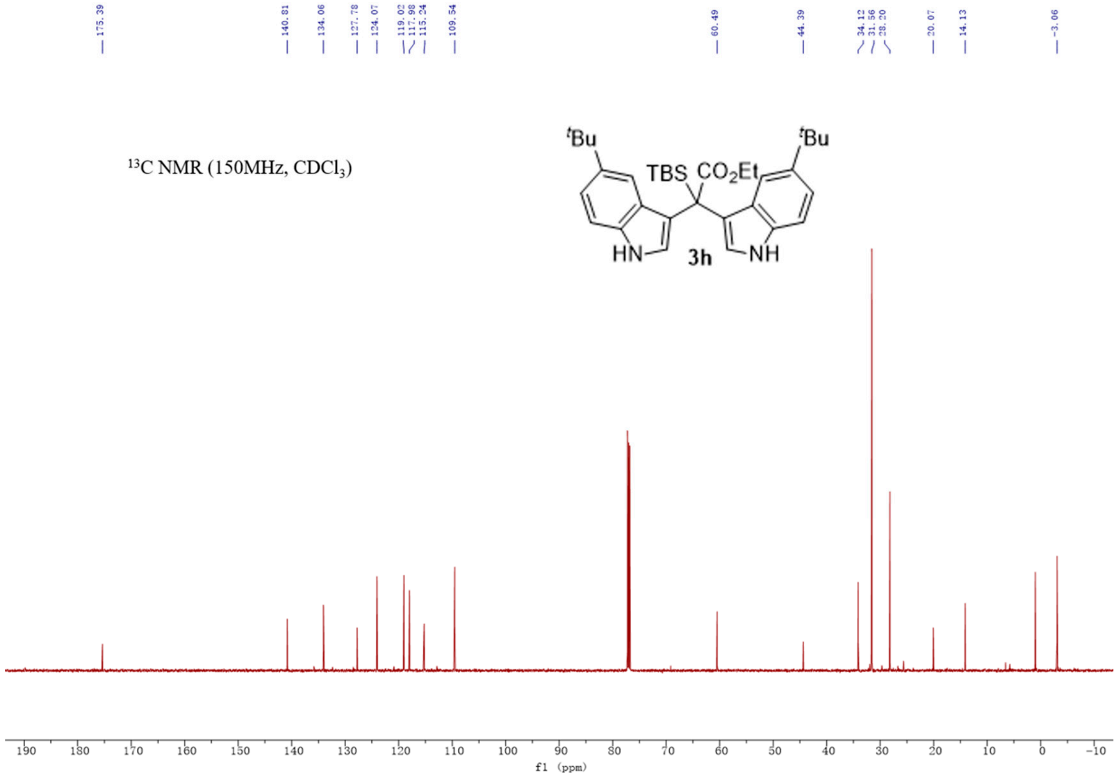

^{13}C NMR (150MHz, $CDCl_3$)

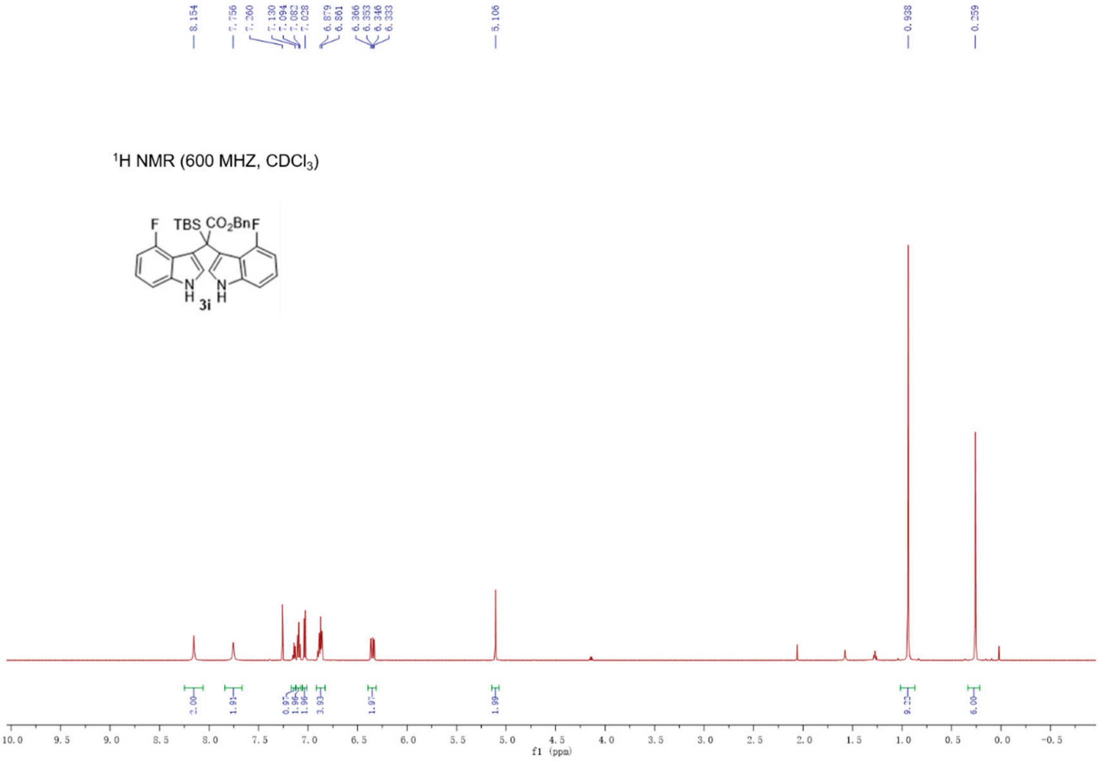
8.154
7.756
7.260
7.130
7.094
7.082
7.028
6.879
6.861
6.366
6.353
6.346
6.333
5.106
0.938
0.259
1H NMR (600 MHZ, CDCl3)
F TBS CO2BnF
N H 3i H N
2.00
1.91
0.97
1.96
1.96
3.93
1.97
1.99
9.23
6.00
f1 (ppm)

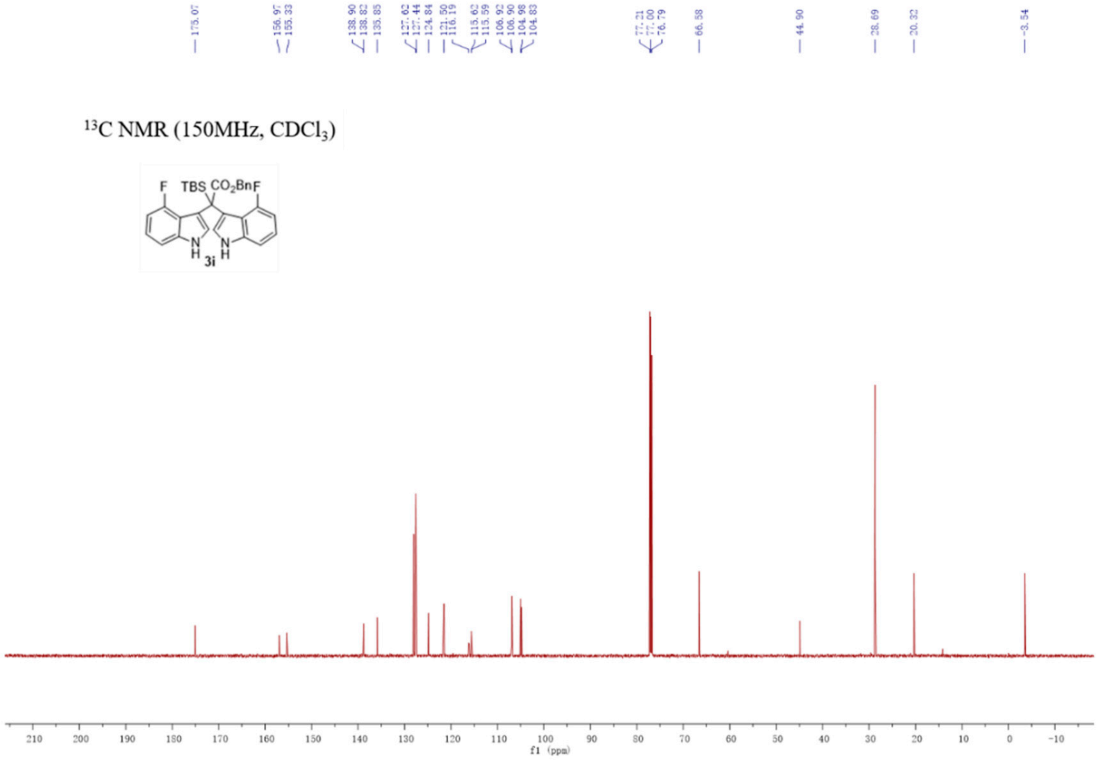
175.07
156.97
155.33
138.90
138.82
135.85
127.62
127.44
124.84
121.50
116.19
115.62
115.59
106.92
106.90
104.98
104.83
77.21
77.00
76.79
66.58
44.90
28.69
20.32
-3.54
13C NMR (150MHz, CDCl3)
F TBS CO2BnF
N H 3i H N
f1 (ppm)

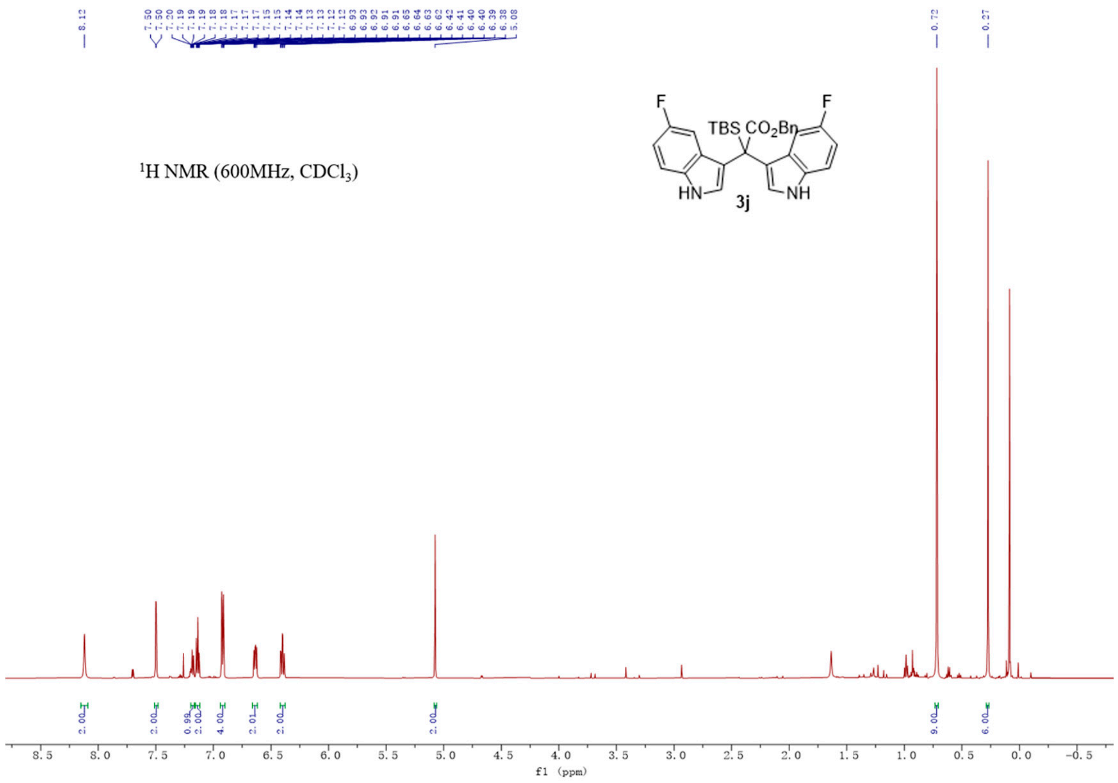

1H NMR (600MHz, CDCl3)
3j

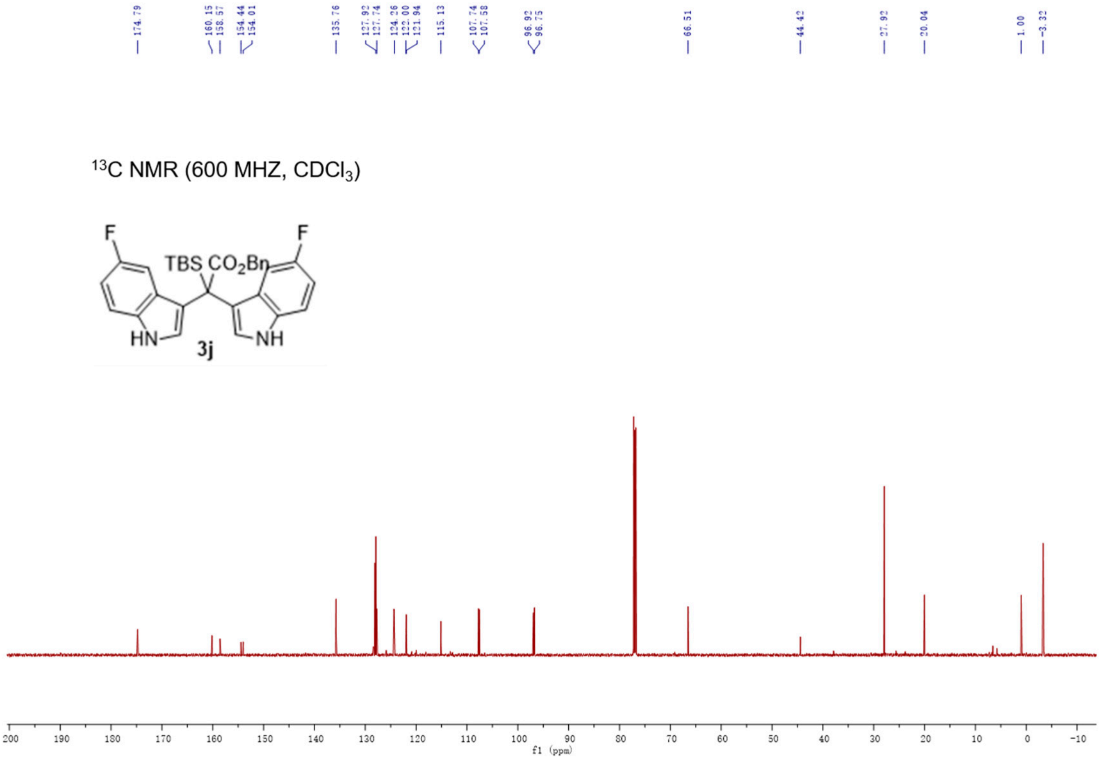

13C NMR (600 MHZ, CDCl3)
3j

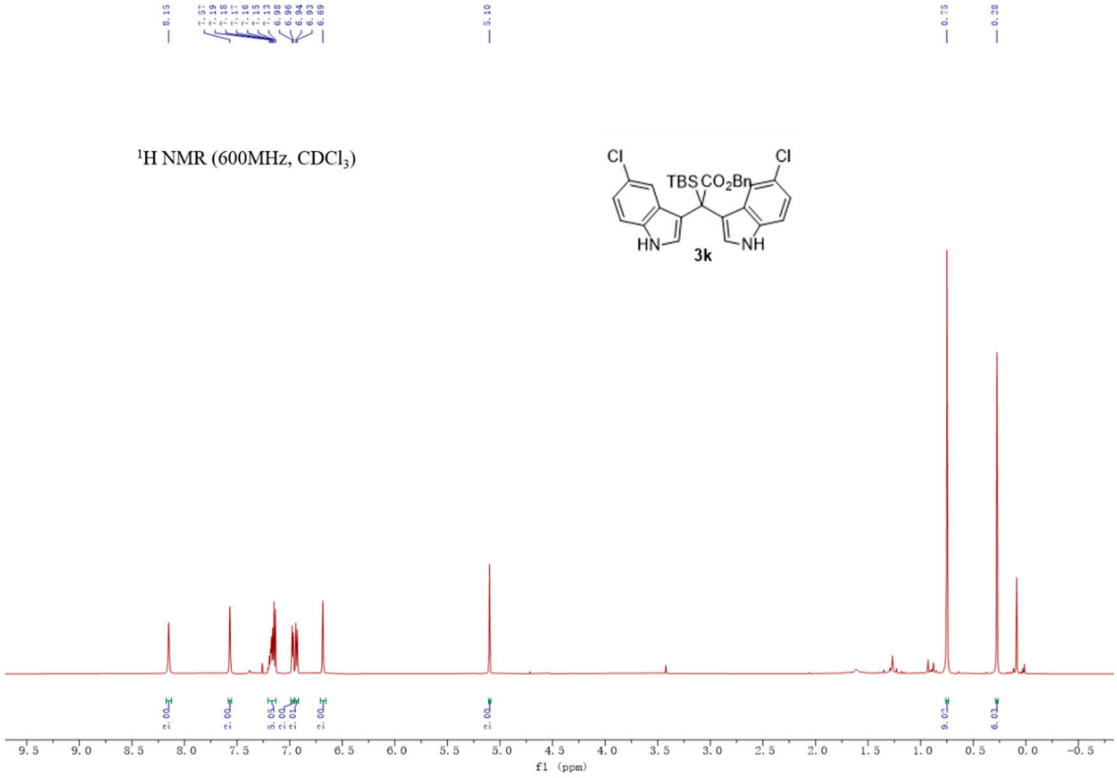

8.15
7.57
7.19
7.18
7.17
7.16
7.15
7.13
6.98
6.96
6.94
6.93
6.69
5.10
0.75
0.28
^{1}H NMR (600MHz, $CDCl_3$)
Cl
TBS
CO_2Bn
Cl
HN
NH
3k
2.00
2.00
5.05
2.00
2.01
2.00
2.00
9.02
6.01
f1 (ppm)

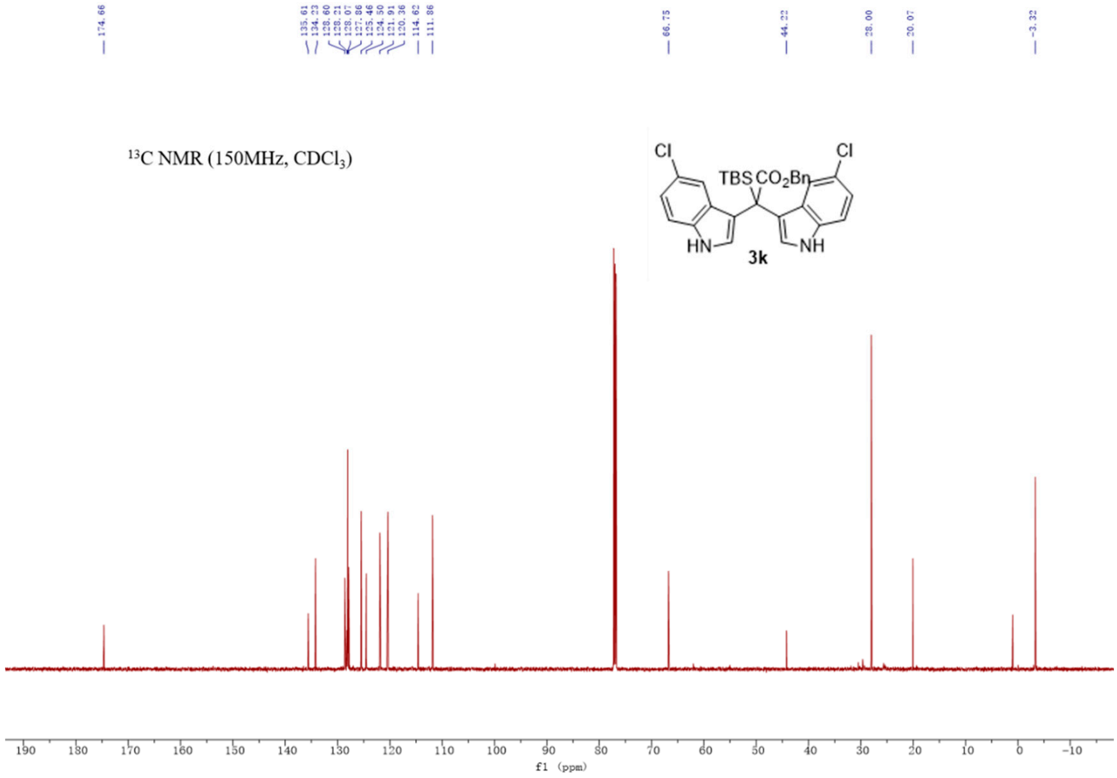

174.66
135.61
134.23
128.60
128.21
128.07
127.86
125.46
124.50
121.91
120.36
114.62
111.86
66.75
44.22
28.00
20.07
-3.32
^{13}C NMR (150MHz, $CDCl_3$)
Cl
TBS
CO_2Bn
Cl
HN
NH
3k
f1 (ppm)

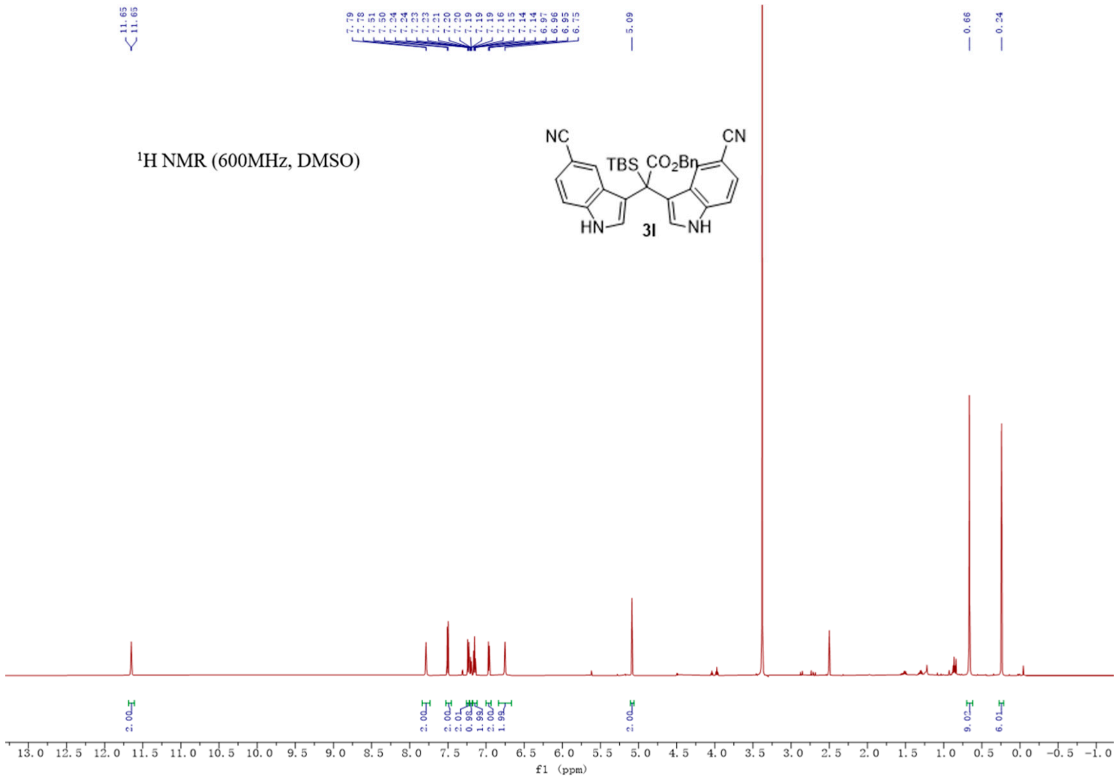

^{1}H NMR (600MHz, DMSO)
NC
CN
TBS
CO_2Bn
HN
NH
3l
f1 (ppm)

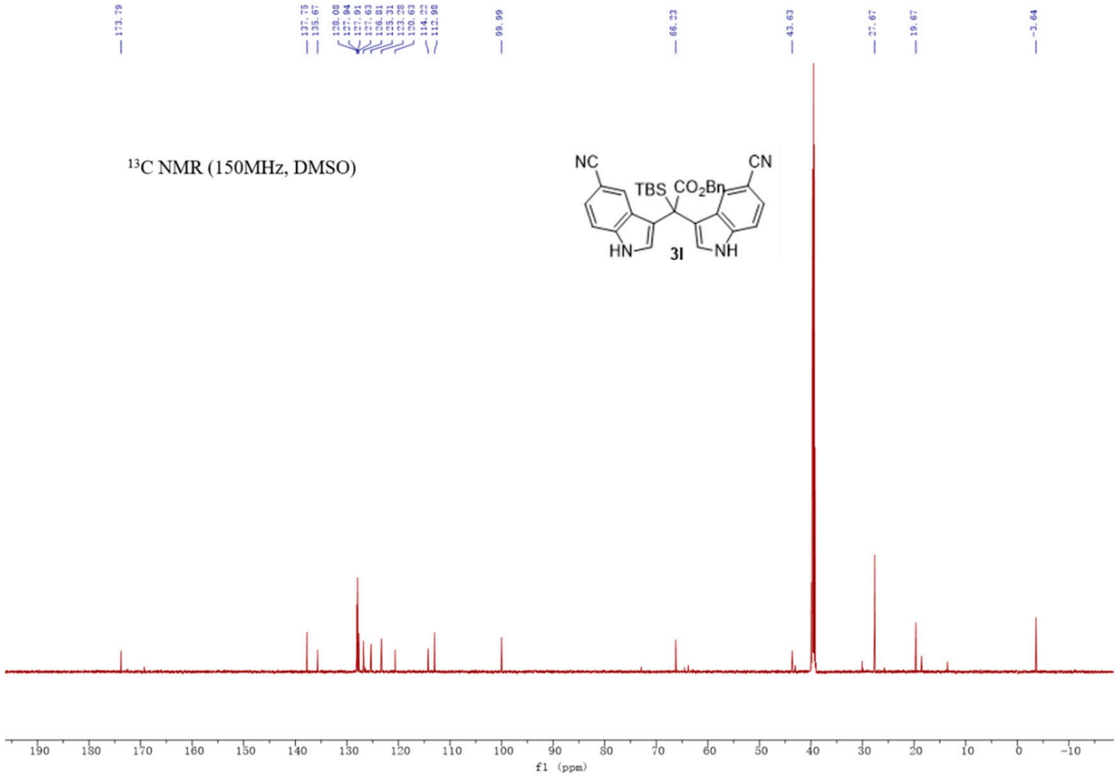

^{13}C NMR (150MHz, DMSO)
NC
CN
TBS
CO_2Bn
HN
NH
3l
f1 (ppm)

^{1}H NMR (600MHz, $CDCl_3$)

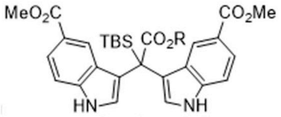

3m, R = o-BrBn

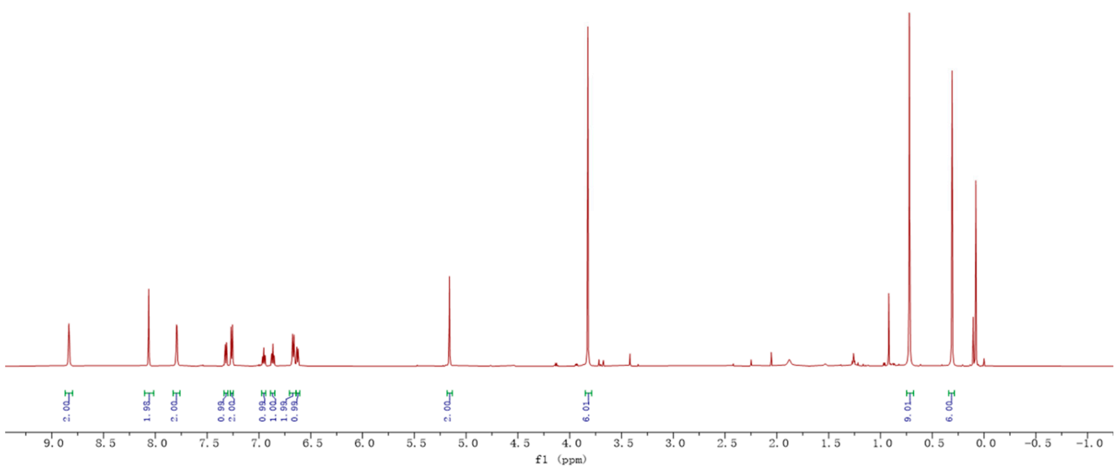

^{13}C NMR (150MHz, $CDCl_3$)

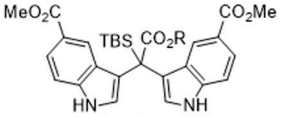

3m, R = o-BrBn

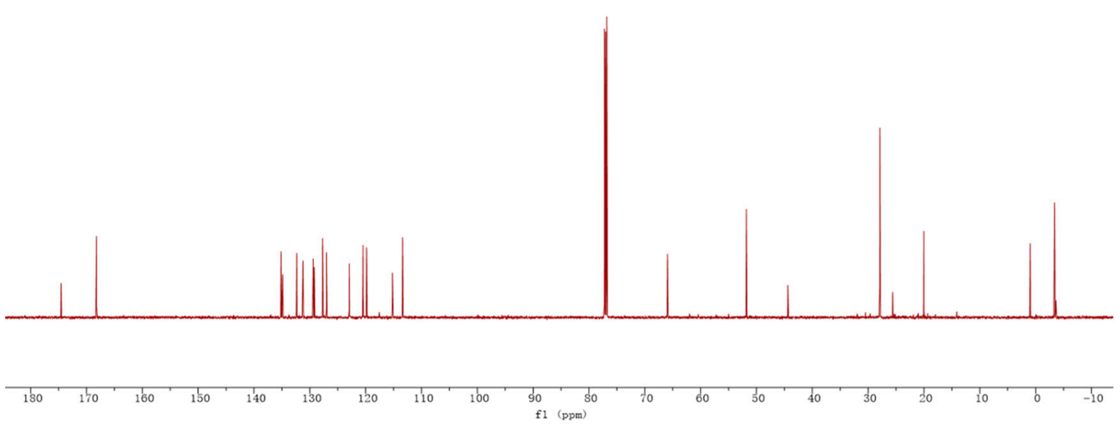

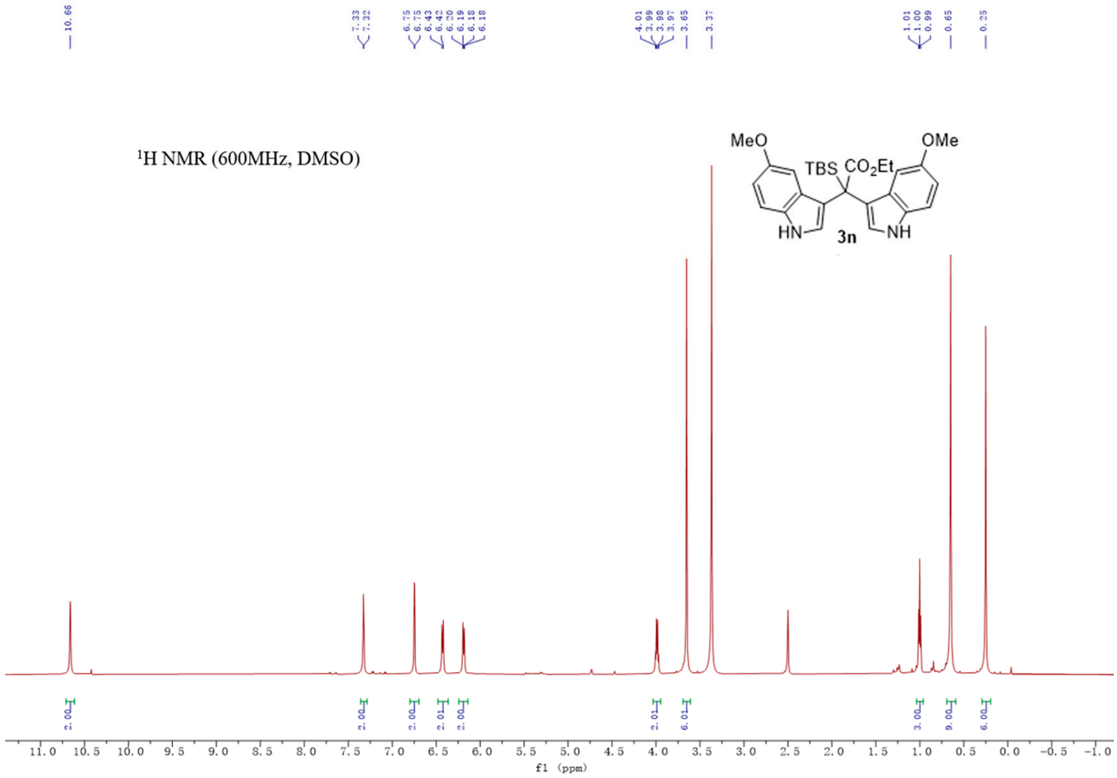

10.66
7.33
7.32
6.75
6.75
6.43
6.42
6.20
6.19
6.18
6.18
4.01
3.99
3.98
3.97
3.65
3.37
1.01
1.00
0.99
0.65
0.25
¹H NMR (600MHz, DMSO)
MeO
TBS
CO2Et
OMe
HN
3n
NH
2.00
2.00
2.00
2.01
2.00
2.01
6.01
3.00
9.00
6.00
11.0 10.5 10.0 9.5 9.0 8.5 8.0 7.5 7.0 6.5 6.0 5.5 5.0 4.5 4.0 3.5 3.0 2.5 2.0 1.5 1.0 0.5 0.0 -0.5 -1.0
f1 (ppm)

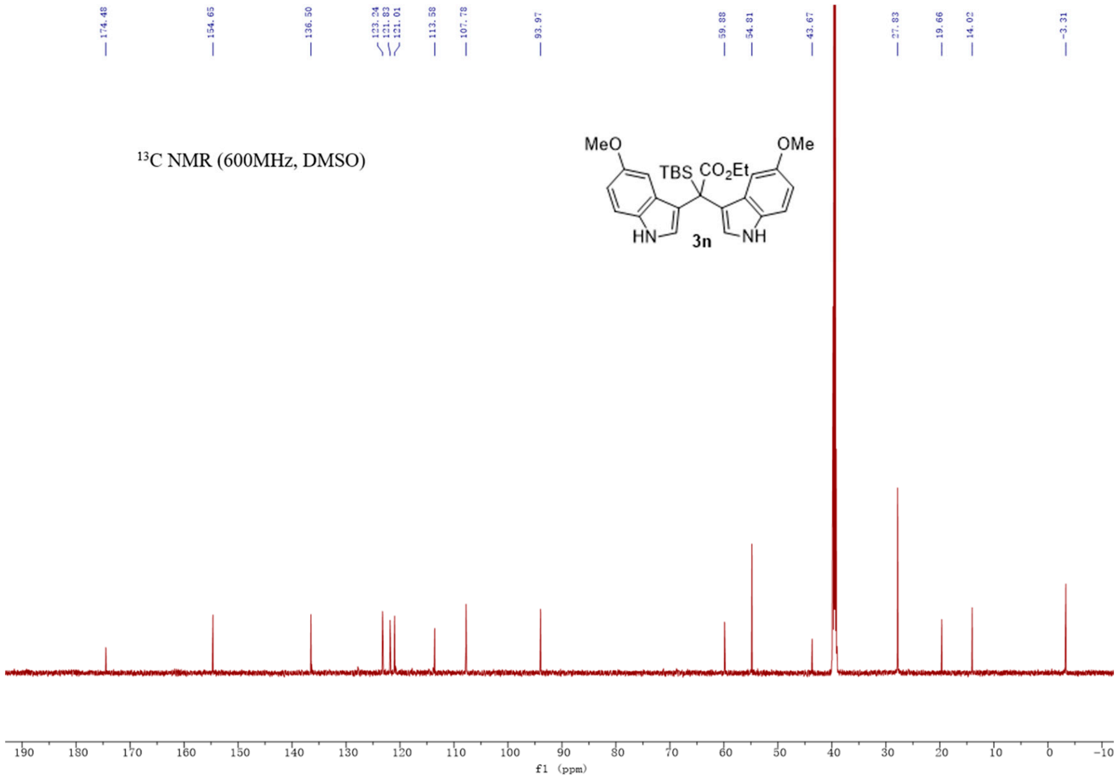

174.48
154.65
136.50
123.24
121.83
121.01
113.58
107.78
93.97
59.88
54.81
43.67
27.83
19.66
14.02
-3.31
¹³C NMR (600MHz, DMSO)
MeO
TBS
CO2Et
OMe
HN
3n
NH
190 180 170 160 150 140 130 120 110 100 90 80 70 60 50 40 30 20 10 0 -10
f1 (ppm)

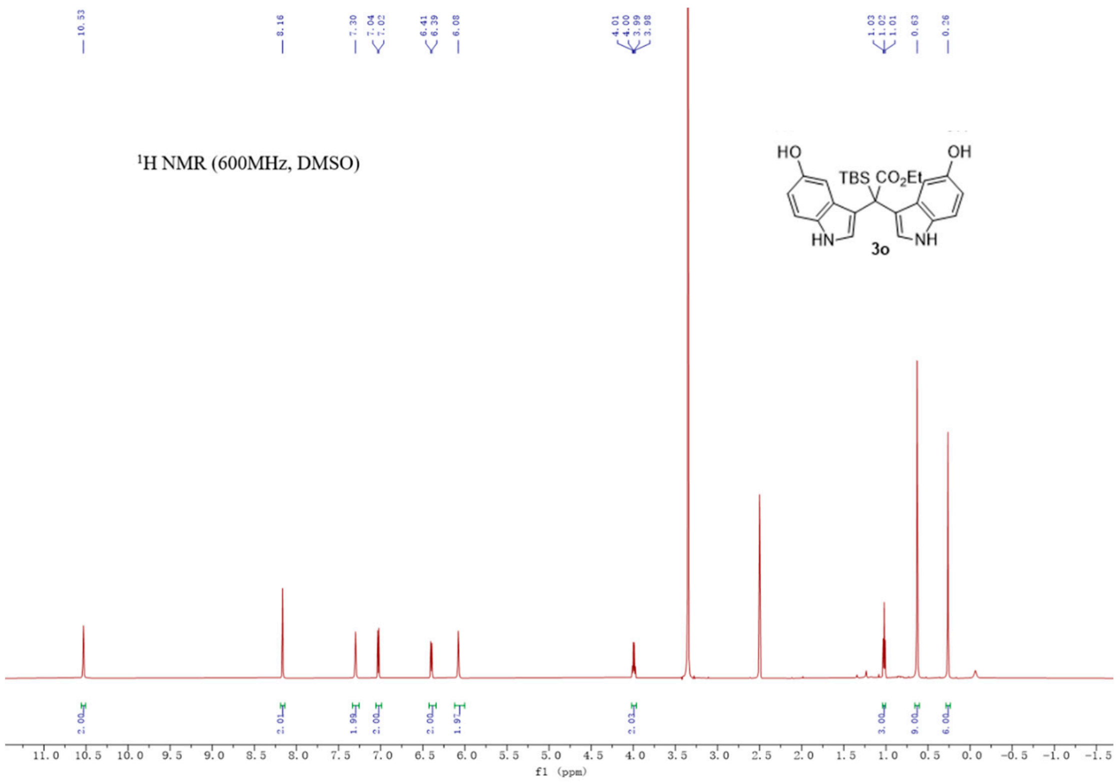

10.53
8.16
7.30
7.04
7.02
6.41
6.39
6.08
4.01
4.00
3.99
3.98
1.03
1.02
1.01
0.63
0.26
1H NMR (600MHz, DMSO)
HO
OH
TBS
CO2Et
HN
NH
3o
2.00
2.01
1.99
2.00
2.00
1.97
2.01
3.00
9.00
6.00
f1 (ppm)

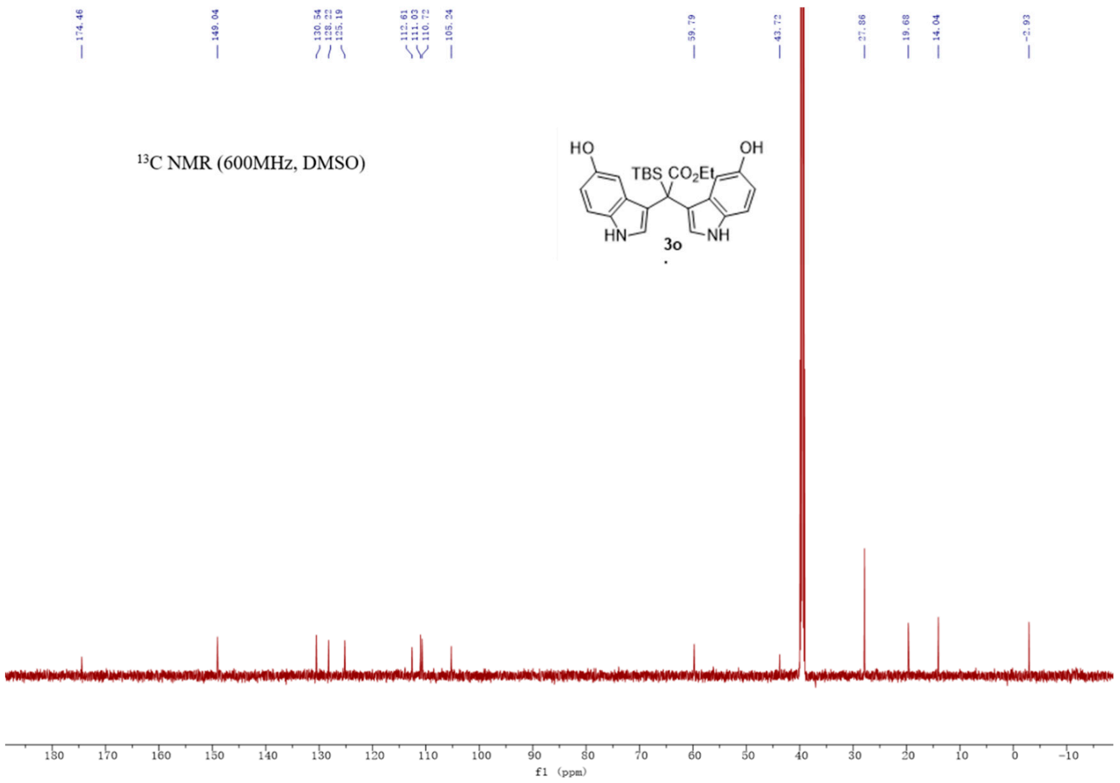

174.46
149.04
130.54
128.22
125.19
112.61
111.03
110.72
105.24
59.79
43.72
27.86
19.68
14.04
-2.93
13C NMR (600MHz, DMSO)
HO
OH
TBS
CO2Et
HN
NH
3o
f1 (ppm)

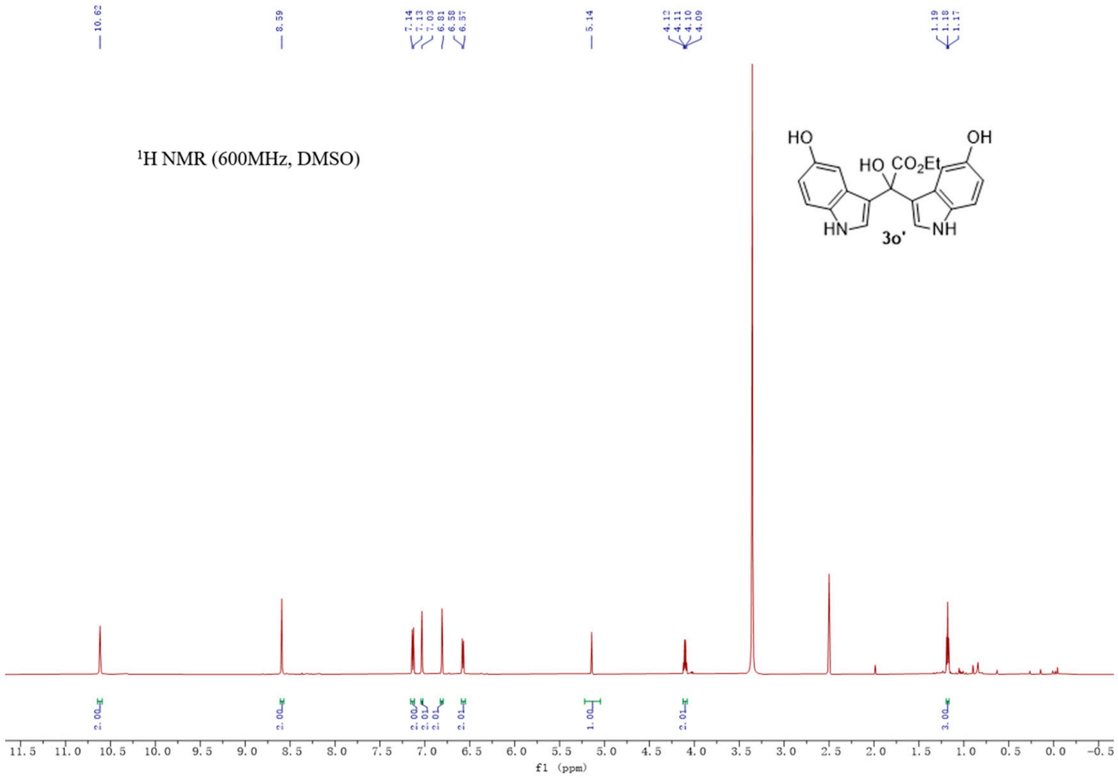
10.62
8.59
7.14
7.13
7.03
6.81
6.58
6.57
5.14
4.12
4.11
4.10
4.09
1.19
1.18
1.17
1H NMR (600MHz, DMSO)
HO
OH
HO CO2Et
HN
NH
3o'
2.00
2.00
2.00
2.01
2.01
2.01
1.00
2.01
3.00
f1 (ppm)

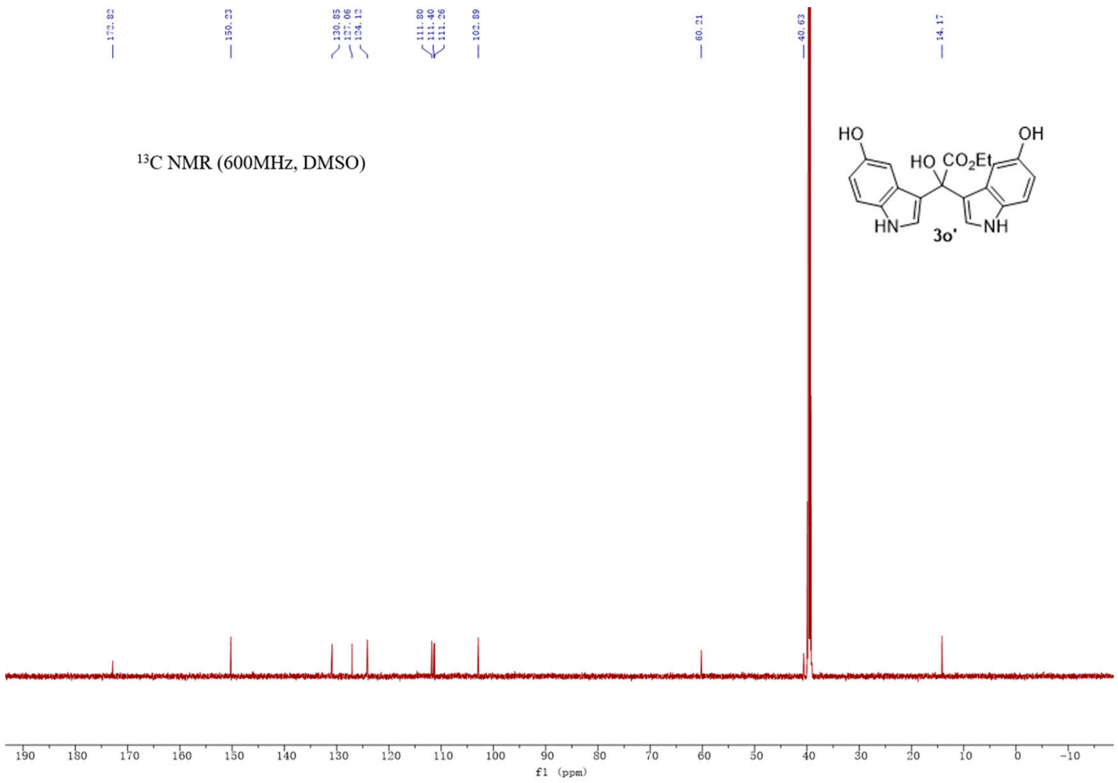
172.82
150.23
130.85
127.06
124.12
111.80
111.40
111.26
102.89
60.21
40.63
14.17
13C NMR (600MHz, DMSO)
HO
OH
HO CO2Et
HN
NH
3o'
f1 (ppm)

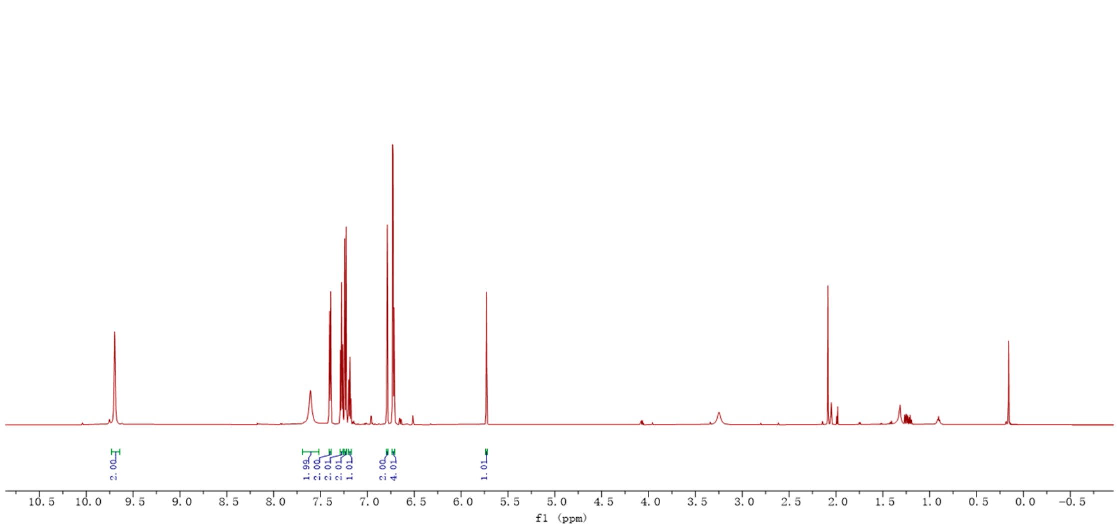

^{1}H NMR (600MHz, Acetone)

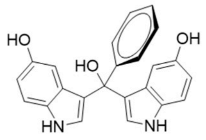

5a

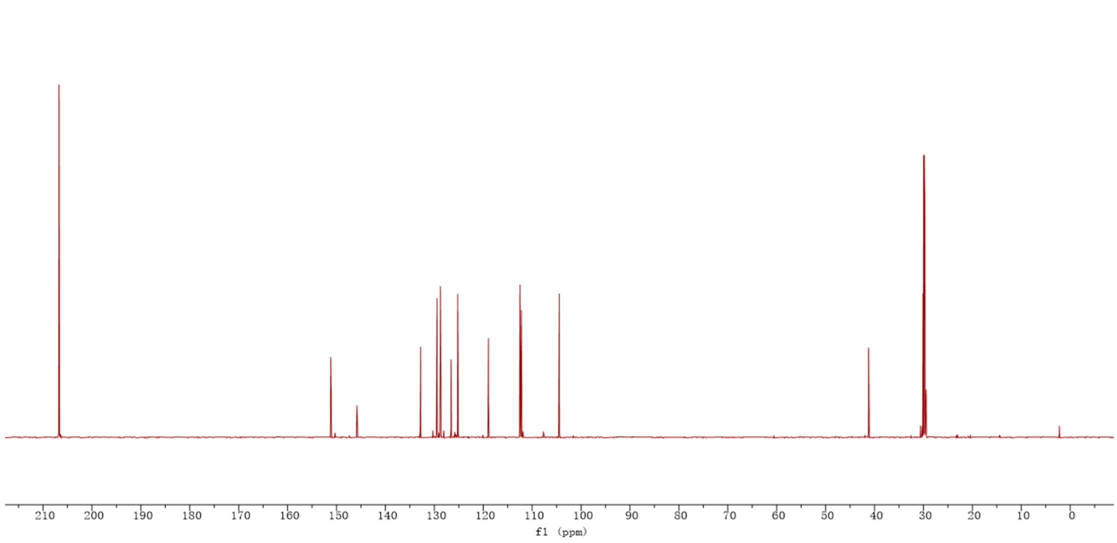

^{13}C NMR (600MHz, DMSO)

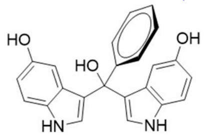

5a

^{1}H NMR (600MHz, Acetone)

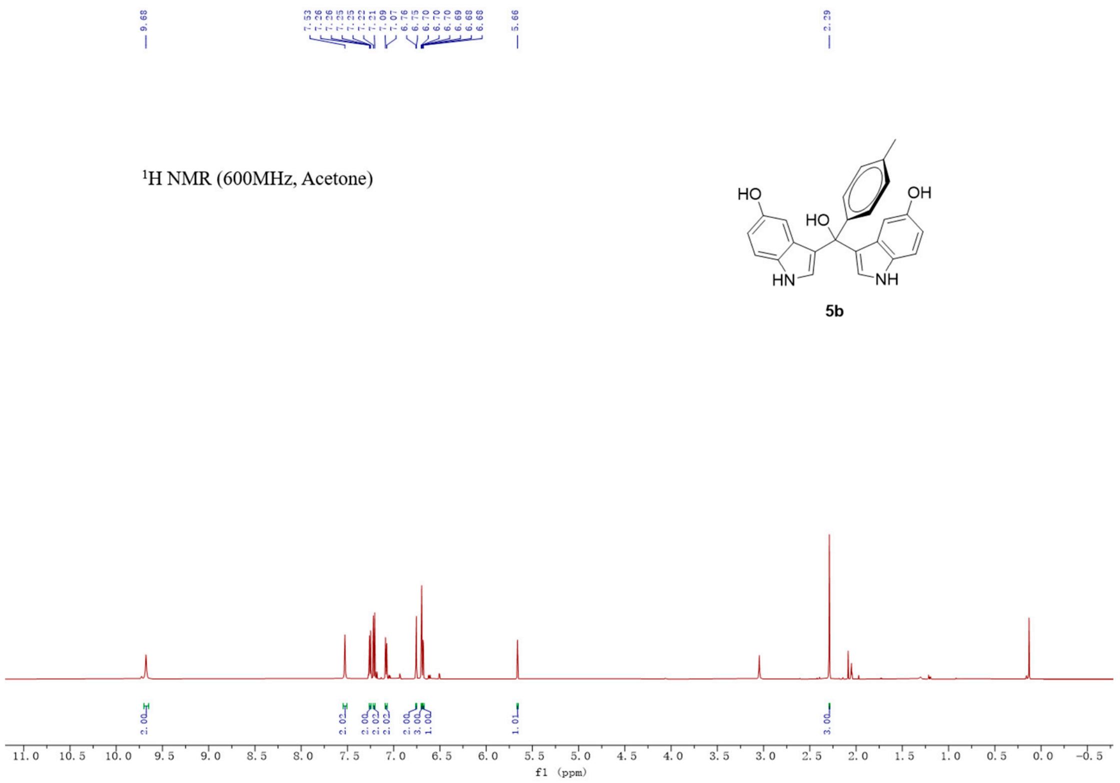

5b

^{13}C NMR (150MHz,Acetone)

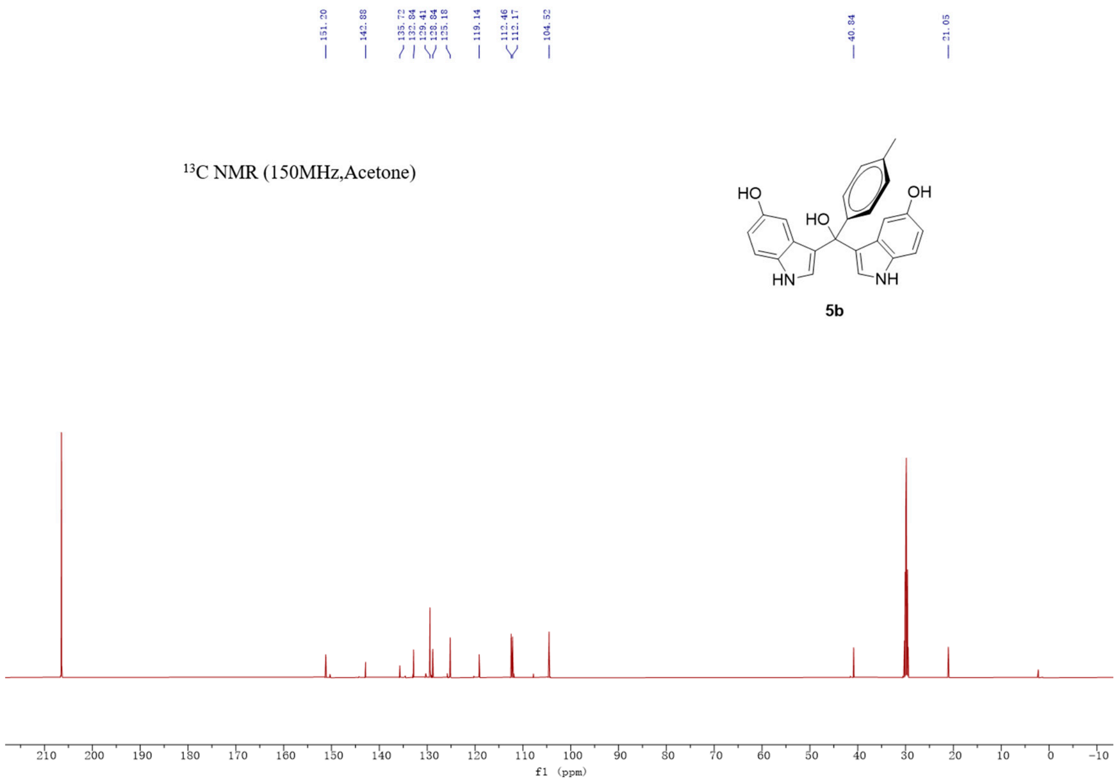

5b

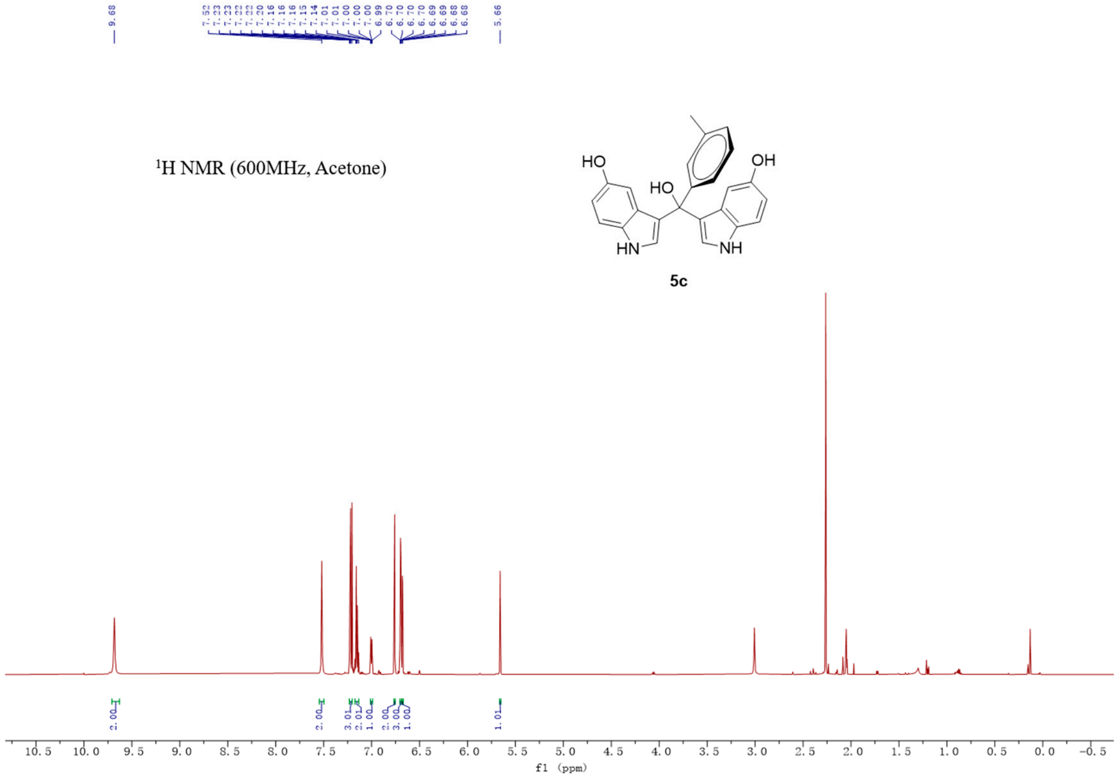
9.68
7.52 7.23 7.23 7.22 7.22 7.20 7.16 7.16 7.16 7.15 7.14 7.01 7.01 7.00 7.00 7.00 6.99 6.70 6.70 6.70 6.70 6.69 6.69 6.68 6.68
5.66
1H NMR (600MHz, Acetone)
HO
OH
HO
HN
NH
5c
2.00
2.00
3.01
2.01
1.00
2.00
3.00
1.00
1.01
f1 (ppm)

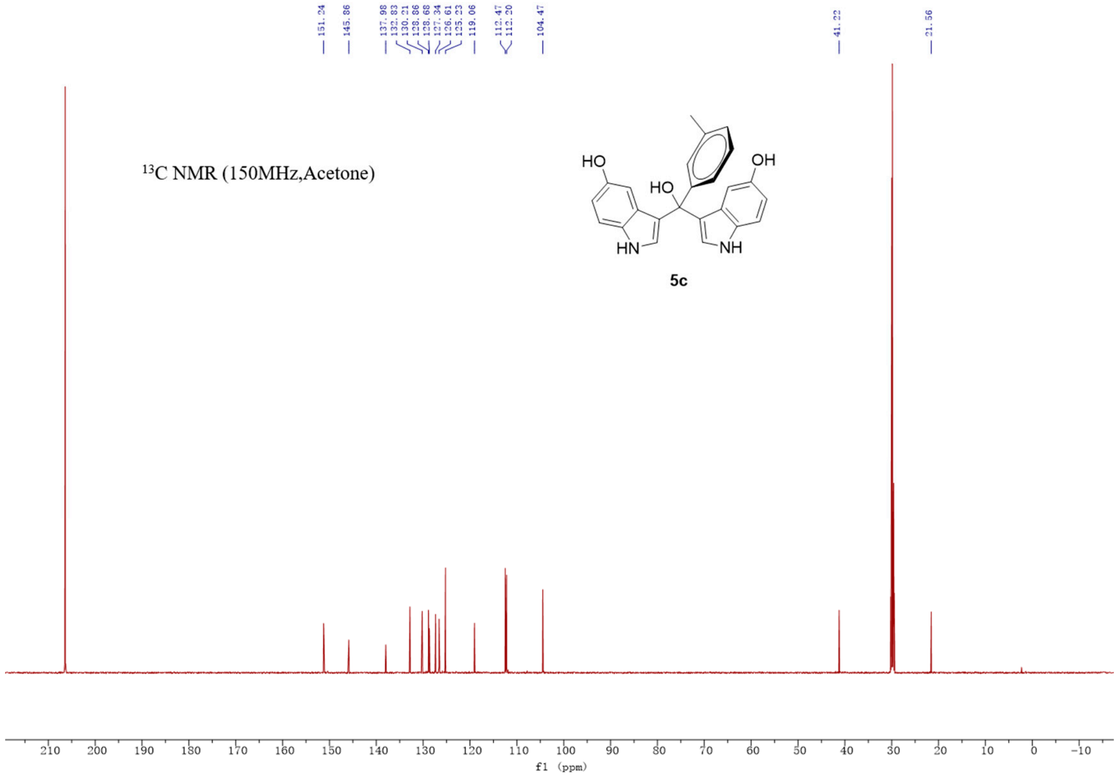
151.24
145.86
137.98 132.83 130.21 128.86 128.68 127.34 126.61 125.23 119.06
112.47 112.20
104.47
41.22
21.56
13C NMR (150MHz,Acetone)
HO
OH
HO
HN
NH
5c
f1 (ppm)

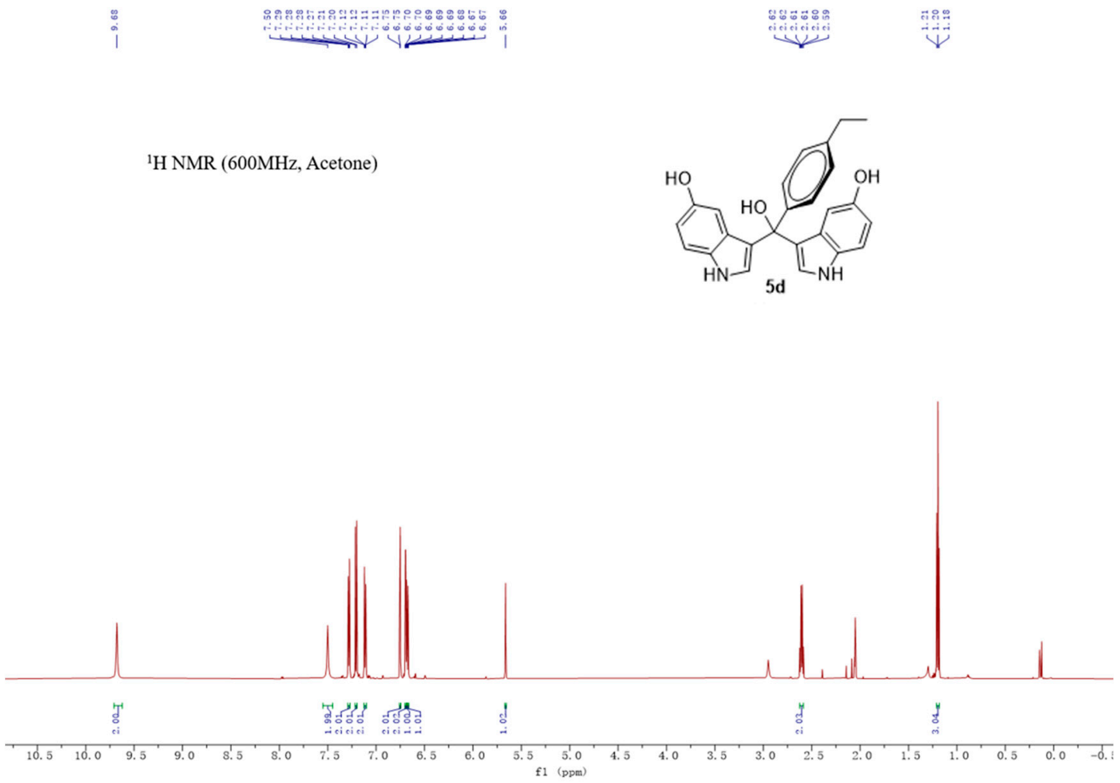

¹H NMR (600MHz, Acetone)
HO
HO
OH
HN
NH
5d
f1 (ppm)

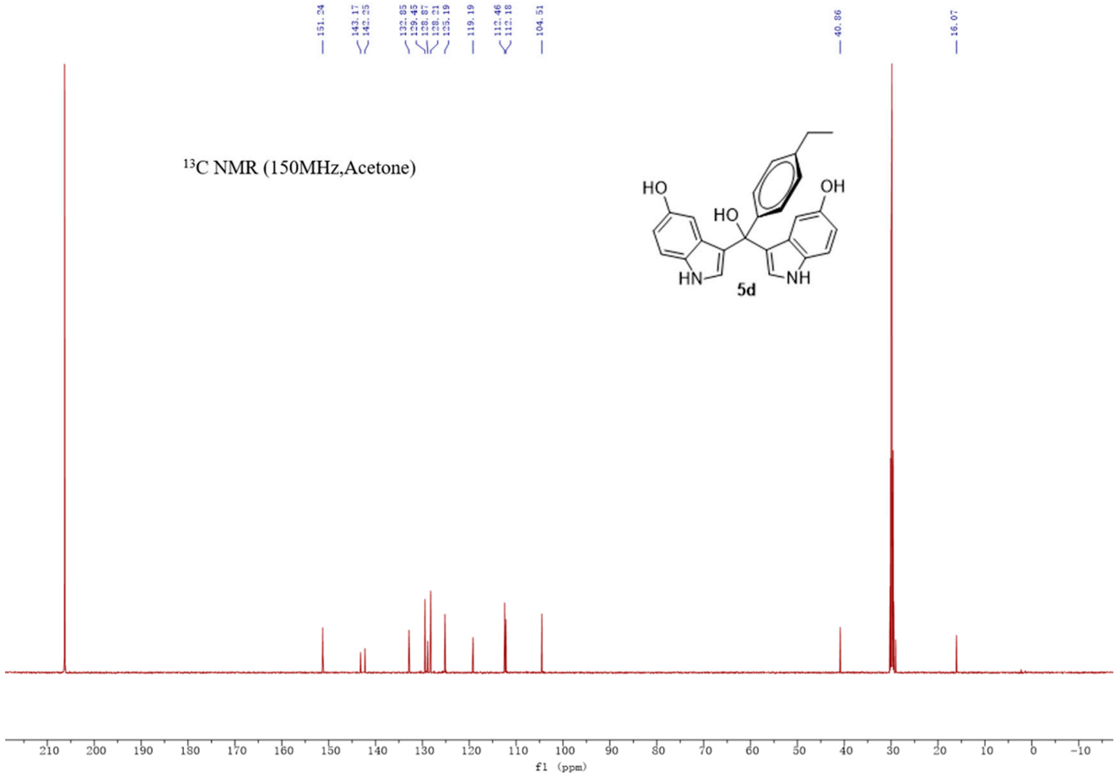

¹³C NMR (150MHz,Acetone)
HO
HO
OH
HN
NH
5d
f1 (ppm)

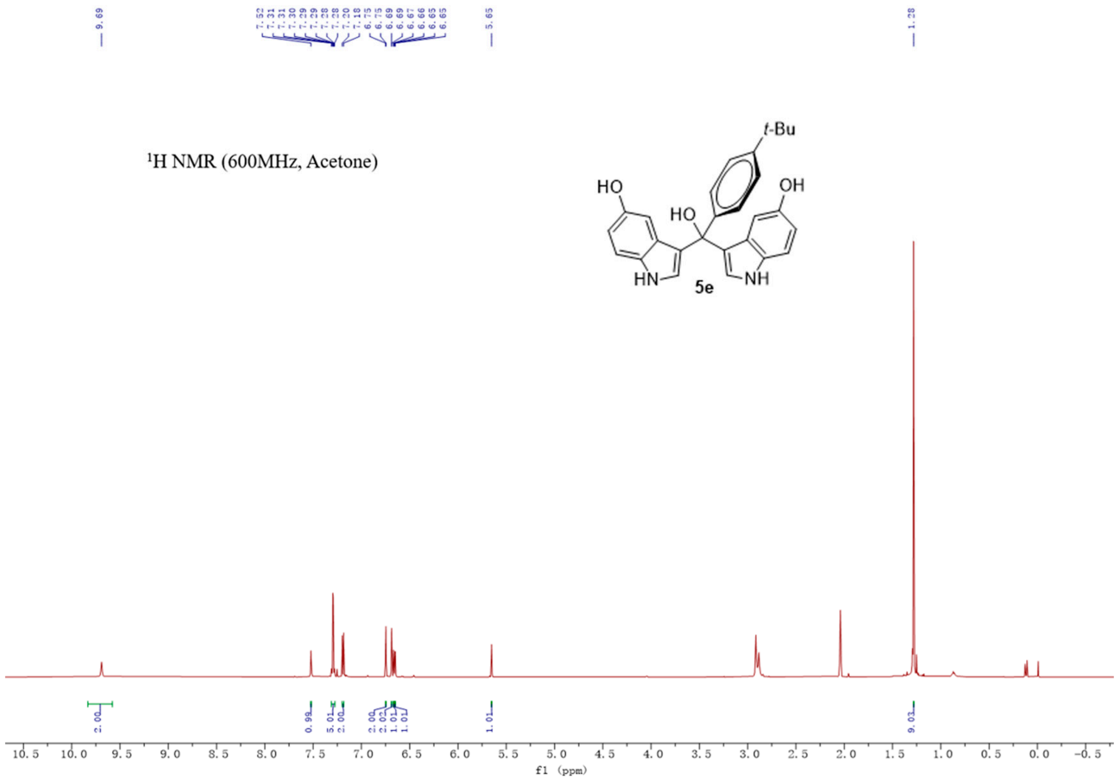
9.69
5.65
1.28
1H NMR (600MHz, Acetone)
t-Bu
HO
OH
HO
HN
NH
5e
f1 (ppm)

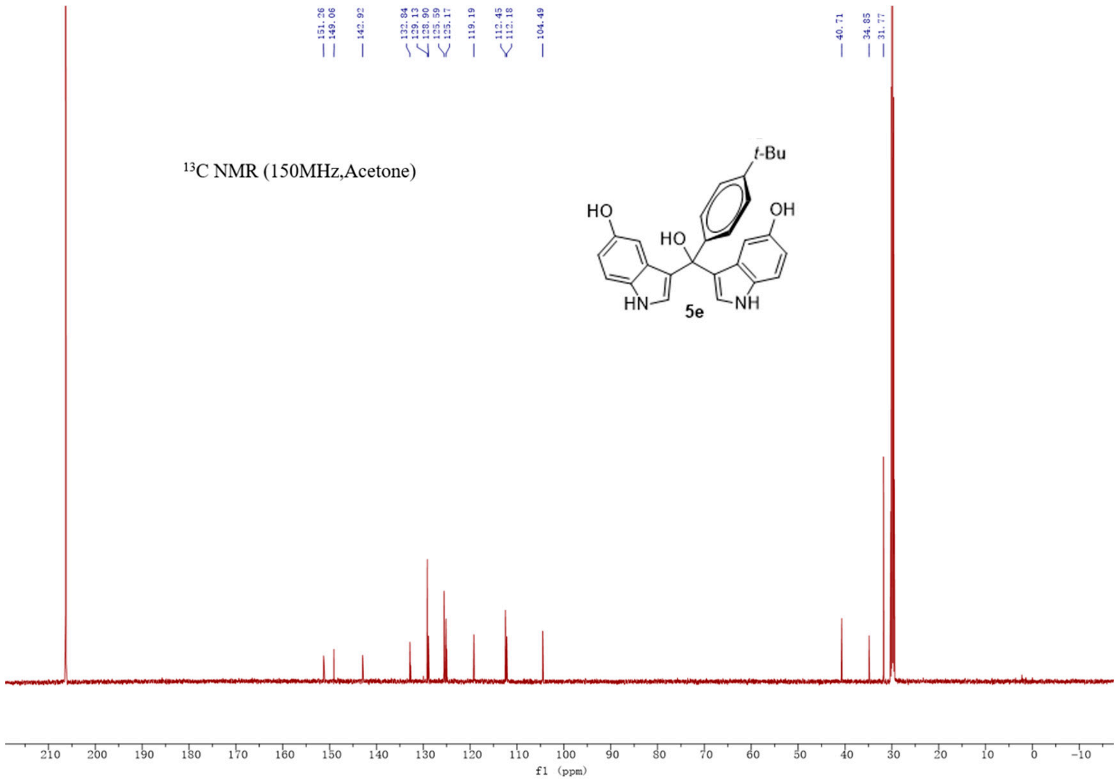
151.26
149.06
142.92
132.84
129.13
128.90
125.59
125.17
119.19
112.45
112.18
104.49
40.71
34.85
31.77
13C NMR (150MHz,Acetone)
t-Bu
HO
OH
HO
HN
NH
5e
f1 (ppm)

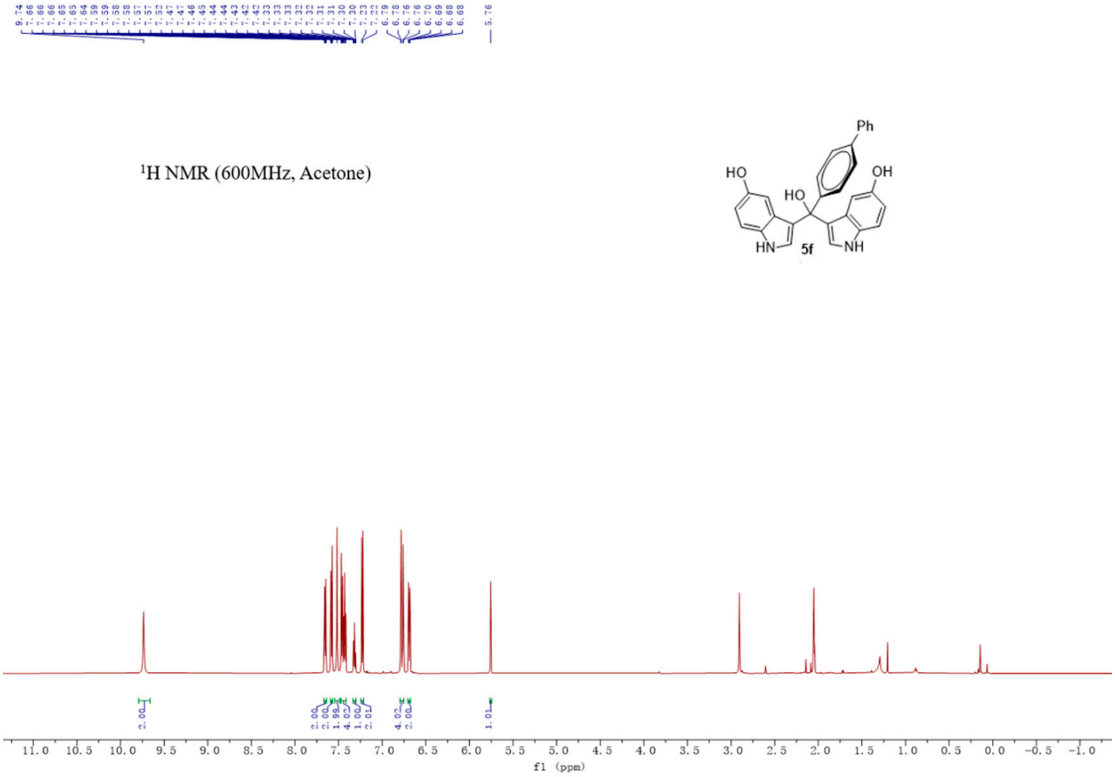

1H NMR (600MHz, Acetone)
Ph
HO
HO
OH
HN
NH
5f
f1 (ppm)

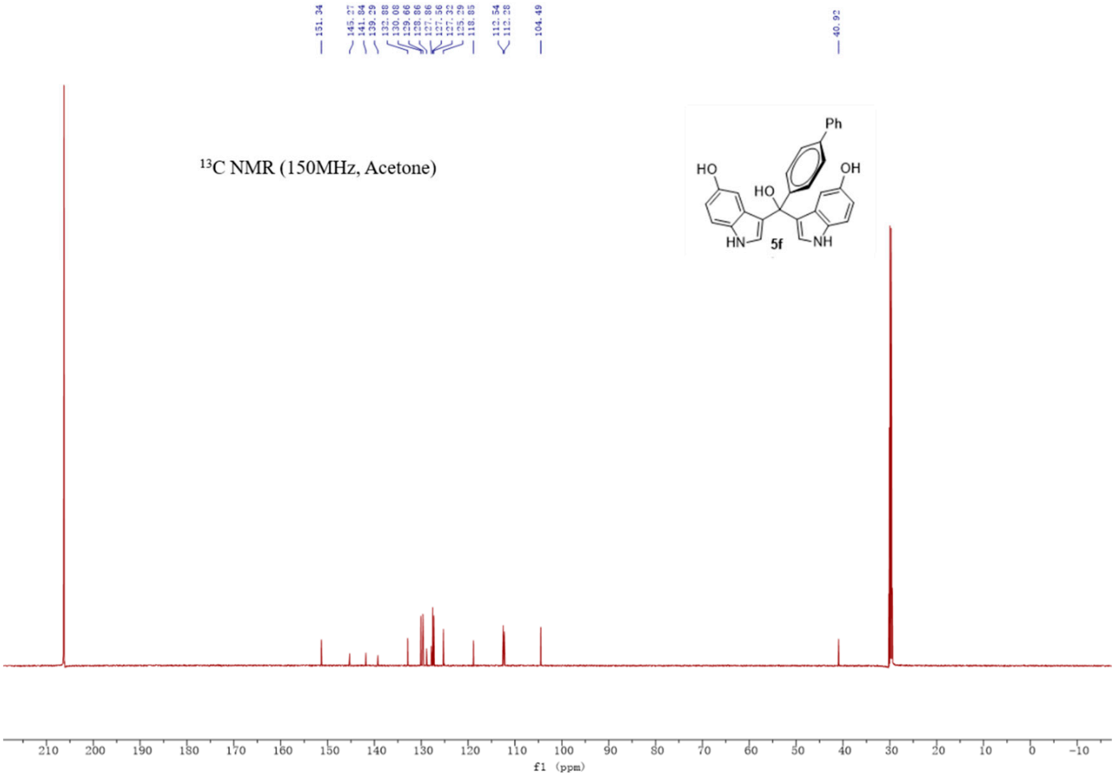

151.34
145.27
141.84
139.29
132.88
130.08
129.66
128.86
127.86
127.56
127.32
125.29
118.85
112.54
112.28
104.49
40.92
13C NMR (150MHz, Acetone)
Ph
HO
HO
OH
HN
NH
5f
f1 (ppm)

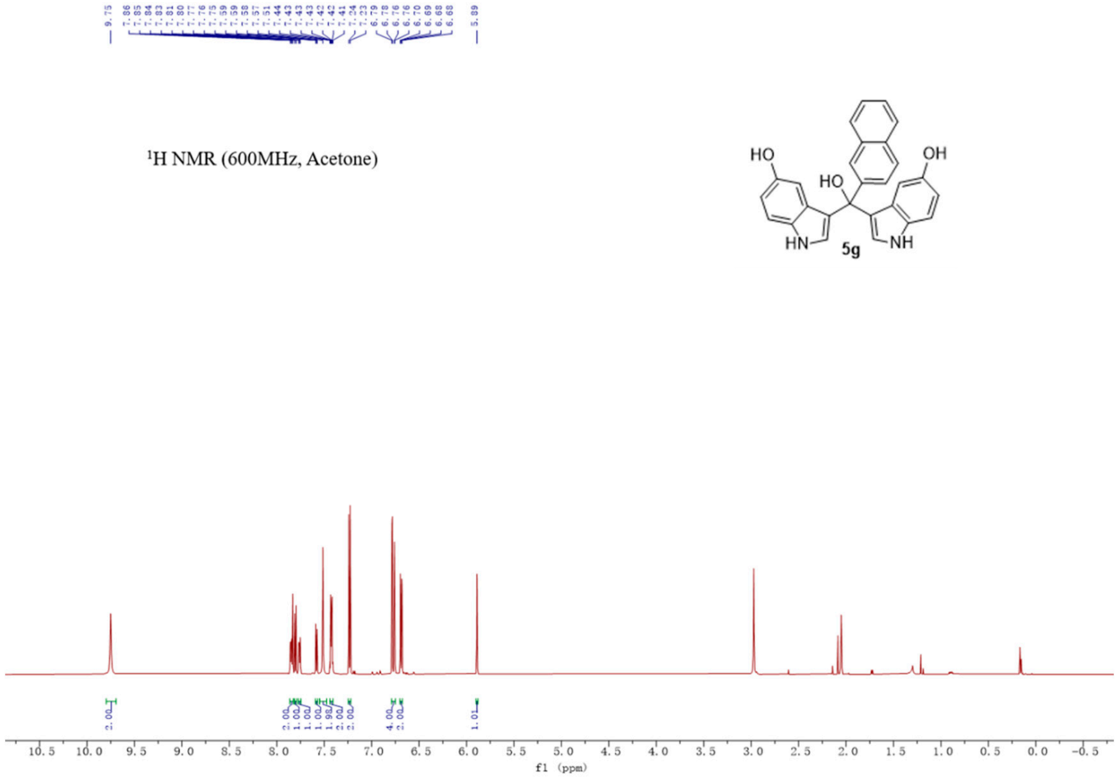

1H NMR (600MHz, Acetone)
HO
HO
OH
HN
NH
5g
f1 (ppm)

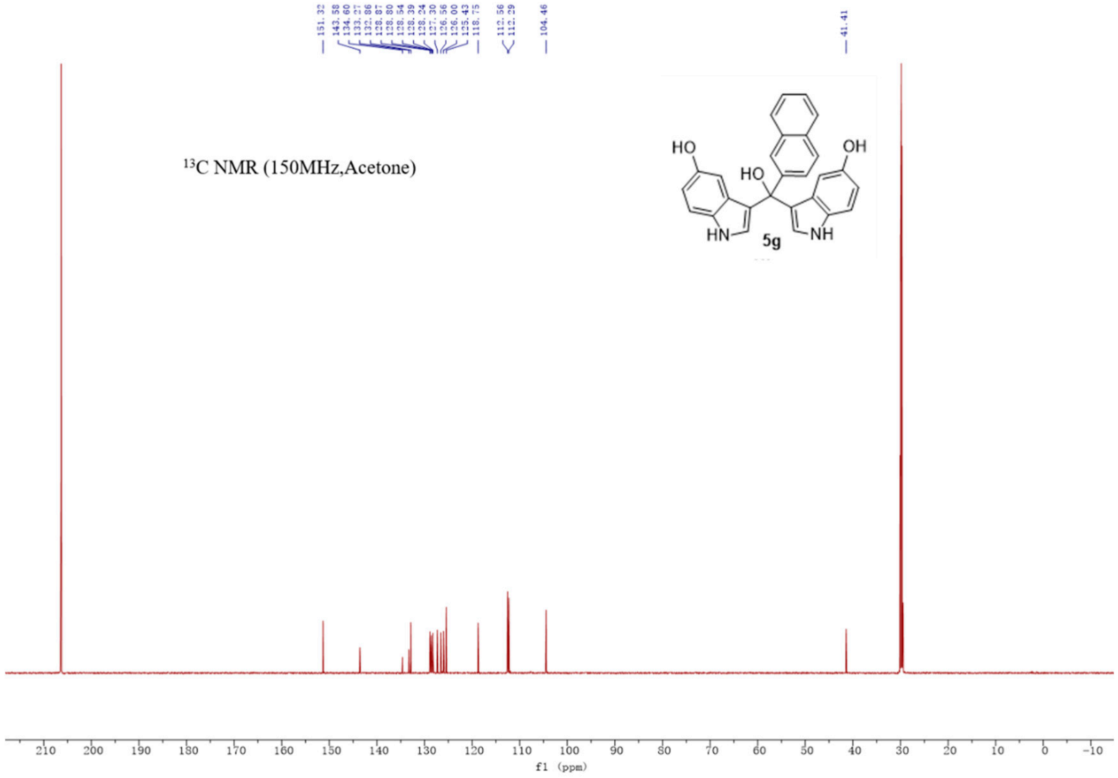

13C NMR (150MHz,Acetone)
HO
HO
OH
HN
NH
5g
f1 (ppm)

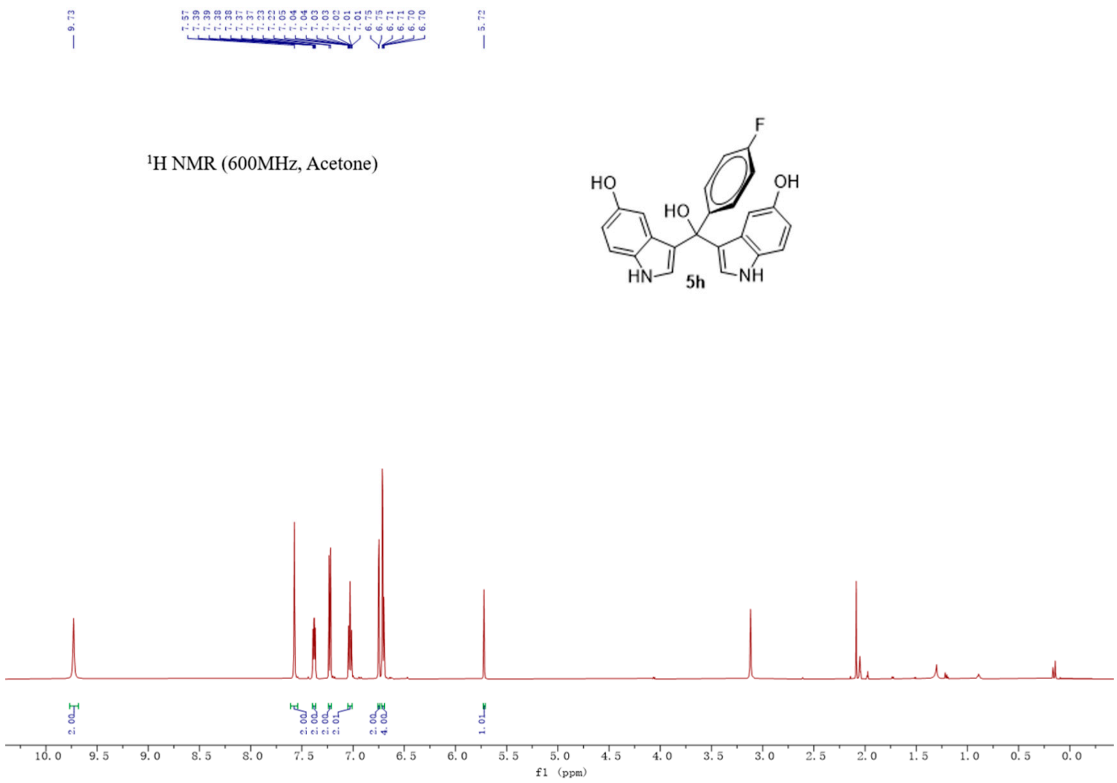
1H NMR (600MHz, Acetone)
5h
f1 (ppm)

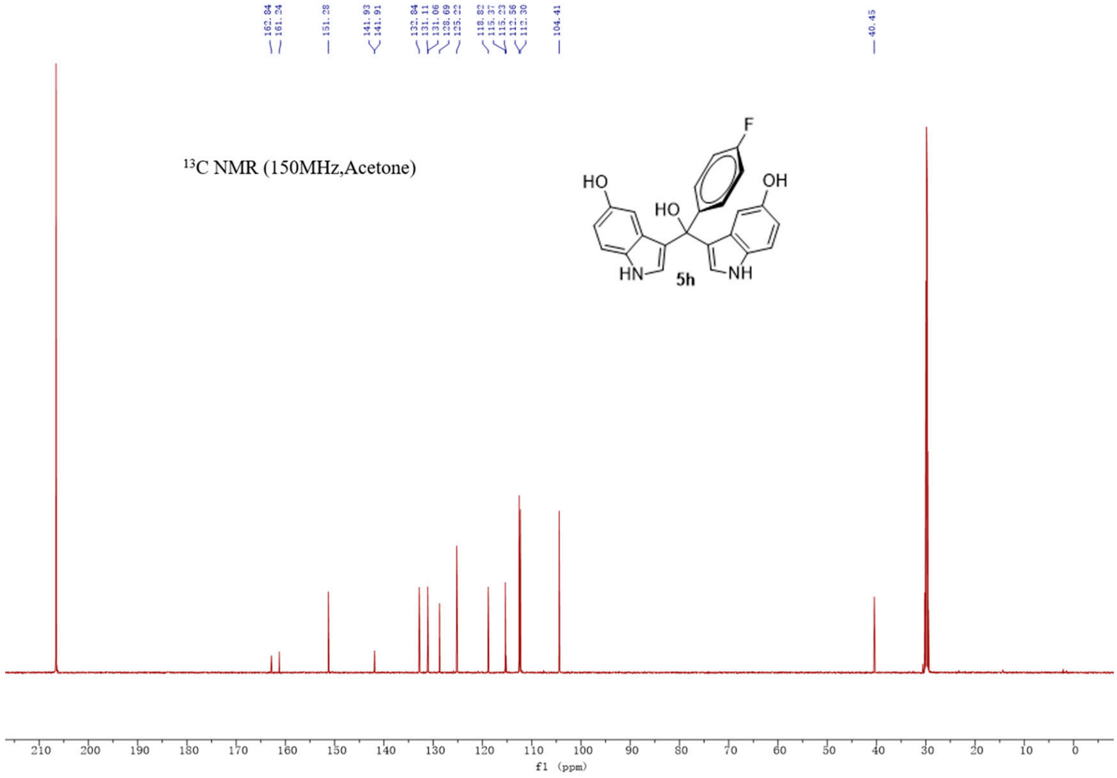
13C NMR (150MHz,Acetone)
5h
f1 (ppm)

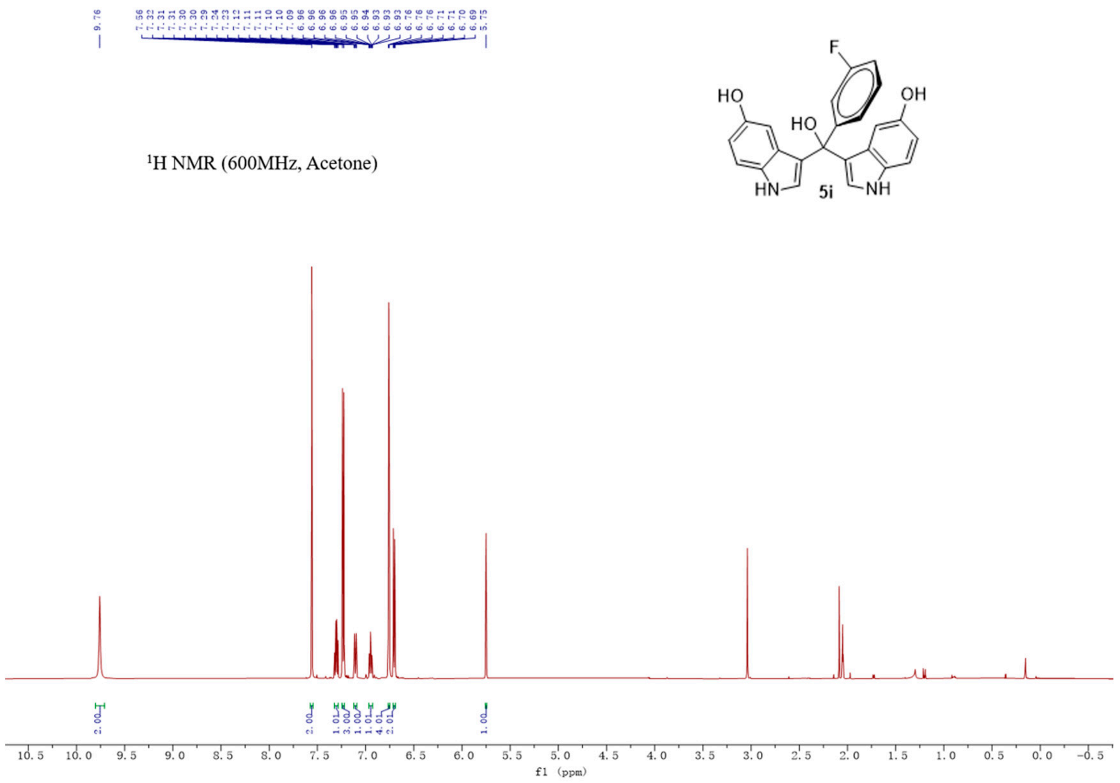

^{1}H NMR (600MHz, Acetone)
5i
f1 (ppm)

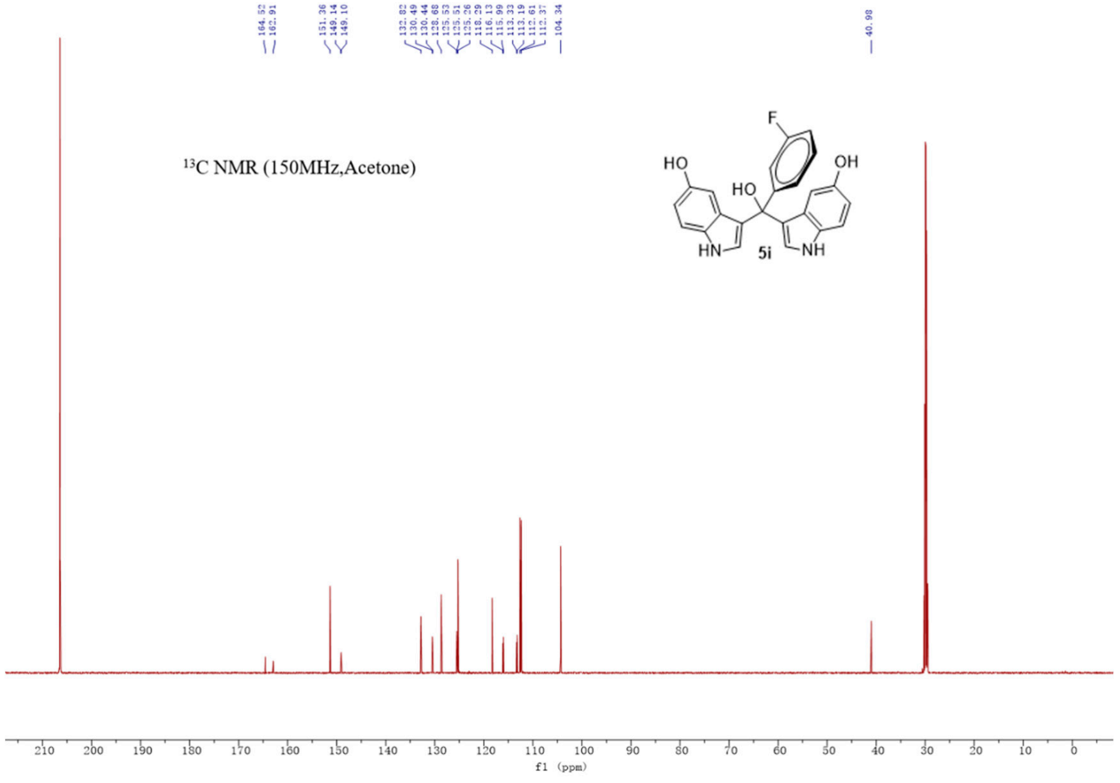

^{13}C NMR (150MHz,Acetone)
5i
f1 (ppm)

^{1}H NMR (600 MHZ, acetone-d_6)

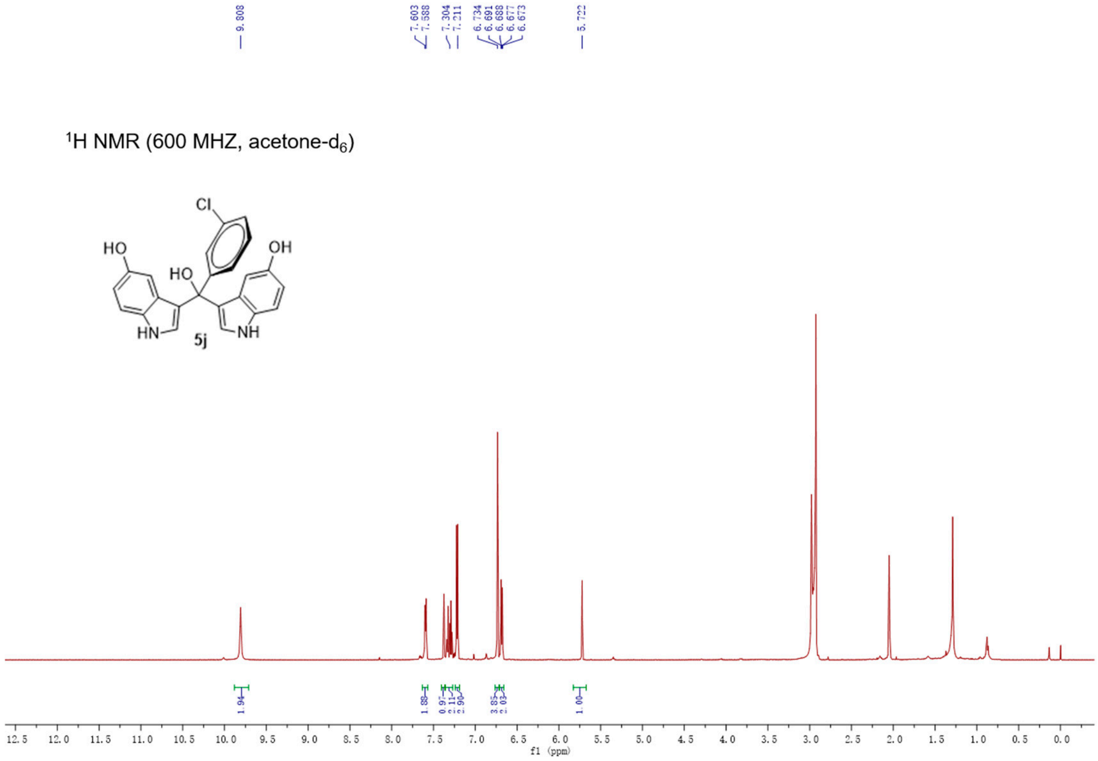

[13] C NMR (600 MHZ, acetone-d_6)

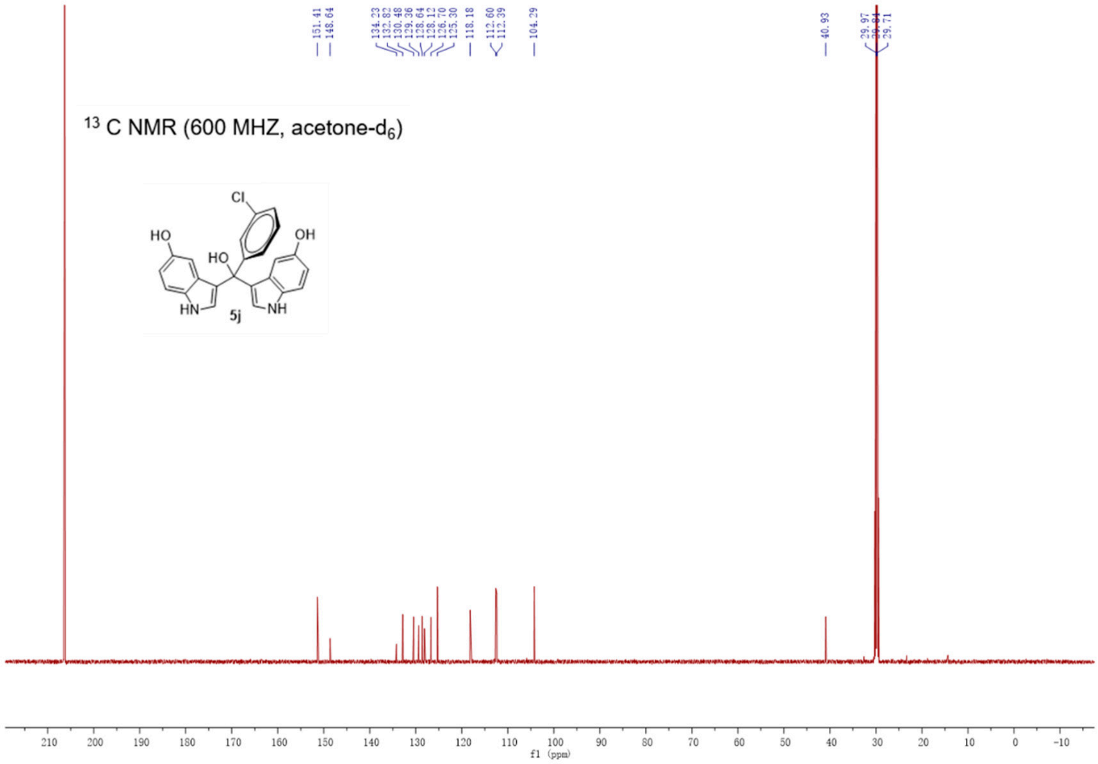

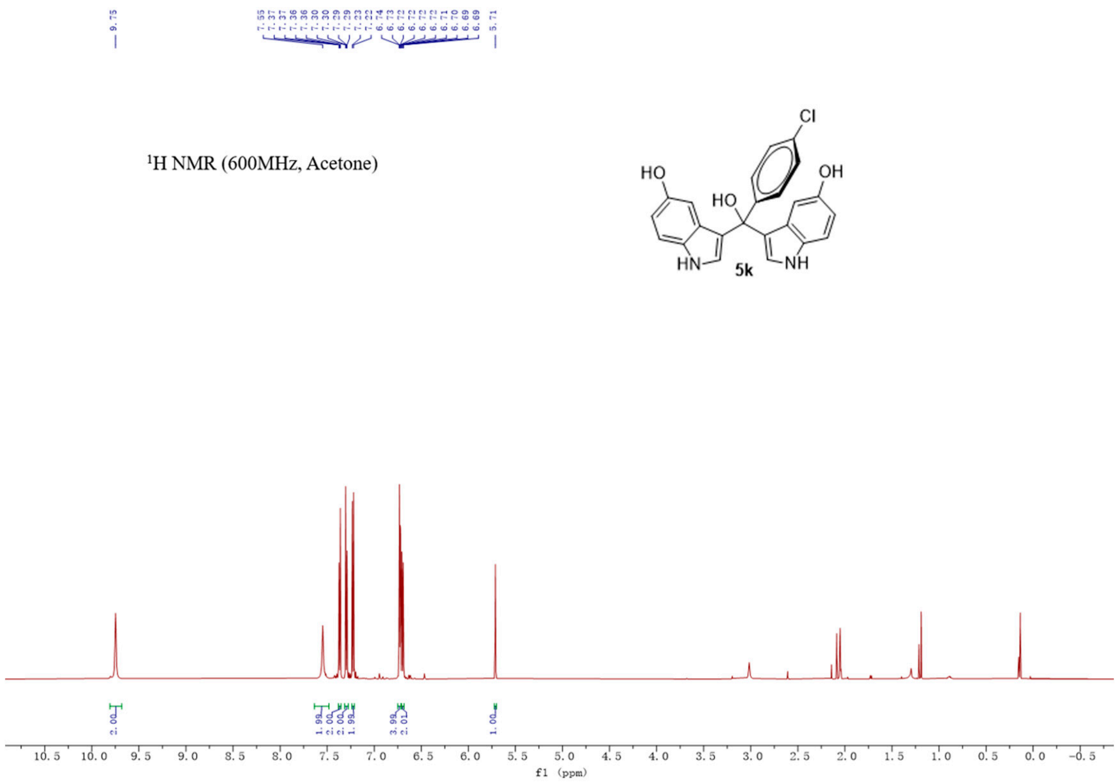

1H NMR (600MHz, Acetone)
5k
f1 (ppm)

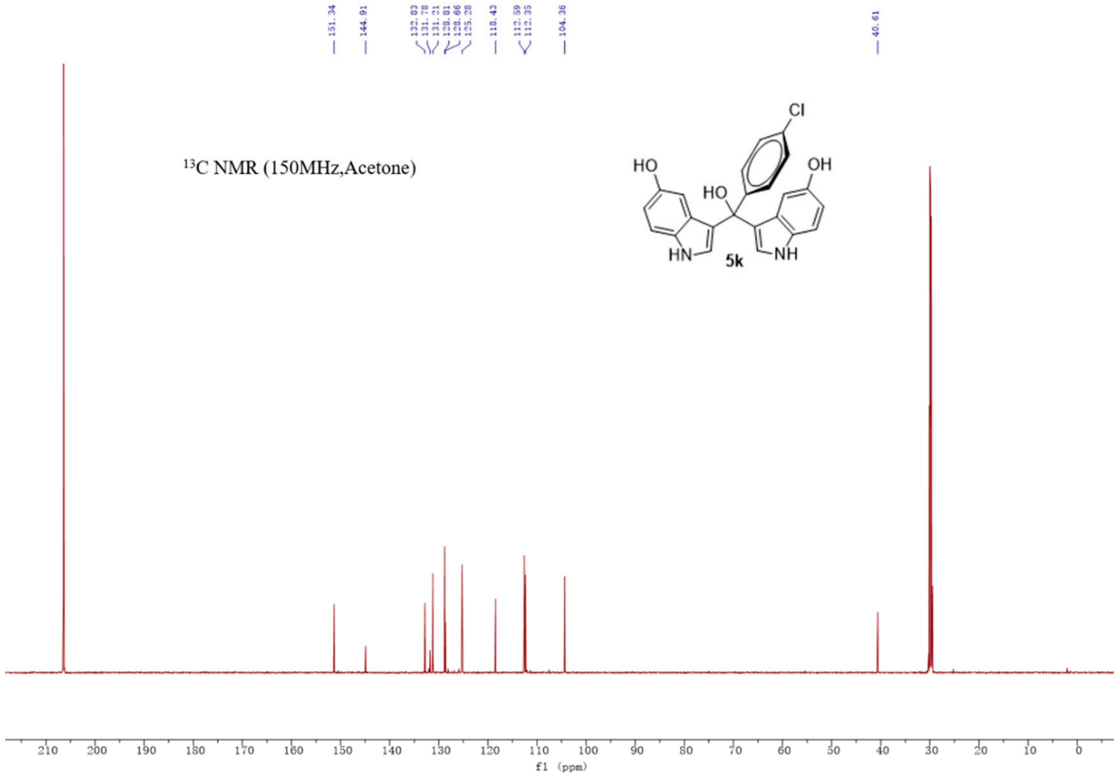

13C NMR (150MHz,Acetone)
5k
f1 (ppm)

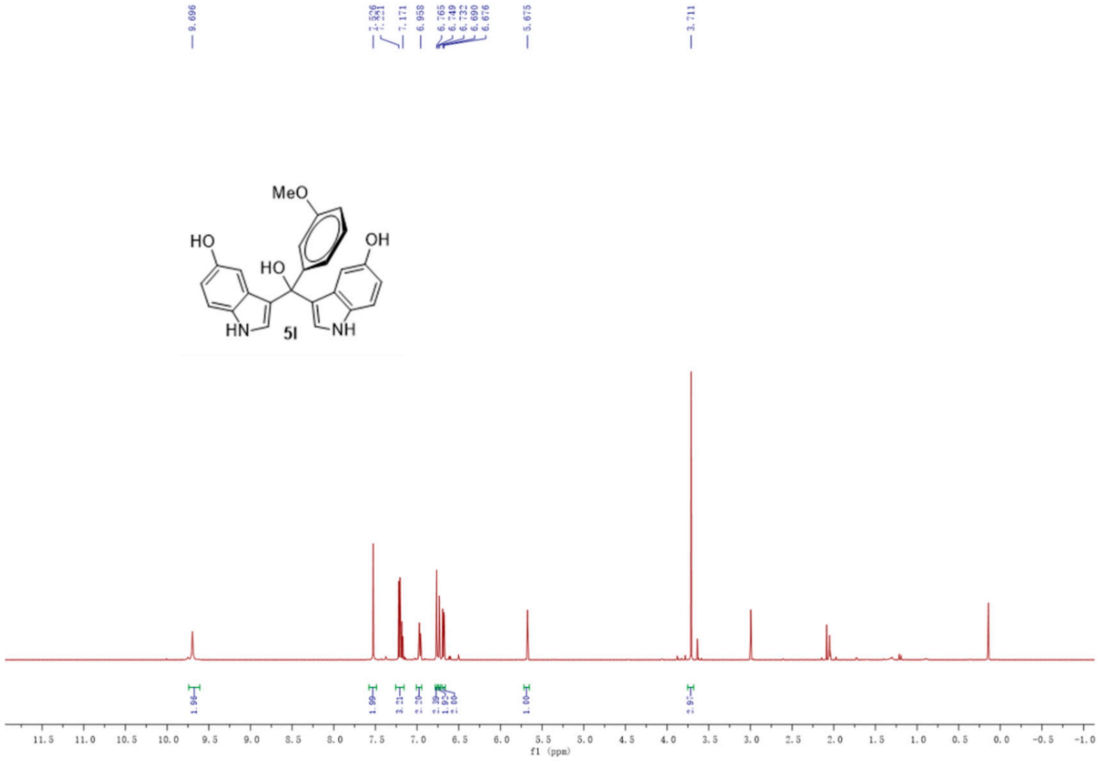
9.696
7.526
7.171
6.958
6.765
6.749
6.732
6.690
6.676
5.675
3.711
MeO
HO
OH
HO
HN
5l
NH
f1 (ppm)

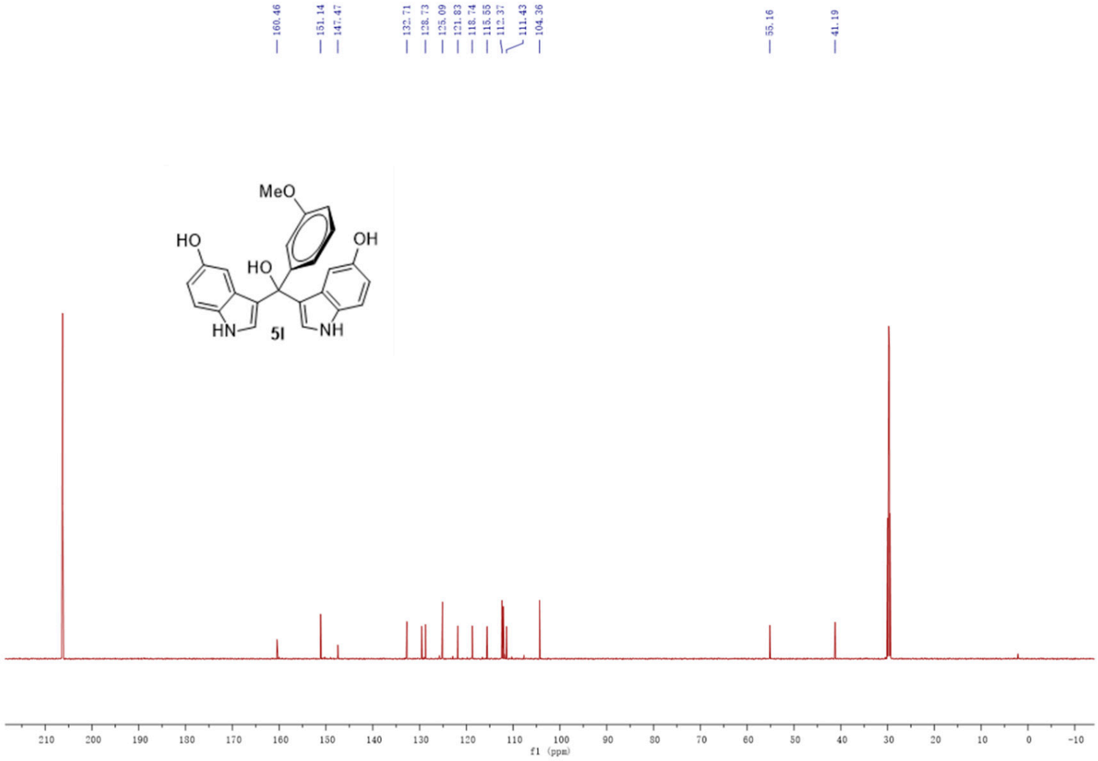
160.46
151.14
147.47
132.71
128.73
125.09
121.83
118.74
115.55
112.37
111.43
104.36
55.16
41.19
MeO
HO
OH
HO
HN
5l
NH
f1 (ppm)

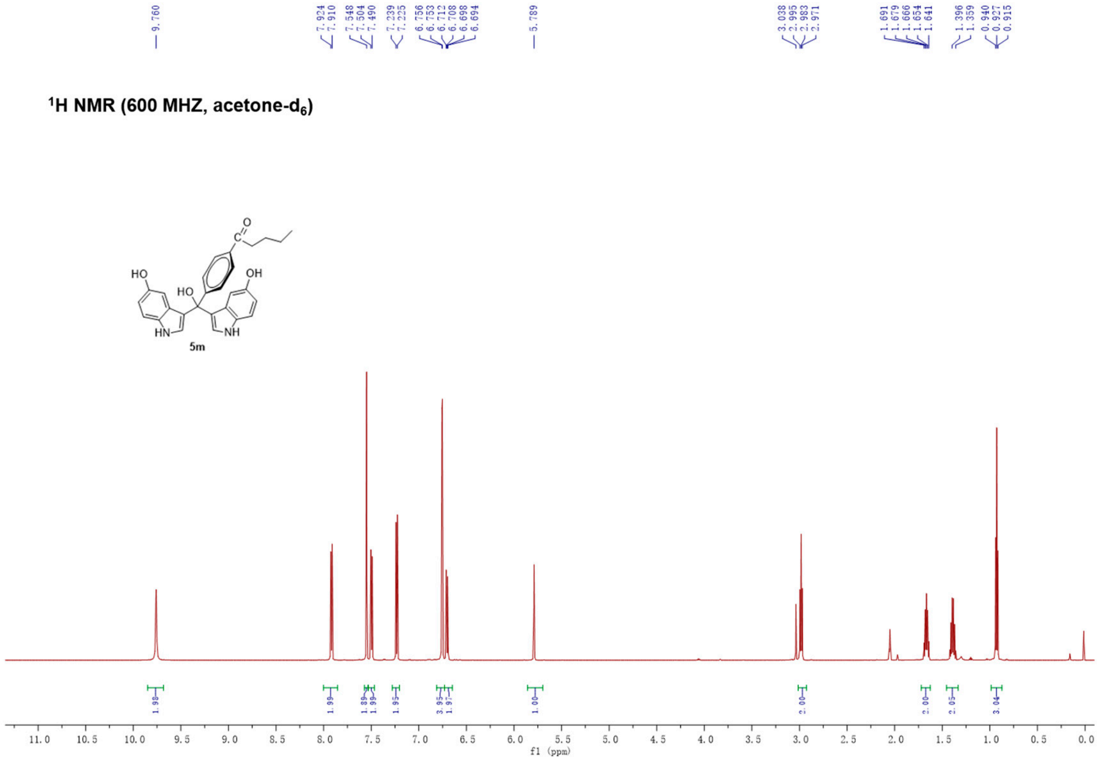

9.760
7.924
7.910
7.548
7.504
7.490
7.239
7.225
6.756
6.753
6.712
6.708
6.698
6.694
5.789
3.038
2.995
2.983
2.971
1.691
1.679
1.666
1.654
1.641
1.396
1.359
0.940
0.927
0.915
¹H NMR (600 MHZ, acetone-d6)
HO
HO
OH
O
HN
NH
5m
1.98
1.99
1.89
1.99
1.95
3.95
1.97
1.00
2.00
2.00
2.05
3.04
f1 (ppm)

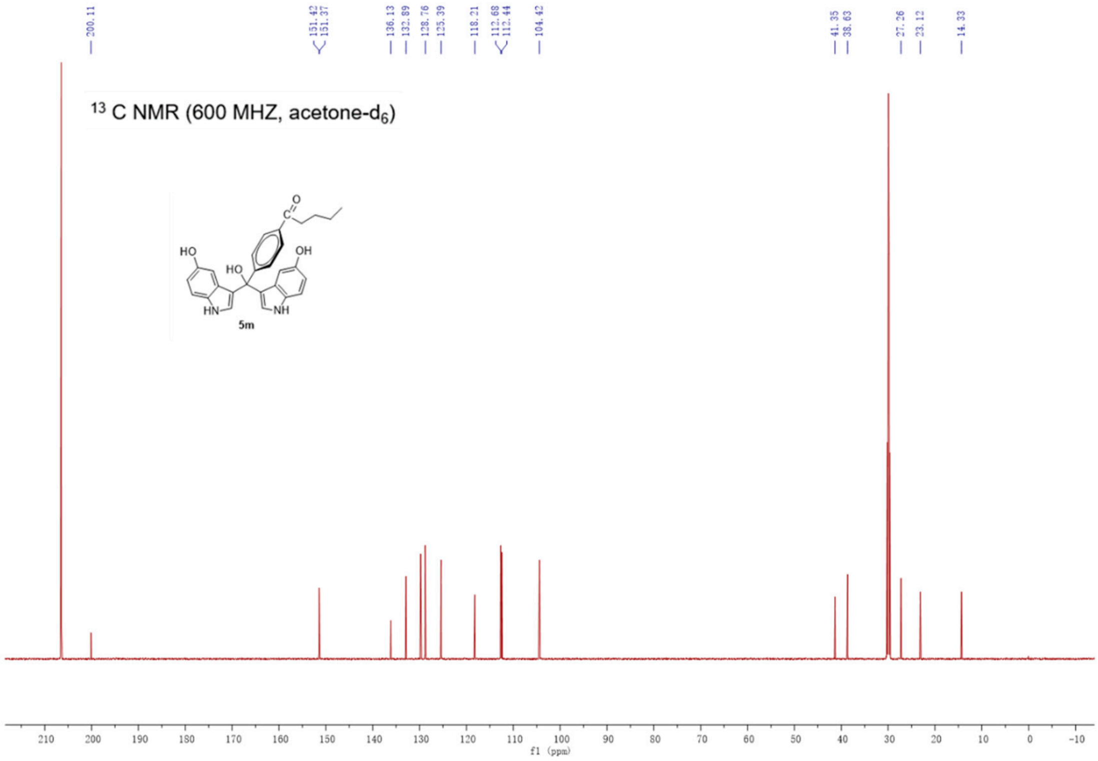

200.11
151.42
151.37
136.13
132.89
128.76
125.39
118.21
112.68
112.44
104.42
41.35
38.63
27.26
23.12
14.33
¹³ C NMR (600 MHZ, acetone-d6)
HO
HO
OH
O
HN
NH
5m
f1 (ppm)

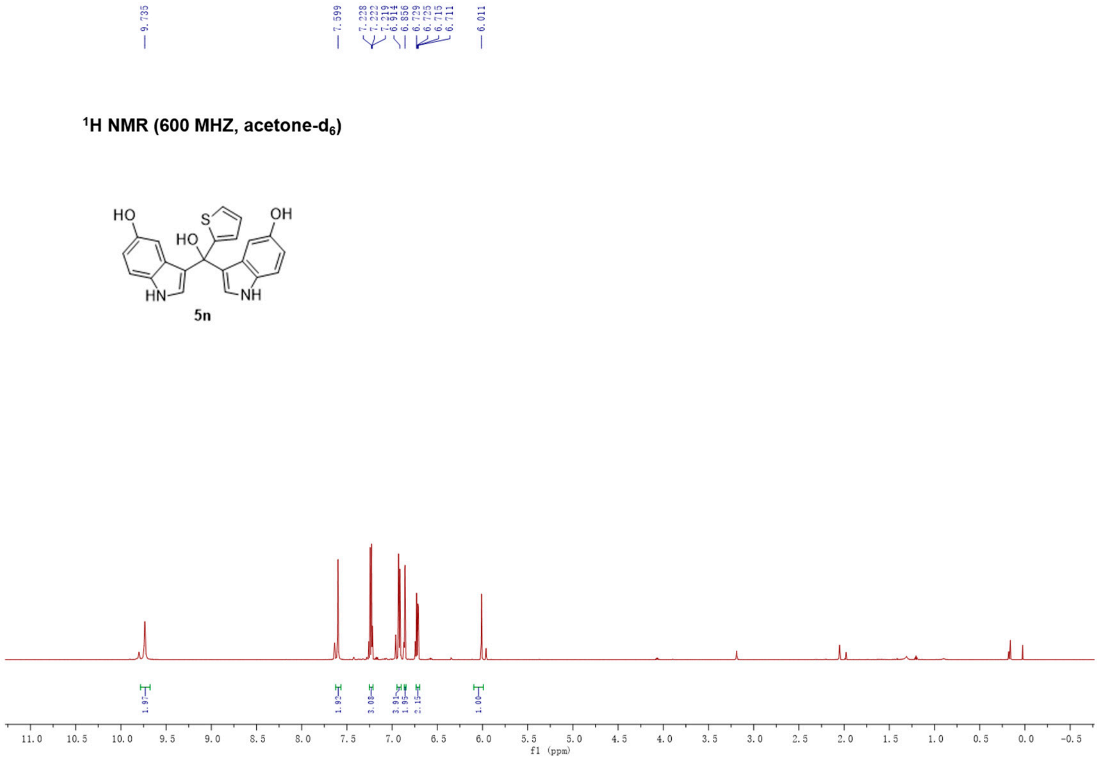

9.735
7.599
7.228
7.222
7.219
6.914
6.856
6.729
6.725
6.715
6.711
6.011
1H NMR (600 MHZ, acetone-d6)
HO
OH
HO
S
HN
NH
5n
1.97
1.95
3.08
3.91
1.95
2.12
1.00
f1 (ppm)

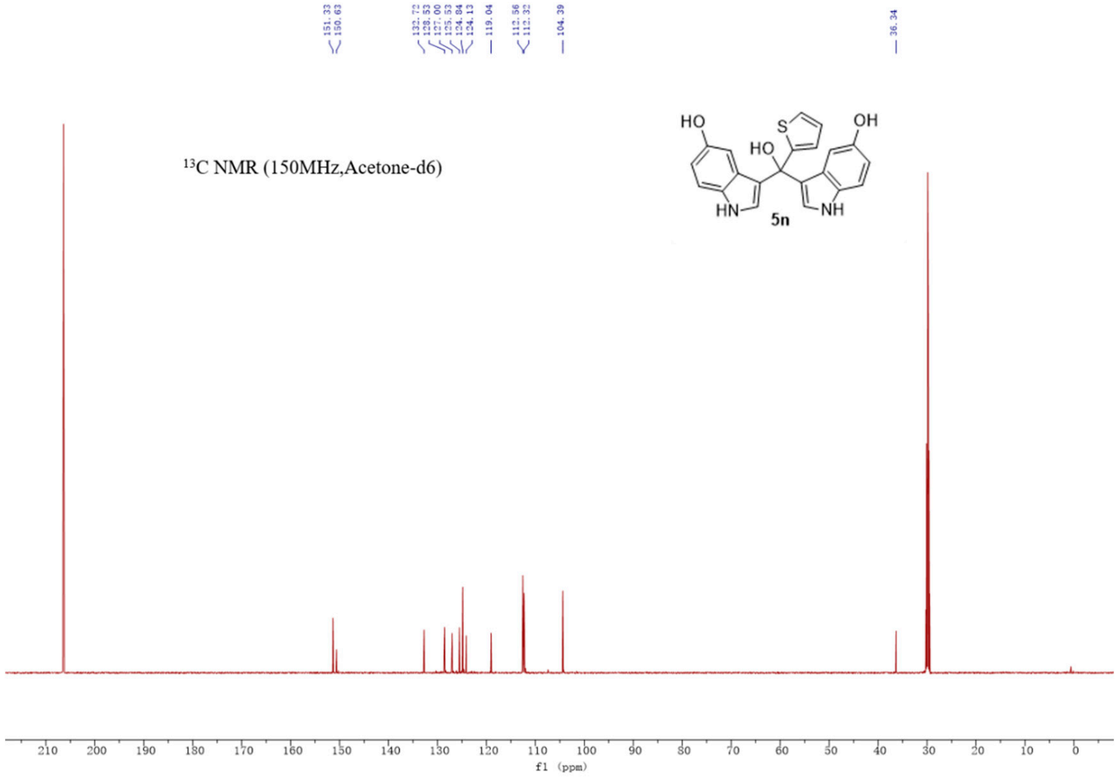

151.23
150.63
132.72
128.53
127.00
125.53
124.84
124.13
119.04
112.56
112.32
104.39
36.34
13C NMR (150MHz,Acetone-d6)
HO
OH
HO
S
HN
NH
5n
f1 (ppm)

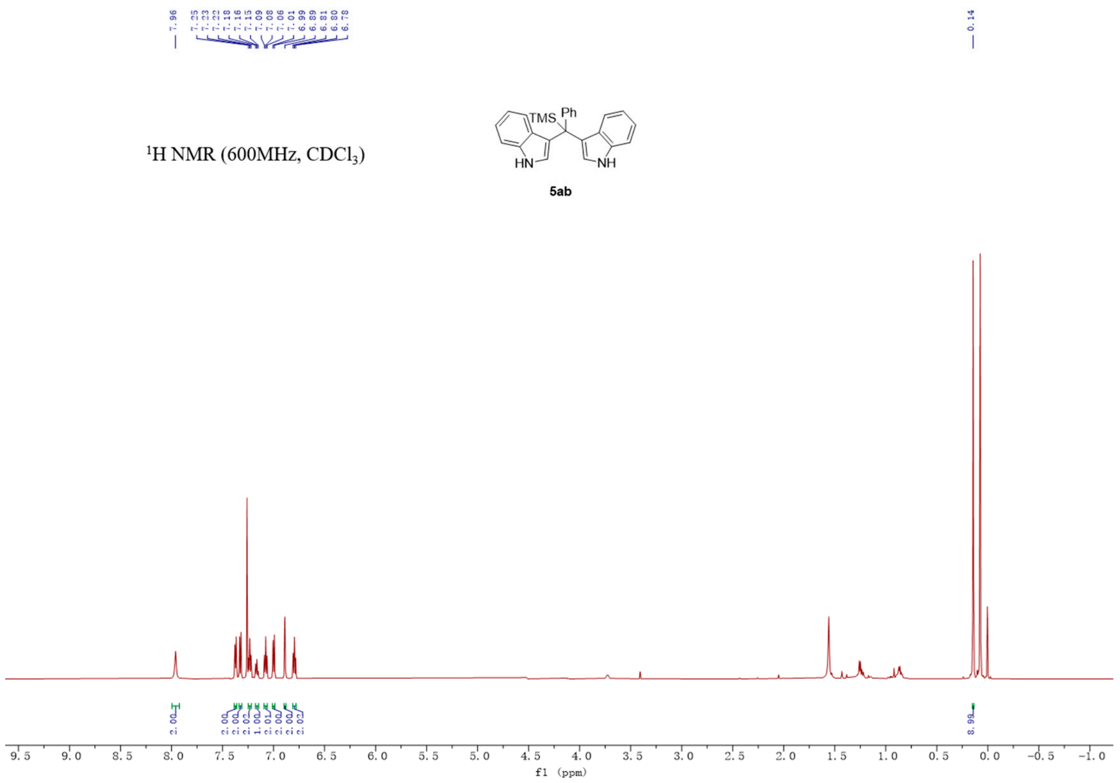
7.96
7.35 7.33 7.22 7.18 7.16 7.15 7.09 7.08 7.06 7.01 6.99 6.89 6.81 6.80 6.78
0.14
1H NMR (600MHz, CDCl3)
TMS
Ph
HN
NH
5ab
f1 (ppm)

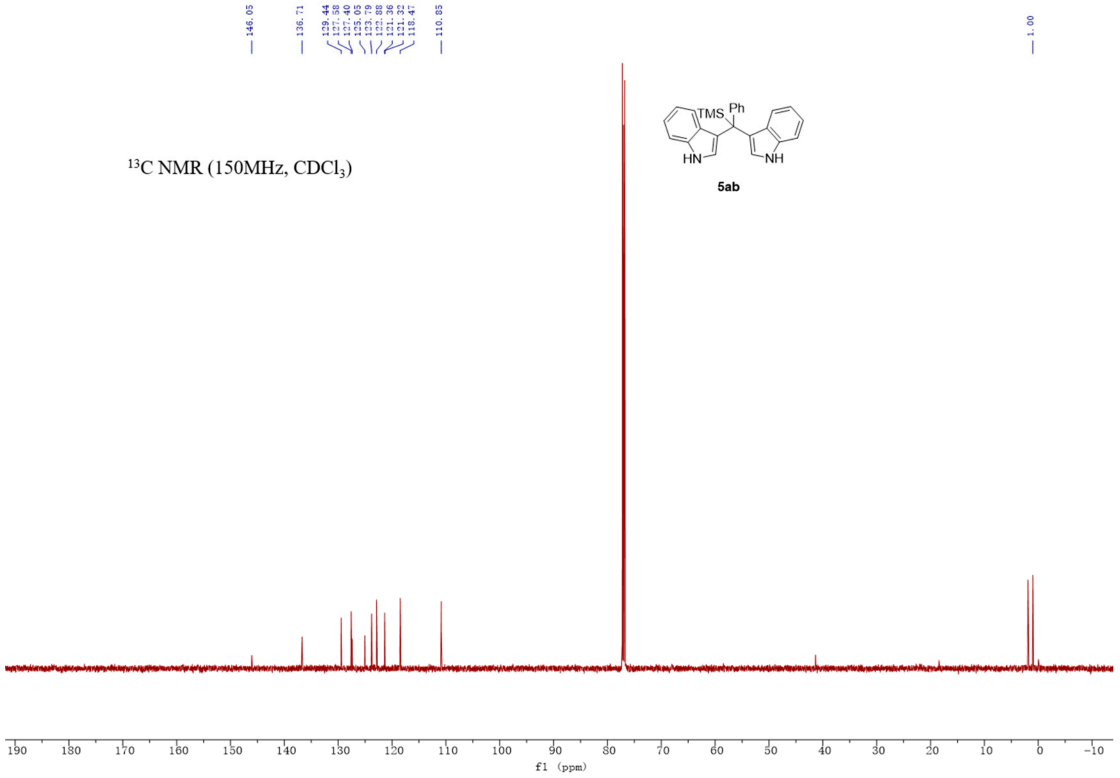
146.05
136.71
129.44 127.58 127.40 125.05 123.79 122.88 121.36 121.32 118.47
110.85
1.00
13C NMR (150MHz, CDCl3)
TMS
Ph
HN
NH
5ab
f1 (ppm)

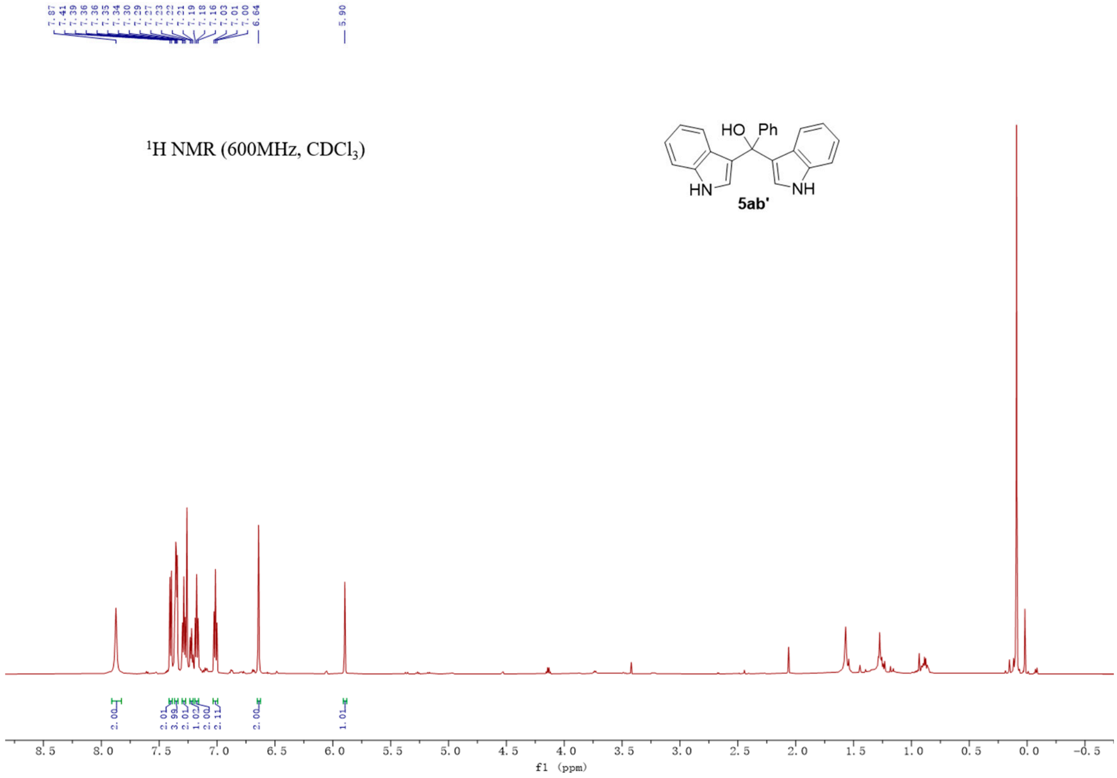
7.87 7.41 7.39 7.36 7.36 7.35 7.34 7.30 7.29 7.27 7.23 7.22 7.21 7.19 7.18 7.16 7.03 7.01 7.00 6.64
5.90
^{1}H NMR (600MHz, CDCl$_3$)
HO Ph
HN NH
5ab'
2.00 2.01 3.99 2.01 1.01 2.00 2.11 2.00 1.01
8.5 8.0 7.5 7.0 6.5 6.0 5.5 5.0 4.5 4.0 3.5 3.0 2.5 2.0 1.5 1.0 0.5 0.0 -0.5
f1 (ppm)

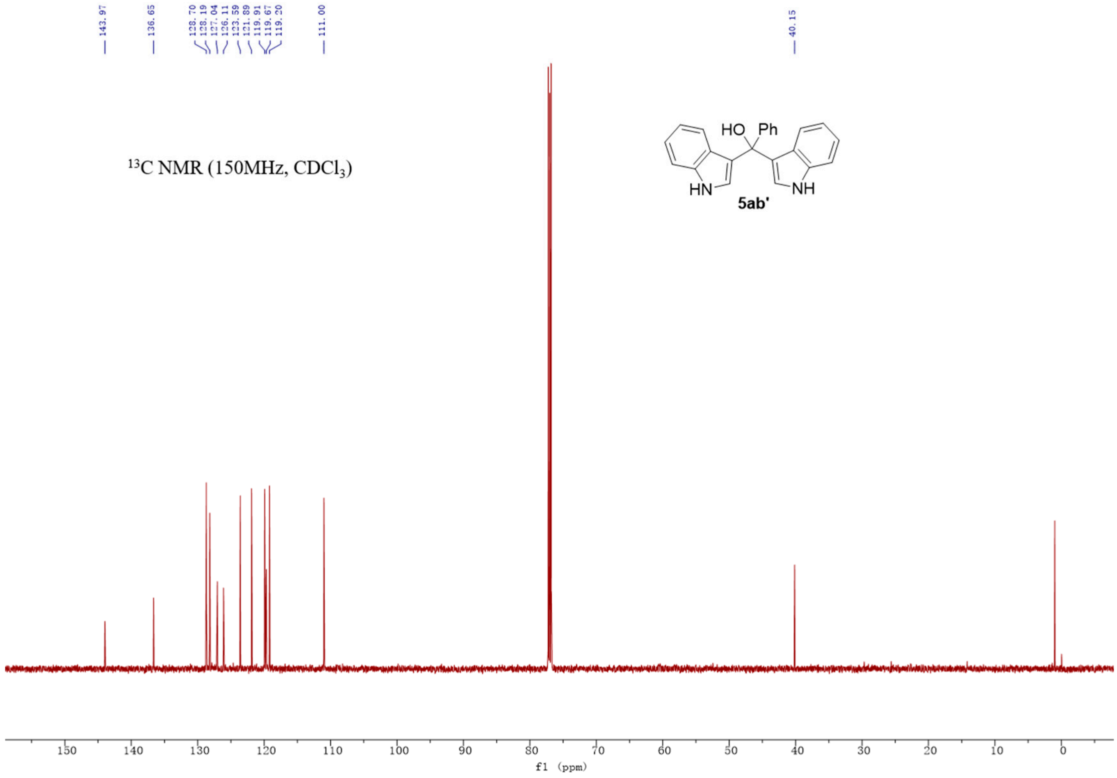
143.97
136.65
128.70 128.19 127.04 126.11 123.59 121.89 119.91 119.67 119.20
111.00
40.15
^{13}C NMR (150MHz, CDCl$_3$)
HO Ph
HN NH
5ab'
150 140 130 120 110 100 90 80 70 60 50 40 30 20 10 0
f1 (ppm)
